# ABO Antigens Active Tri- and Disaccharides Microarray to Evaluate C-type Lectin Receptor Binding Preferences

**DOI:** 10.1038/s41598-018-24333-y

**Published:** 2018-04-26

**Authors:** Chethan D. Shanthamurthy, Prashant Jain, Sharon Yehuda, João T. Monteiro, Shani Leviatan Ben-Arye, Balamurugan Subramani, Bernd Lepenies, Vered Padler-Karavani, Raghavendra Kikkeri

**Affiliations:** 1 Indian Institute of Science Education and Research, Dr. Homi Bhabha Road, Pune, 411008 India; 2 Tel-Aviv University, Department of Cell Research and Immunology, The George S. Wise Faculty of Life Sciences, Tel-Aviv, 69978 Israel; 3 University of Veterinary Medicine Hannover, Immunology Unit & Research Center for Emerging Infections and Zoonoses, Hannover, Germany

## Abstract

Understanding blood group antigen binding preferences for C-type lectin receptors holds promise for modulating immune responses, since several Gram-negative bacteria express blood group antigens as molecular mimicry to evade immune responses. Herein, we report the synthesis of ABO blood group antigen active tri and disaccharides to investigate the binding specificity with various C-type lectin receptors using glycan microarray. The results of binding preferences show that distinct glycosylation on the galactose and fucose motifs are key for C-type lectin receptor binding and that these interactions occur in a Ca^2+^-dependent fashion.

## Introduction

Many pathogens display blood group antigens and their molecular mimics on their cell surfaces to escape the immune system of the host^1–3^. For example, *Escherichia coli* O86:K2:H2 and O86:K61:B7 display B-antigen oligosaccharides core (α-D-Gal-(1-3)-[α-L-Fuc-(1-2)]β-D-Gal-(1-3)-α-D-GalNac-(1-3)-GalNAc) in the lipopolysaccharide part and cause diarrhea in children^4^. Similarly, *E*. *coli* O90, O127 and O128 also express human blood group antigen epitopes and Lewis^x^ on their cell surfaces to evade host immune responses^5^. In 1960s, Springer and co-workers reported that several gram-negative bacteria display blood group determinants^6^. Moreover, cancer-associated carbohydrate antigens such as Globo-H carry structural features of O-antigen^7,8^. ABO(H) antigens are composed of distinct carbohydrate structures that differ only in their terminal monosaccharide structures. Animal lectins such as siglecs, galectins and C-type lectins have been shown to mediate pathogen recognition and subsequent immune responses^9–14^. In addition, galectins binding preference to blood group antigens was described^15,16^. Given the importance of C-type lectin receptors as microbial and cancer-associated pattern recognition receptors, herein, we report the synthesis of ABO active tri- and disaccharides that were then printed as glycan microarrays to study C-type lectin receptor (CLR) binding specificities. CLR are defined as such by their Ca^2+^-dependent binding to their antigens of selective carbohydrate-protein interactions^17^. Mg^2+^ are also known to have stabilizing effect albeit weaker than the Ca^2+^ in CLR binding^18^. Using four CLRs as CLR-Fc fusion proteins, we investigate glycan-protein interactions in a carbohydrate array format. The selected CLR-hFc fusion proteins were human DC-SIGN and three murine CLRs (Mincle-hFc, MGL-1-hFc and SIGNR3-hFc). Based on the binding preferences, we have rationalized the C-type lectin selectivity.

## Results and Discussion

### Synthesis of ABO active glycans

**Figure 1:**
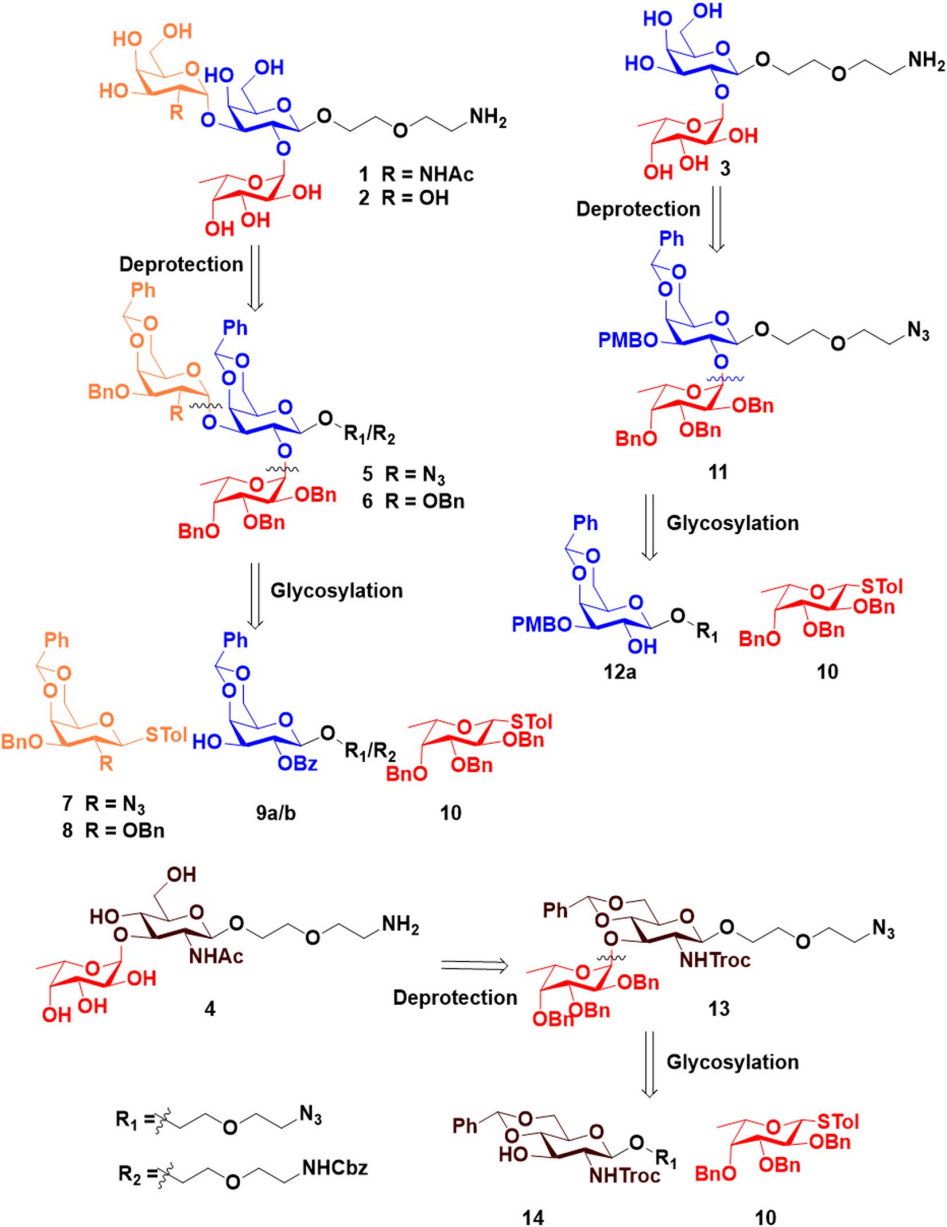
Retrosynthesis of compounds **1-4**.

**Figure 2:**
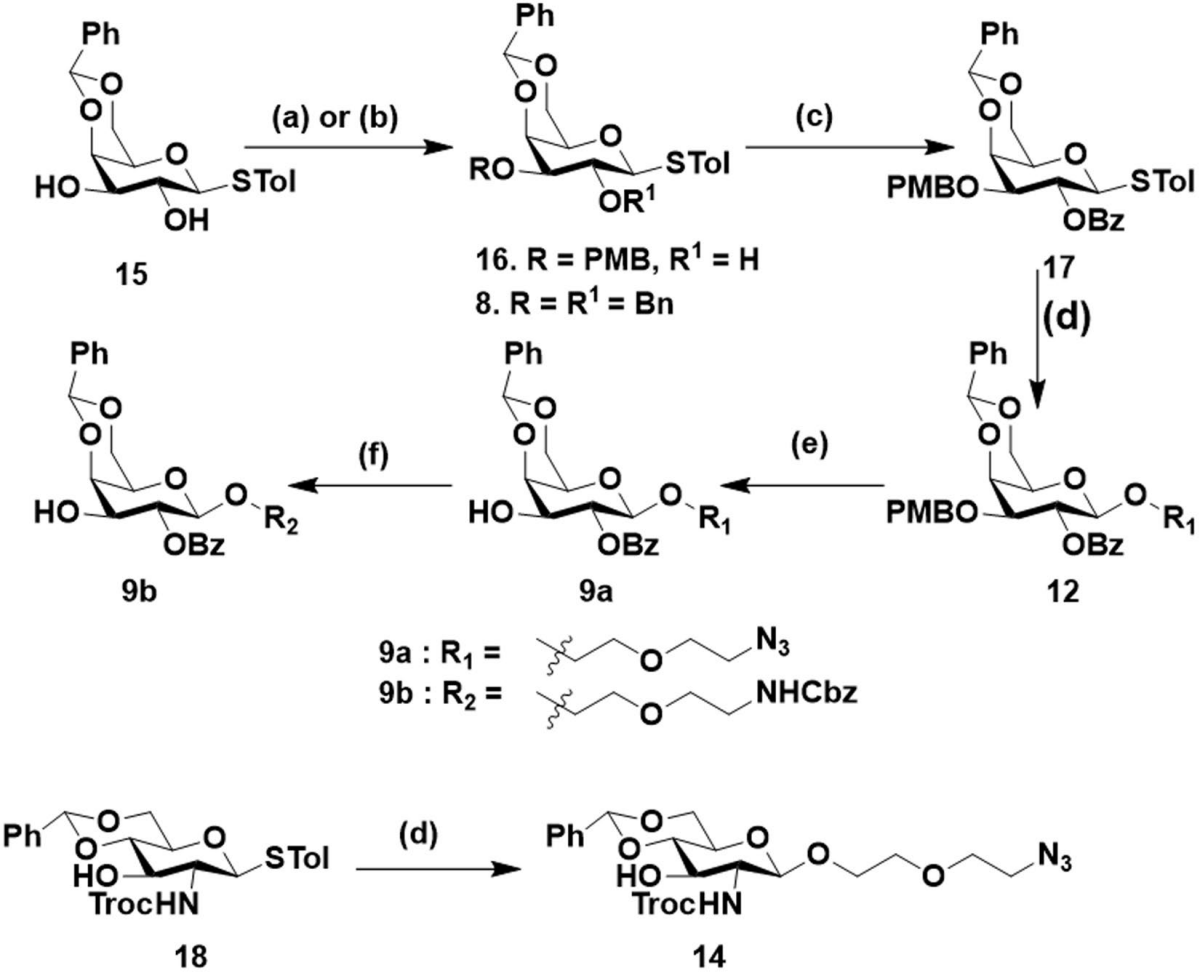
Synthesis of building blocks: (a) PMBCl, dibutyltinoxide, Bu_2_SnO, TBAI, toluene, 104 °C; (b) BnBr, NaH, DMF, 0 °C; (c) BzCl/Pyridine, 0 °C; (d) 2-azidoethoxyethanol, NIS/TfOH, DCM, −40 °C, 4 Å molecular sieves (e) DDQ, DCM/Water [18:1 (v/v)], (f) Zn, AcOH, and CbzCl, NaHCO_3,_ THF/H_2_O, 0 °C.

**Figure 3:**
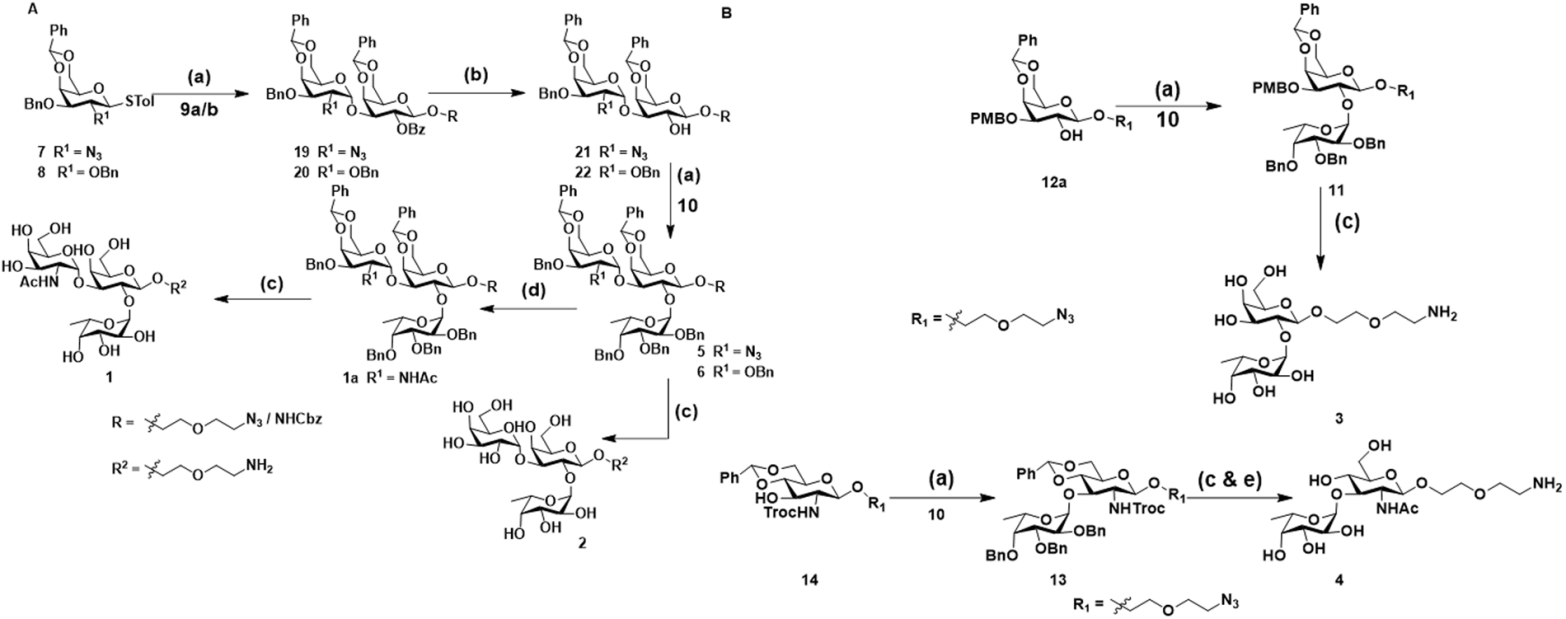
**(A** and **B**). Synthesis of **1-4** glycans: (a) NIS/TfOH, DCM, −40 to −20 °C, 4 Å molecular sieves **7**/**8** with **9a**/**b**; **12a/14/21/22** with **10**; (b) NaOMe, MeOH; (c) HCOOH, Pd(OH)_2_/H_2_, MeOH; (d) Zn, THF, AcOH, Ac_2_O(v/v, 3:2:1)**;** (e) LiOH.H_2_O, 1,4*-d*ioxane:H_2_O (v/v, 1:1), 80 °C.

The ABO active glycan and fucosyl derivative (**1-4**) were synthesized from fully protected tri and disaccharides (**5**, **6**, **11** and **13**), respectively **(**Fig. 1). The assembly of A and B-antigens (GalNAcα(1-3)(Fucα(1-2))GalβPEGNH_2_ **(1**) and Galα(1-3)(Fucα(1-2))GalβPEGNH_2_) **(2**) involved the thiogalactoside building block **7** and **8**, and of reducing end sugar **9a/b**. In contrast, the assembly of **3** (O-antigen, Fucα(1-2)GalβPEGNH_2_) and **4** (Fucα(1-3)GlcNAcβPEGNH_2_) involved the assembly of thiofucoside donor **10** and reducing end sugar 4,6-benzilidine-galactoside **12a** and glucosamine acceptor **14**, respectively. The thiogalactoside building block **7** and thiofucoside donor **10** were synthesized as previously described^19–22^. The donor **8** was synthesized from a known thiogalactoside donor **15**, which was synthesized from D-galactose in 4 steps, as described^22^. Benzylation of the C-2 and C-3 hydroxyl function of **15** provided the desired thiogalactoside building block **8 (**Fig. 2). The synthesis of thiogalactose acceptor **9a/b** involved the C-3 PMB protection of **15** by using PMBCl in the presence of stannylene acetate formation, followed by C-2 benzoylation and glycosylation with 2-azidoethoxyethanol linker yielded **12**, and the cleavage of C-3 PMB ether protection in the presence of 2,3-dichloro-5,6,-dicyano-1,4-benzoquinone (DDQ) yielded acceptor **9a** and *in situ* protection of Cbz by reduction of linker azide yielded **9b (**Fig. 2). The 4,6-benzilidine-NHTroc-glucosamine reducing end sugar was synthesized from **18**^23^ by glycosylation with the linker to yield **14**. With all building blocks in hand, we carried out glycosylation using standard NIS/TfOH mediated condition at −40 °C. The thiogalactoside donor **8** and 2-azido-2-deoxy-thiogalacoside donor **7** was glycosylated with **9a/b** to obtain disaccharides **19** and **20 (**Fig. 3A). To control the stereochemistry of GalN3-α(1-3)-Gal **(19**) and Gal-α(1-3)-Gal **(20**), the fucosyl building block **10** was incorporated after the assembly of the disaccharides. Finally, the global deprotection and acetylation of galactosamine resulted in the fully deprotected A- and B-group trisaccharides **(1-2**) in moderate yields **(**Fig. 3A). Similarly, glycosylation between thiofucoside donor **10** with acceptor **12a** and **14** yielded fully protected disaccharides **11** and **13**, respectively. Finally, the global deprotection, followed by acetylation of glucosamine resulted in the disaccharides **3** and **4** in reasonable yields **(**Fig. 3B).

### Microarray analysis

**Figure 4:**
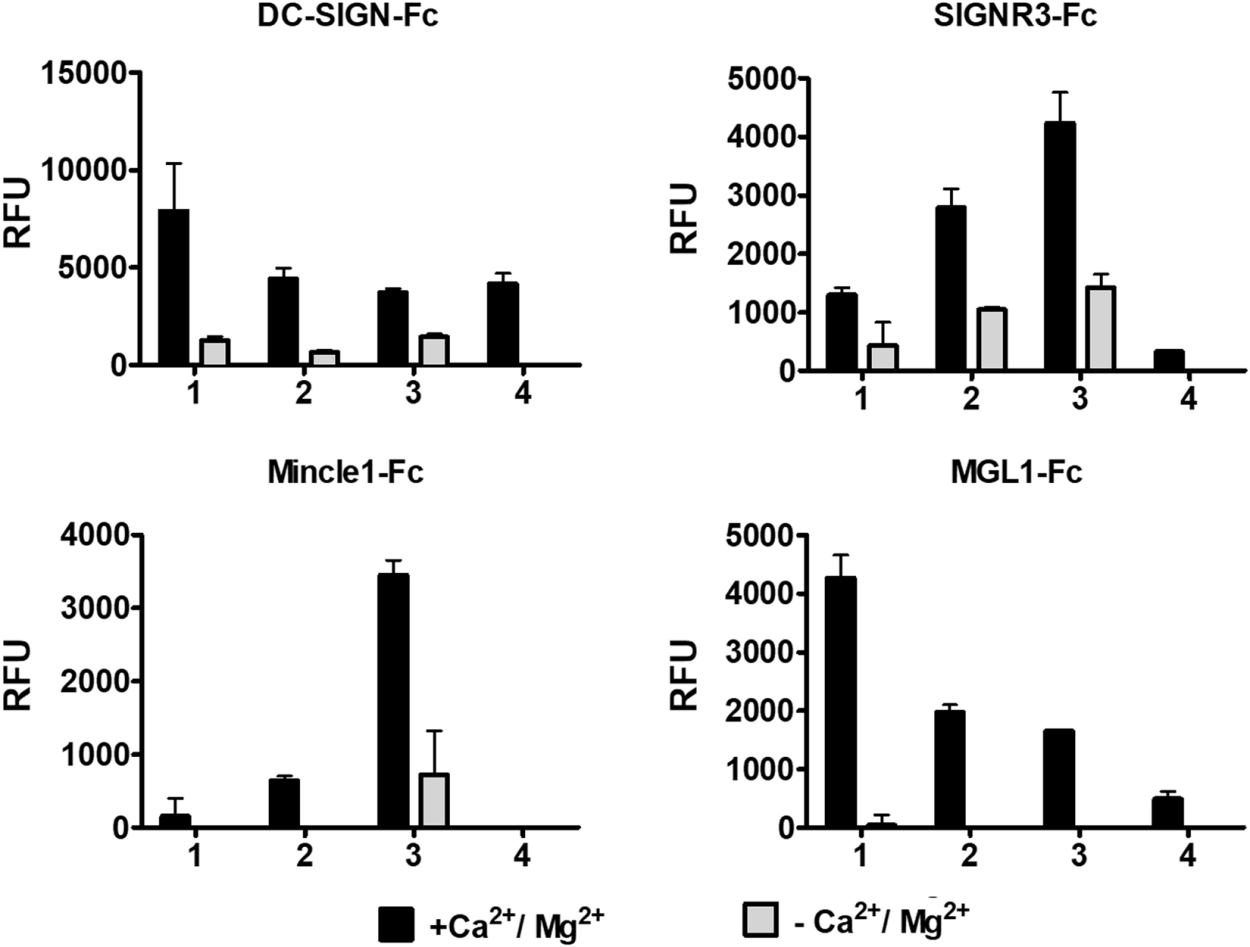
Microarray analysis of compounds 1-4 with C-type lectin-Fc fusion proteins (DC-SIGN, SIGNR3, Mincle and MGL1). Mean ± SD of four replicates per measurement.

Next, the synthetic **1-4** were printed onto epoxide-functionalized microarray slides at 50 μM in replicates of four, as described in the experimental section^24,25^. CLR-hFc fusion proteins were incubated on the slide at 20 ng μl^−1^ (DC-SIGN, SIGNR3, Mincle and MGL-1)^26,27^ in optimized conditions of 50 mM HEPES buffer with 5 mM CaCl_2_, 5 mM MgCl_2_, 0.005% Tween-20 and 1% ovalbumin, followed by the secondary antibody (Cy3-anti-human IgG). During all binding and washing steps divalent cations were maintained. In control blocks, divalent cations were omitted from all binding and washing steps. Slides were scanned and the binding was determined by the fluorescence intensity, as described in the experimental section **(**Fig. 4). In addition, binding to **1-4** on the microarray was analyzed using IVIG (at 1 µg μl^−1^; human IgG pool, known to bind many glycans) and the biotinylated plant lectin PNA at 20 ng μl^−1^. As expected, the PNA lectin, a galactose specific plant lectin, bound to **2-3**, and the human IgG pool bound to ABO antigens confirming the conjugation of glycans to the slides (Fig. S6). Furthermore, all four C-type lectins displayed binding preference to ABO active saccharides. However, they exhibited different binding preferences, clearly indicating the differences in structure-binding relationships. Human DC-SIGN, a fucose-specific lectin^28–32^ bound to all four glycans with statistically significant greater responsiveness towards **1**, suggesting that galactose residue may also be important for optimal binding preferences. In contrast, the high-mannose and fucose glycan specific SIGNR3 lectin^33–36^ showed weak binding of **4** compared to **1-3**, highlighting that the structural-activity relationship of SIGNR3 is different from DC-SIGN. On the other hand, MGL-1^37^ showed preferential binding towards A-antigen active trisaccharide compared to compound **2-4** which were also recognized^38–40^. Finally, Mincle lectin, which is known to bind trehalose derivatives^41^, surprisingly displayed some binding preference towards **3** compared to the other glycans. The disparity in the binding preference clearly indicates the galactose/galactosamine and fucose residues of **1-3** significantly influence the binding pattern of the CLR-Fc fusion proteins. In order to analyze the Ca^2+^ dependency of the CLR-hFc interactions with **1-4**, we screened the binding preferences in the absence of Ca^2+^ (and Mg^2+^) ions. As expected, the binding affinity of specific antigen active molecules to the CLR-hFc fusion proteins significantly decreased. Some of these lectins had been previously examined on glycan microarrays of the Consortium for Functional Glycomics (CFG; http://www.functionalglycomics.org/static/consortium/resources/resourcecoreh.shtml), including against blood group antigens. DC-SIGN and MGL1 exhibited somewhat different binding patterns, while SIGNR3 had not been examined. Binding patterns may be affected by ABO semi-synthetic oligosaccharides vs full length oligosaccharides microarray analysis, differences in the glycan linker, conjugation chemistry to the slides, microarray slide coat, and lectin binding detection method^10a^. Altogether, the structure-activity data shows that blood group antigens bind to C-type lectins, yet with different preferences. Future studies could address binding affinities and avidity while examining multimeric CLR units to resemble the native receptors function. Furthermore, these results provide the basis for understanding of how pathogens may target CLRs by molecular mimicry to evade immune responses.

## Conclusions

In conclusion, we have synthesized a series of blood group antigens and immobilized them on glycan microarray slides to determine C-type lectin receptor binding preferences. We have demonstrated that galactose and fucose moieties in **1-4** significantly influence the binding preferences of specific C-type lectins, in a Ca^2+^-dependent manner. Overall, comparison of the C-type lectin binding patterns allows for further understanding of the basic differences in their preferential recognition of blood group antigens that constitutes a valuable tool for interfering with these interactions.

## Methods

### General procedure for the production of the CLR-Fc library

The general procedure for the production of human and mouse CLR-Fc fusion protein library has been described previously^24,42^. The following primers **(**Table 1) were used for PCR amplification of cDNA fragments encoding the extracellular part of the respective C-type lectin receptor (CLR).

**Table 1:** Primers for production of human and mouse CLR-Fc fusion protein library.

DC-SIGN	DC-SIGN-fw	5′-GAATTCGTCCAAGGTCCCCAGCTCCAT-3′
SIGNR3	DC-SIGN-rev	5′-CCATGGACGCAGGAGGGGGGTTTGGGGT-3′
	SIGNR3-fw	5′-GAATTCCATGCAACTGAAGGCTGAAG-3′
	SIGNR3-rev	5′-AGATCTTTTGGTGGTGCATGATGAGG-3′
Mincle	Mincle-fw	5′-CCATGGGGCAGAACTTACAGCCACAT-3′
	Mincle-rev	5′-AGATCTGTCCAGAGGACTTATTTCTG-3′
MGL-1	MGL-1-fw	5′-CCAGTTAAGGAGGGACCTAGGCAC-3′
	MGL-1-rev	5′-AGCTCTCCTTGGCCAGCTTCATC-3′

The cDNA fragments were cloned into the pDrive cloning vector (Qiagen) and further ligated into the pFuse-hIgG1-Fc expression vector (InvivoGen). Next, the CLR-hFc encoding vectors were transiently transfected using the FreeStyle Max CHO-S Expression System (Life Technologies). The cell supernatant containing the CLR-hFc fusion proteins was collected and the CLR-hFc fusion proteins were purified using HiTrap Protein G HP columns (GE Healthcare). Identity and purity of the CLR-Fc fusion proteins were confirmed by SDS-PAGE with subsequent Coomassie stain as well as Western Blot. Concentration determination was performed using the Micro BCA Protein Assay Kit (Thermo Scientific).

### Microarray fabrication

Arrays were fabricated with NanoPrint LM-60 Microarray Printer (Arrayit) on epoxide-derivatized slides (PolyAn 2D) with 16 sub-array blocks on each slide. Glycoconjugates were distributed into 384-well source plates using 4 replicate wells per sample and 8 µl per well (Version Vr.). Each glycoconjugate was prepared at 50 µM in an optimized print buffer (300 mM phosphate buffer, pH 8.4 supplemented with 0.005% Tween-20). To monitor printing quality AlexaFlour-555-Hydraside (Invitrogen, at 2 ng/µl in 178 mM phosphate buffer, pH 5.5) was used for each printing run. The arrays were printed with four SMP3 pins (5 µm tip, 0.25 µl sample channel, ~100 µm spot diameter; Arrayit), with spot to spot spacing of 275 µm. The humidity level in the arraying chamber was maintained at about 70% during printing. Printed slides were left on arrayer deck over-night, allowing humidity to drop to ambient levels (40–45%). Next, slides were packed, vacuum-sealed and stored at room temperature (RT) until used.

### Microarray binding assay

Slides were developed and analyzed as previously described^24,25^ with some modifications. Slides were rehydrated with dH_2_O and incubated for 30 min in a staining dish with 50 °C pre-warmed ethanolamine (0.05 M) in Tris-HCl (0.1 M, pH 9.0) to block the remaining reactive epoxy groups on the slide surface, then washed with 50 °C pre-warmed dH_2_O. Slides were centrifuged at 200 × *g* for three min then fitted with ProPlate™ Multi-Array 16-well slide module (Grace Bio-lab P37001) to divide into the sub-arrays (blocks). Slides were washed with washing buffer (50 mM HEPES pH 7, 5 mM CaCl_2_, 5 mM MgCl_2_, 0.005% Tween 20), aspirated and blocked with 200 µl/sub-array of Blocking buffer (50 Mm HEPES pH 7, 5 mM CaCl_2_, 5 mM MgCl_2_, 0.005% Tween 20 and 1% w/v Ovalbumin) for 1 hour at RT with gentle shaking. Next, the blocking buffer was aspirated and 100 µl/block of C-type lectins (20 ng/µl), plant lectin Bio-PNA (20 ng/µl) and IVIG GammaGard 1 mg/ml (all diluted blocking buffer, except Bio-PNA that was diluted in blocking buffer C: 50 Mm HEPES pH 7, 20 mM CaCl_2_, 5 mM MnCl_2_, 0.005% Tween 20 and 1% w/v Ovalbumin) were incubated with gentle shaking for 2 hours at RT. Slides were washed 4 times with washing buffer, then with washing buffer without Tween-20 for 2 min. Bound antibodies were detected by incubating with secondary detection diluted in washing buffer, 200 µl/block at RT for 1 hour: Cy3-anti-human IgG, H + L (1.5 µg/ml) or Cy3-SA (0.75 µg/ml). Slides were washed 4 times with washing buffer, then with washing buffer for 10 min followed by removal from ProPlate™ Multi-Array slide module and immediately dipping in a staining dish with dH_2_O supplemented with 5 mM CaCl_2_, 5 mM MgCl_2_ for 10 min with shaking. Slides then were centrifuged at 200 × *g* for 3 min and the dry slides immediately scanned.

### Array slide processing

Processed slides were scanned and analyzed described^4,5^ at 10 μm resolution with a Genepix 4000B microarray scanner (Molecular Devices) using 350 gain. Image analysis was carried out with Genepix Pro 6.0 analysis software (Molecular Devices). Spots were defined as circular features with a variable radius as determined by the Genepix scanning software. Local background subtraction was performed.

## Electronic supplementary material

Supplementary Information
