# Supplementary material for: ABO Antigens Active Tri- and Disaccharides Microarray to Evaluate C-type Lectin Receptor Binding Preferences
Source: Sci Rep. 2018 Apr 26;8:6603. doi: 10.1038/s41598-018-24333-y (PMC5920051; doi:10.1038/s41598-018-24333-y)
Supplement: Supplementary file 1 — Supplementary Information [file 41598_2018_24333_MOESM1_ESM.pdf]

ABO Antigens Active Tri- and Disaccharides Microarray to Evaluate C-type Lectin Receptor Binding Preferences

Chethan D. Shanthamurthy,[a] Prashant Jain,[a] Sharon Yehuda,[b] Joao Monteiro,[c] Shani Leviatan Ben-Arye,[b] Balamurugan Subramani,[a] Bernd Lepenies,*[c] Vered Padler-karavani*[b] and Raghavendra Kikkeri *[a]

[a]Indian Institute of Science Education and Research, Dr. Homi Bhabha Road, Pune-411008, India. Email: rkikkeri@iiserpune.ac.in

[b] Tel-Aviv University, Department of Cell Research and Immunology, Tel-Aviv 69978, Israel, E-mail: vkaravani@post.tau.ac.il

[c]University of Veterinary Medical Hannover, Immunology Unit & Research Center for Emerging Infection and Zoonoses, Hannover, Germany. E-mail: bernd.lepenies@tiho-hannover.de.

Table of Contents:

1. **General Information**
2. **Synthesis of compound 1-4.**

1. General Information

All chemicals were reagent grade and used as supplied except where noted. Analytical thin layer chromatography (TLC) was performed on Merck silica gel 60 F254 plates (0.25 mm). Compounds were visualized by UV irradiation or dipping the plate in CAM solution followed by heating. Column chromatography was carried out using force flow of the indicated solvent on Fluka Kieselgel 60 (100–200 mesh). ^{1}H and ^{13}C NMR spectra were recorded on Jeol 400 MHz using residual solvents signals as an internal reference (Chloroform-*d* δH, 7.26 ppm, δc 77.3 ppm and Methanol-*d*4 δH 3.31 ppm, δc 49.0 ppm). The chemical shifts (δ) are reported in *ppm* and coupling constants (*J*) in Hz. PBSx10 was purchased from Hy-labs, ethanolamine from Fisher, ovalbumin (Grade V), sodium phosphate monobasic monohydrate, sodium phosphate dibasic heptahydrate, Tween-20 and Tris/HCl were purchased from Sigma-Aldrich. Antibodies mouse IgG ChromoPure whole molecule, Cy3-anti-human IgG H+L, Cy3-sterptavidin (Jackson ImmunoResearch). Mouse IgM Kappa mouse myeloma TEPC 183 (ICL). plant lectins Bio-PNA, (Vector Labs). IVIG GammaGard 10% (Baxter, USA) were a kind gift from Dr. Adriana Tremolot, Childrens hospital UCSD.

2. Synthesis of compound 1-4.

Synthesis of building blocks (9a/b, 12 and 14).

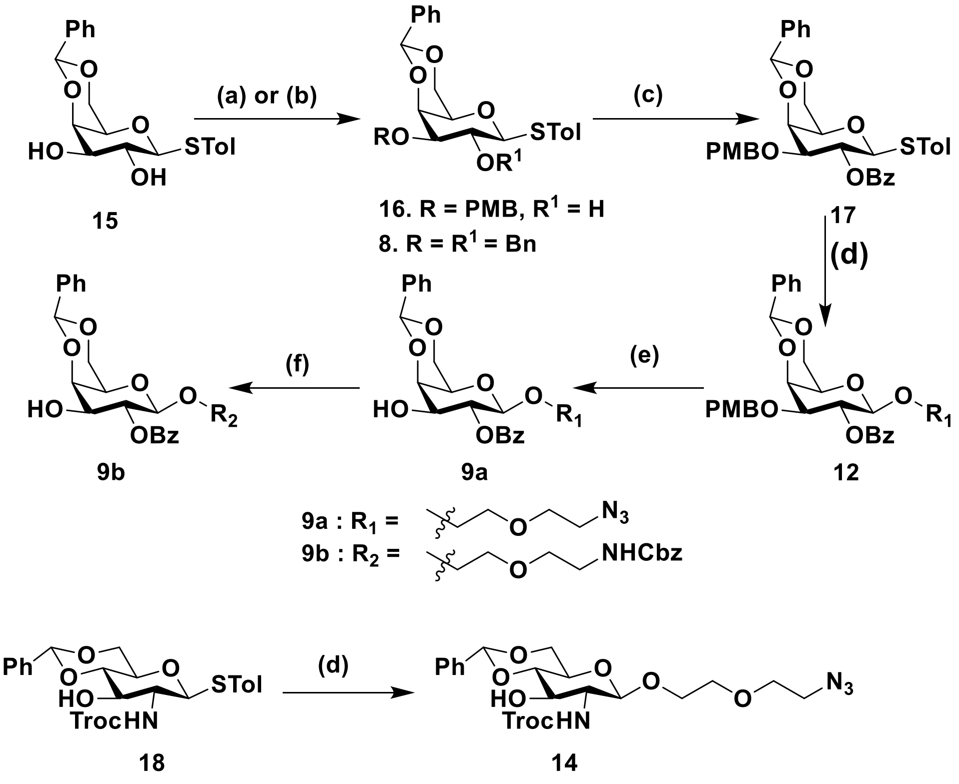

Figure S1. Synthesis of galactose building blocks (a) PMBCl, dibutyltinoxide, Bu_2SnO, TBAI, *p*-toluene, 104 ˚C; (b) BnBr, NaH, DMF, 0 ˚C; (c) BzCl/Pyridine, 0 ˚C; (d) 2-azidoethoxyethanol, NIS/TfOH, DCM , -40 ˚C, 4 Å molecular sieves (e) DDQ, DCM/Water [18:1 (v/v)]. (f) Zn, AcOH, and CbzCl, $NaHCO_3$THF/H_2O.

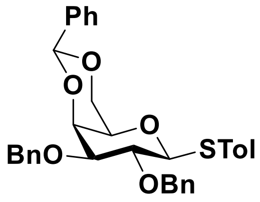

8

Compound **15** (1.2 g, 3.20 mmol) was dissolved in dry DMF (8 mL) and 60% of NaH (0.38 g, 16.04 mmol) at r.t benzyl bromide (1.53 mL, 12.83 mmol) was then added drop wise under anhydrous condition at 0 °C. After stirring overnight the reaction mixture was quenched by methanol, and extracted with EtOAc and washed water. The combined organic layer washed with brine solution, dried over Na_2SO_4 and concentrated under reduced pressure. The residue was purified by column chromatography (EtOAc/Hexane = 1/4) to afford **8** (1.2 g, 70%) as white solid. ^{1}H NMR (400 MHz, Chloroform-*d*) δ 7.61 (d, *J* = 8.1 Hz, 2H), 7.53 (dd, *J* = 6.7, 3.0 Hz, 2H), 7.44 (d, *J* = 8.5 Hz, 2H), 7.41 – 7.38 (m, 4H), 7.36 – 7.32 (m, 4H), 7.31 – 7.28 (m, 3H), 7.01 (d, *J* = 8.2 Hz, 2H), 5.48 (s, 1H), 4.75 – 4.67 (m, 4H), 4.58 (d, *J* = 9.5 Hz, 1H), 4.37 (dd, *J* = 12.3, 1.3 Hz, 1H), 4.15 (d, *J* = 3.3 Hz, 1H), 3.98 (dd, *J* = 12.3, 1.4 Hz, 1H), 3.84 (t, *J* = 9.3 Hz, 1H), 3.63 (dd, *J* = 9.2, 3.4 Hz, 1H), 3.41 (s, 1H), 2.31 (s, 3H). ^{13}C NMR (100 MHz, Chloroform-*d*) δ 138.72, 138.26, 138.06, 137.81, 133.61, 129.78, 129.18, 128.79, 128.54, 128.48, 128.33, 128.26, 127.97, 127.83, 126.82, 101.49, 86.77, 81.59, 75.56, 75.49, 73.81, 71.98, 69.91, 69.61, 21.30.

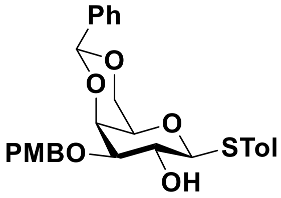

16

A solution of compound **15** (2.5 g, 6.68 mmol) in dry toluene (30 mL) was placed on a dean-stark apparatus set up and then dibutyltinoxide (1.83 g, 7.35 mmol) was added. The reaction mixture was refluxed at 110 °C for 12 h. The mixture was cooled to r.t and were added *p*-methoxybenzylchloride (1.36 mL, 10.03 mmol) and TBAI (0.49 g, 1.34 mmol) and refluxed the reaction mixture for 12 h. The mixture was diluted with EtOAc and washed with water, dried over Na_2SO_4 and concentrated under reduced pressure. The residue was purified by column chromatography (EtOAc/Hexane = 1/4) to afford **16** (2.1 g, 65%) as white solid. ^{1}H NMR (400 MHz, Chloroform-*d*) δ 7.57 (d, *J* = 8.1 Hz, 2H), 7.42 – 7.32 (m, 5H), 7.28 – 7.256 (m, 2H), 7.05 (d, *J* = 8.0 Hz, 2H), 6.84 (d, *J* = 8.6 Hz, 2H), 5.41 (s, 1H), 4.65 (dd, *J* = 11.9,11.9 Hz 2H), 4.46 (d, *J* = 9.4 Hz, 1H), 4.35 (dd, *J* = 12.3, 1.3 Hz, 1H), 4.11 (d, *J* = 3.2 Hz, 1H), 3.97 (dd, *J* = 12.3, 1.5 Hz, 1H), 3.84 (dd, *J* = 18.1, 8.8 Hz, 2H), 3.79 (s, 3H), 3.48 (dd, *J* = 9.3,3.3 Hz 1H), 3.43 (s, 1H), 2.33 (s, 3H). ^{13}C NMR (100 MHz, Chloroform-*d*) δ 159.52, 138.51, 138.00, 130.1 , 129.16, 128.20, 126.75, 126.69, 114.00, 101.31, 87.21, 79.94, 73.45, 71.40, 70.18, 69.56,67.21, 55.41, 21.37.

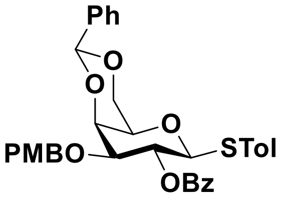

17

Compound **16** (2.1 g, 4.25mmol) was dissolved in a mixture of dry CH_2Cl_2 (10 mL) and pyridine (15 mL). Benzoyl chloride (0.59 mL, 5.10 mmol) was then added drop wise at 0 °C. After stirring overnight the reaction mixture was quenched by 2 mL of isopropanol, solvents were evaporated under reduced pressure and the residue was extracted with EtOAc and 2 N HCl. The combined organic layer washed with brine solution, dried over Na_2SO_4 and concentrated under reduced pressure. The residue was purified by column chromatography (EtOAc/Hexane = 1/2) to

afford **17** (2.2 g, 88%) as a white solid. ^{1}H NMR (400 MHz, Chloroform-*d*) δ 8.04 (dd, *J* = 8.3, 1.3 Hz, 2H), 7.60 (tt, *J* = 6.9, 1.3 Hz, 1H), 7.48 (m, 4H), 7.44 (dd, *J* = 6.8, 2.8 Hz, 2H), 7.40 – 7.35 (m, 3H), 7.10 – 7.04 (m, 4H), 6.68 – 6.64 (m, 2H), 5.50 – 5.46 (m, 2H), 4.75 (d, *J* = 9.8 Hz, 1H), 4.52 (dd, *J* = 12.4, 12.4 Hz, 2H), 4.38 (dd, *J* = 12.3, 1.5 Hz, 1H), 4.21 (d, *J* = 2.9 Hz, 1H), 4.02 (dd, *J* = 12.3, 1.6 Hz, 1H), 3.75 (d, *J* = 3.4 Hz, 1H), 3.73 (s, 3H), 3.48 (s, 1H), 2.32 (s, 3H). ^{13}C NMR (100 MHz, Chloroform-*d*) δ 165.36, 159.65, 138.57, 138.17, 134.79, 133.36, 130.80, 130.29, 130.22, 129.90, 129.72, 129.42, 128.74, 128.49, 128.09, 127.12, 114.11, 101.72, 85.98, 78.29, 73.71, 71.12, 70.53, 69.77, 69.67, 55.61, 21.66.

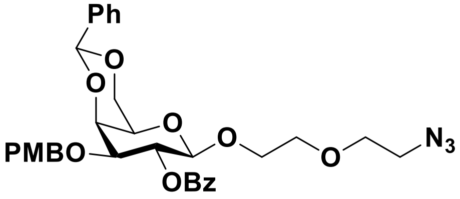

12

A solution of **17** (2.2 g, 3.67 mmol) and 2-azidoethoxyethanol (0.53 g, 4.04 mmol) and and activated 4Å powdered molecular sieves (1.5 g) in anhydrous dichloromethane (5 mL) was stirred for 2 h at room temperature under an argon atmosphere, and then cooled to -40^{0}C followed by addition of NIS (1.24 g, 5.51 mmol), TfOH (0.064 mL, 0.73 mmol). The reaction mixture was stirred at -40 ^{0}C for 0.5 h to 3 h until the disappearance of the donor on TLC, then quenched with triethylamine (81 μL, 0.81 mmol, 0.75 equiv) and warmed to room temperature. The mixture was diluted with dichloromethane, filtered through Celite, washed with 20% aqueous $Na_2S_2O_3$ solution, dried over Na_2SO_4, and concentrated under reduced pressure. The residue was purified by column chromatography (EtOAc/Hexane = 1/3) to afford **12** (1.8 g, 81%) as white solid. ^{1}H NMR (400 MHz, Chloroform-*d*) δ 8.04 (dd, *J* = 8.3, 1.2 Hz, 2H), 7.621 – 7.56 (m, 3H), 7.46 (t, *J* = 7.7 Hz, 2H), 7.37 (q, *J* = 5.6 Hz, 3H), 7.15 (d, *J* = 8.6 Hz, 2H), 6.72 (d, *J* = 8.6 Hz, 2H), 5.61 (dd, *J* = 10.1, 8.0 Hz, 1H), 5.53 (s, 1H), 4.67 (d, *J* = 8.0 Hz, 1H), 4.58 (dd, *J* = 12.5, 12.5 Hz, 2H), 4.34 (d, *J* = 12.3 Hz, 1H), 4.21 (d, *J* = 3.3 Hz, 1H), 4.07 (dd, *J* = 12.3, 1.6 Hz, 1H), 4.00 (dt, *J* = 11.2, 3.8 Hz, 1H), 3.75 (s, 3H), 3.72 (td, *J* = 6.5, 2.6 Hz, 2H), 3.58 (tp, *J* = 11.3, 4.3, 3.9 Hz, 2H), 3.46 – 3.42 (m, 3H), 3.09 – 2.95 (m, 2H). ^{13}C NMR (100 MHz, Chloroform-*d*) δ 165.29, 159.40, 137.83, 133.08, 130.45, 130.04, 129.92, 129.43, 129.09,

128.43, 128.27, 126.61, 113.87, 101.50, 101.37, 76.69, 73.44, 70.89, 70.62, 70.10, 69.29, 68.73, 66.90, 55.35, 50.76.

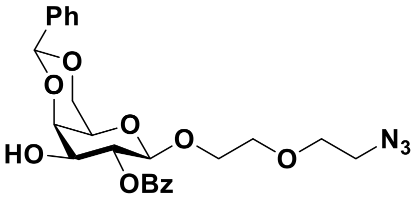

9a

Compound **12** (2.2 g, 3.63 mmol) was dissolved in mixture of CH_2Cl_2 and water (15 mL, 18/1, v/v). 2,3-*d*ichloro-5,6-*d*icyano-1,4-benzoquinone (DDQ, 2.47 g, 10.91 mmol) was added in three equal portions over a interval of half an hour at room temperature. After stirring overnight the reaction mixture was extracted with CH_2Cl_2 and water. The combined organic layer washed with brine solution, dried over Na_2SO_4 and concentrated under reduced pressure. The residue was purified by column chromatography (EtOAc/Hexane = 1/1) to afford **9a** (1.7 g, 96%) . ^{1}H NMR (400 MHz, Chloroform-*d*) δ 8.08 (dd, *J* = 8.4, 1.3 Hz, 2H), 7.60 – 7.52 (m, 3H), 7.47 – 7.43 (m, 2H), 7.41 – 7.39 (m, 3H), 5.59 (s, 1H), 5.37 (dd, *J* = 9.9, 8.0 Hz, 1H), 4.73 (d, *J* = 8.0 Hz, 1H), 4.38 (dd, *J* = 12.4, 1.3 Hz, 1H), 4.28 (d, *J* = 3.7 Hz, 1H), 4.12 (dd, *J* = 12.5, 1.7 Hz, 1H), 4.07 – 3.0 (m, 1H), 3.93 (td, *J* = 9.7, 3.3 Hz, 1H), 3.78 (ddd, *J* = 11.2, 7.3, 3.6 Hz, 1H), 3.61 (dt, *J* = 7.2, 3.7 Hz, 1H), 3.57 – 3.56 (m, 1H), 3.53 – 3.46 (m,21H), 2.63 (d, *J* = 10.7 Hz, 1H). ^{13}C NMR (100 MHz, Chloroform-*d*) δ 166.26, 137.43, 133.26, 130.04, 129.90, 129.39, 128.44, 128.37, 126.51, 101.56, 101.09, 75.71, 72.92, 71.70, 70.61, 70.14, 69.08, 69.03, 66.69, 50.68. HRMS (ESI) m/z: calc'd for $C_{24}H_{27}N_3O_8Na$: 508.1696, found: 508.1694.

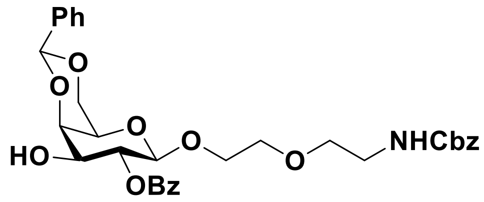

9b

Compound **9a** (0.73 g, 1.5 mmol) was dissolved in acetic acid (10 mL) and added zinc dust (0.7g, 10.70mmol). After 4 h filter off zinc on celite bed and evaporated AcOH then co-evaporated with CH_2Cl_2 (3x). The crude product was dissolved in THF:H_2O (v/v, 2:1, 15 mL) and were added $NaHCO_3$ and CbzCl (0.32 mL, 2.25 mmol). The mixture was stirred at room temperature for 6 h and extracted with EtOAc and washed with water. The organic layers were combined, washed with brine, dried over Na_2SO_4 and concentrated under reduced pressure. The crude was purified by column chromatography (EtOAc/Hexane = 1/1) to yield compound **9b** (0.51 g, 57%). ^{1}H NMR (400 MHz, Chloroform-*d*) δ 8.05 (dd, *J* = 8.3, 1.1 Hz, 2H), 7.55 – 5.52 (m, 3H), 7.43 – 7.31 (m, 10H), 5.57 (s, 1H), 5.37 (dd, *J* = 9.8, 8.1 Hz, 1H), 5.10 – 5.04 (m, 2H), 4.68 (d, *J* = 8.0 Hz, 1H), 4.36 (d, *J* = 12.4 Hz, 1H), 4.23 (d, *J* = 3.2 Hz, 1H), 4.07 (d, *J* = 12.4 Hz, 1H), 3.98 (dt, *J* = 11.0, 4.0 Hz, 1H), 3.9 – 3.89 (m, 1H), 3.77 – 3.72 (m, 1H), 3.56 (t, *J* = 4.3 Hz, 2H), 3.52 (s, 1H), 3.39 (t, *J* = 5.0 Hz, 2H), 3.17 – 3.14 (m, 2H), 2.71 – 2.70 (m, 1H). ^{13}C NMR (100 MHz, Chloroform-*d*) δ 166.36, 156.48, 137.43, 136.77, 133.35, 130.00, 129.90, 129.48, 128.64, 128.50, 128.44, 128.29, 128.21, 126.55, 101.68, 100.97, 75.76, 72.80, 71.80, 70.27, 70.17, 69.11, 68.44, 66.81, 66.75, 40.93, 29.67.

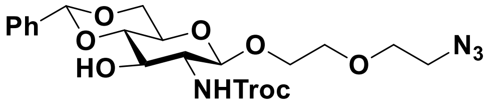

14

Compound **18** (1.8 g, 3.28 mmol) and 2-azidoethoxyethanol (0.34 g, 2.62 mmol) were dissolved in dry CH_2Cl_2 (18 mL), and freshly dried 4 Å MS, were added under anhydrous conditions. After stirring for 1 h at r.t, the reaction flask was cooled at -40 °C and NIS (1.10g, 4.92mmol), TfOH (0.058ml, 0.66mmol) were added, the reaction was monitored by TLC. After the completion of reaction, quenched with Et_3N and diluted with CH_2Cl_2. Molecular sieves were filtered off on

celite bed and the organic layer washed with $Na_2S_2O_3$, dried over Na_2SO_4, filtered and concentrated under reduced pressure. The residue was purified by column chromatography (EtOAc/Hexane = 1/3) to afford **14** (1.09 g, 60%). ^{1}H NMR (400 MHz, Chloroform-*d*): δ 7.55 – 7.49 (m, 2H), 7.42 – 7.37 (m, 3H), 5.91 (s, 1H), 5.55 (s, 1H), 4.83 (d, *J* = 12.1 Hz, 1H), 4.73 – 4.67 (m, 2H), 4.35 (dd, *J* = 10.5, 5.0 Hz, 1H), 3.97 (dt, *J* = 11.7, 3.4 Hz, 2H), 3.84 – 3.76 (m, 2H), 3.75 – 3.68 (m, 2H), 3.67 – 3.61 (m, 2H), 3.58 (t, *J* = 9.2 Hz, 2H), 3.54 – 3.43 (m, 4H). ^{13}C NMR (100 MHz, Chloroform-*d*) δ 155.43, 137.09, 129.40, 128.45, 126.46, 101.95, 95.55, 81.34, 74.82, 71.89, 71.12, 70.07, 69.41, 68.64, 66.27, 58.93, 50.97. HRMS (ESI) m/z: calc'd for $C_{20}H_{25}Cl_3N_4O_8Na$:577.0636, found: 577.0634.

Synthesis of compound 1 and 2.

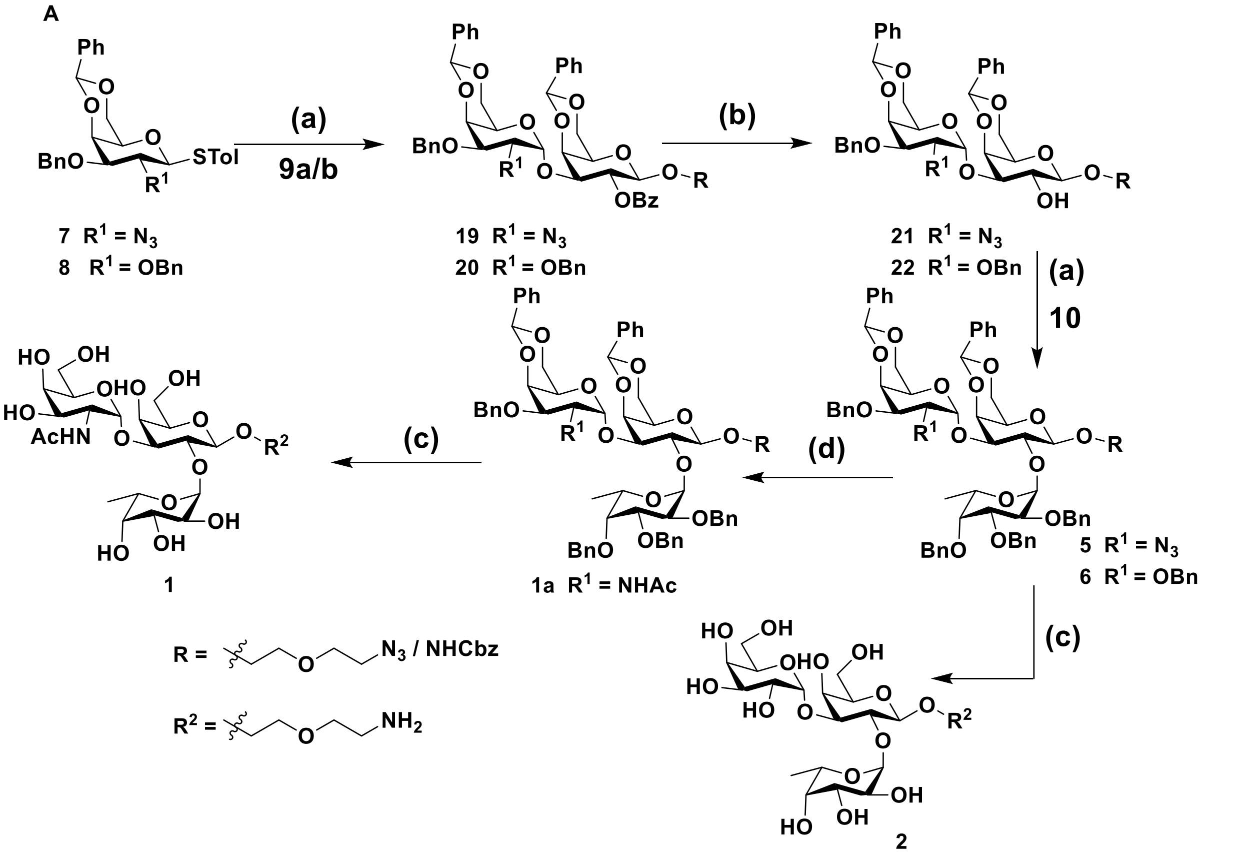

Figure 2. Synthesis of compound **1-2**: (a) NIS/TfOH, DCM , -40 to -20 ˚C, 4 Å molecular sieves with Comp **9** (**7** & **8**) and Comp **10 (21,** & **22**); (b) NaOMe, MeOH; (c) HCOOH, $Pd(OH)_2/H_2$, MeOH; (d) Zn, THF:AcOH:Ac_2O(v/v/v, 3:2:1).

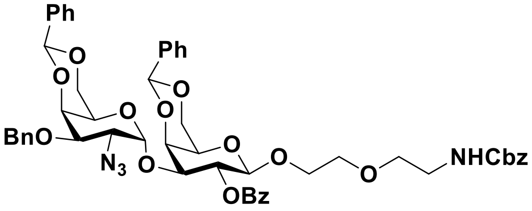

19

Compound **7** (0.3 g, 0.61 mmol) and **9b** (0.315 g, 0.6 mmol) were dissolved in dry CH_2Cl_2 (24 mL) and freshly dried 4 Å MS were added under anhydrous conditions. After stirring for 1 h at RT, the reaction flask was cooled at -78 °C and NIS (0.21 g, 0.92 mmol) and TfOH (0.011 mL, 0.122 mmol) were added and monitored the reaction by TLC. After the completion of reaction, quenched with Et_3N and diluted with CH_2Cl_2. Molecular sieves were filtered off on celite bed and organic layer washed with $Na_2S_2O_3$, followed by brine and dried over Na_2SO_4, then filtered, concentrated under reduced pressure. The residue was purified by column chromatography (EtOAc/Hexane = 1/1) to afford **19** (0.35 g, 61%). ^{1}H NMR (400 MHz, Chloroform-*d*) δ 8.01 (d, *J* = 7.7 Hz, 2H), 7.61 – 7.56 (m, 3H), 7.45 – 7.36 (m, 8H), 7.33 – 7.26 (dt, *J* = 20.8, 3.6 Hz, 7H), 5.61 (d, *J* = 13.6 Hz, 2H), 5.22 (s, 1H), 5.13 – 5.06 (m, 4H), 4.72 (d, *J* = 8.0 Hz, 1H), 4.65 (d, *J* = 11.7 Hz, 1H), 4.55 (d, *J* = 11.7 Hz, 1H), 4.38 (d, *J* = 12.3 Hz, 2H), 4.15 – 4.09 (m, 1H), 4.00 – 3.91 (m, 3H), 3.82 (q, *J* = 7.1, 5.3 Hz, 2H), 3.75 (dt, *J* = 10.9, 5.1 Hz, 1H), 3.64 (d, *J* = 12.5 Hz, 1H), 3.54 (t, *J* = 4.4 Hz, 2H), 3.48 (s, 1H), 3.44 – 3.35 (m, 3H), 3.12 (q, *J* = 5.5 Hz, 2H). ^{13}C NMR (100 MHz, Chloroform-*d*) δ 165.04, 156.35, 137.82, 137.63, 137.53, 136.71, 133.48, 129.82, 129.54, 128.89, 128.62, 128.51, 128.34, 128.09, 128.08, 127.80, 127.78, 126.35, 126.05, 101.18, 100.97, 100.60, 98.41, 77.70, 77.22, 74.07, 73.16, 72.90, 71.47, 70.39, 70.10, 69.98, 69.00, 68.80, 68.00, 66.76, 66.61, 63.35, 58.47, 40.82, 29.69. HRMS (ESI) m/z: calc'd for $C_{52}H_{54}N_4O_{14}Na$: 981.3534, found: 981.3541.

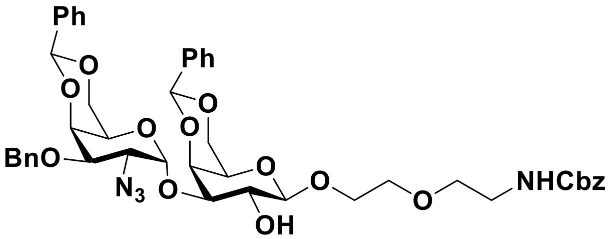

21

Compound **19** (0.33 g, 0.34 mmol) was dissolved in mixture of dry methanol and dry CH_2Cl_2 (15 mL, 2/1, v/v). Sodium methoxide (0.036g, 0.66mmol) was added under nitrogen atmosphere. After completion of reaction, neutralized with Amebrlite IR-120H$^+$ acid resin, filtered and solvent was evaporated. The residue was purified by column chromatography (EtOAc/Hexane = 1.5/1) to afford **21** (0.22 g, 77%). ^{1}H NMR (400 MHz, Chloroform-*d*) δ 7.55 (dd, *J* = 15.5, 6.9 Hz, 4H), 7.43 – 7.29 (m, 16H), 5.73 (q, *J* = 7.3, 5.9 Hz, 1H), 5.63 – 5.57 (m, 1H), 5.48 – 5.43 (m, 1H), 5.28 – 5.06 (m, 3H), 4.76 – 4.56 (m, 2H), 4.37 (dd, *J* = 17.8, 9.9 Hz, 2H), 4.29 – 4.18 (m, 3H), 4.14 – 4.05 (m, 3H), 4.01 (s, 1H), 3.96 – 3.84 (m, 3H), 3.76 – 3.56 (m, 6H), 3.52 – 3.32 (m, 4H). ^{13}C NMR (100 MHz, Chloroform-*d*) δ 156.68, 138.00, 137.79, 137.76, 136.83, 129.07, 128.90, 128.71, 128.53, 128.30, 128.16, 127.97, 127.86, 126.32, 126.25, 103.58, 101.09, 100.87, 96.74, 77.77, 77.36, 74.07, 73.10, 72.82, 71.39, 70.26, 70.21, 69.49, 69.41, 69.25, 68.72, 66.78, 63.34, 58.38, 41.04. HRMS (ESI) m/z: calc'd for $C_{45}H_{50}N_4O_{13}Na$: 877.3271, found: 877.3270.

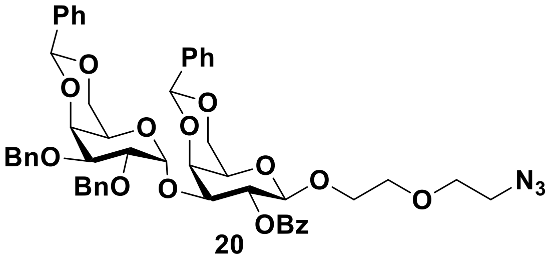

20

Compound **8** (0.3 g, 0.54 mmol) and **9a** (0.29 g, 0.59 mmol) were dissolved in dry CH_2Cl_2 (10 mL), and freshly dried 4 Å MS were added under anhydrous conditions. After stirring for 1 h at RT, the reaction flask was cooled at -78 °C and NIS (0.18 g, 0.81 mmol) and TfOH (0.01 mL, 0.11 mmol) were added and monitored the reaction by using TLC. After the completion of

reaction, quenched with Et_3N and diluted with CH_2Cl_2. Molecular sieves was filtered on celite bed and organic layer washed with $Na_2S_2O_3$, followed by brine and dried over Na_2SO_4, then filtered, concentrated under reduced pressure. The residue was purified by column chromatography (EtOAc/Hexane = 1/2) to afford **20** (0.31 g, 63%). ^{1}H NMR (400 MHz, Chloroform-*d*) δ 8.07 – 8.04 (m,z 2H), 7.63 – 7.59 (m, 1H), 7.55 (dd, *J* = 6.7, 2.9 Hz, 2H), 7.49 – 7.44 (m, 4H), 7.34 (ddd, *J* = 11.1, 5.2, 2.1 Hz, 6H), 7.31 – 7.28 (m, 3H), 7.26 – 7.21 (m, 7H), 5.62 (dd, *J* = 10.1, 8.0 Hz, 1H), 5.28 (s, 1H), 5.26 (s, 1H), 5.12 (d, *J* = 3.4 Hz, 1H), 4.83 (d, *J* = 11.3 Hz, 1H), 4.76 (d, *J* = 8.0 Hz, 1H), 4.71 (d, *J* = 12.1 Hz, 1H), 4.59 (dd, *J* = 11.7, 3.3 Hz, 2H), 4.34 (dd, *J* = 12.3, 1.6 Hz, 1H), 4.26 (d, *J* = 3.6 Hz, 1H), 4.07 – 4.00 (m, 2H), 3.97 – 3.87 (m, 4H), 3.79 (ddd, *J* = 11.2, 7.3, 3.6 Hz, 1H), 3.65 – 3.58 (m, 2H), 3.55 – 3.48 (m, 5H), 3.44 (s, 1H), 3.16 – 3.01 (m, 2H). ^{13}C NMR (100 MHz, Chloroform-*d*) δ 165.27, 138.94, 138.80, 137.89, 133.46, 130.09, 129.96, 129.73, 128.94, 128.91, 128.66, 128.48, 128.37, 128.34, 128.19, 127.99, 127.74, 127.64, 127.60, 126.56, 126.45, 126.38, 101.39, 101.00, 100.91, 99.33, 78.48, 77.36, 76.18, 75.54, 74.69, 73.52, 73.48, 72.37, 70.71, 70.14, 69.15, 69.03, 68.75, 66.88, 63.43, 50.74. HRMS (ESI) m/z: calc'd for $C_{51}H_{53}N_3O_{13}Na$: 938.3476, found: 938.3453.

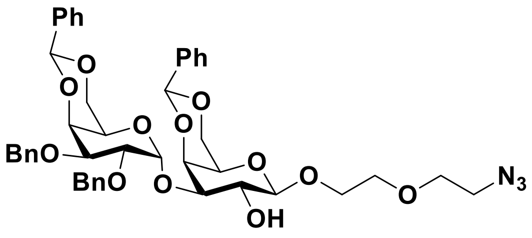

22

Compound **20** (0.3 g, 0.32 mmol) was dissolved in mixture of dry methanol and dry CH_2Cl_2 (15 mL, 2/1, v/v). Sodium methoxide (0.035 g, 0.65 mmol) was added under nitrogen atmosphere. After completion of reaction, neutralized with Amebrlite IR-120H$^+$ acid resin, filtered and solvent were evaporated. The residue was purified by column chromatography (EtOAc/Hexane = 1.5/1) to afford **22** (0.2 g, 75%). ^{1}H NMR (400 MHz, Chloroform-*d*) δ 7.54 (dt, *J* = 8.0, 2.0 Hz, 4H), 7.41 – 7.38 (m, 3H), 7.37 – 7.35 (m, 2H), 7.34 – 7.32 (m, 2H), 7.32 – 7.30 (m, 2H), 7.30 – 7.27 (m, 2H), 7.23 – 7.17 (m, 1H), 7.17 – 7.14 (m, 4H), 5.54 (s, 1H), 5.51 (s, 1H), 5.30 (d, *J* = 2.2 Hz, 1H), 4.82 (d, *J* = 12.1 Hz, 1H), 4.76 – 4.71 (m, 2H), 4.62 (d, *J* = 11.7 Hz, 1H), 4.43 (d, *J*

= 7.7 Hz, 1H), 4.37 – 4.32 (m, 2H), 4.27 – 4.22 (m, 2H), 4.16 – 4.14 (m, 2H), 4.13 – 4.07 (m, 3H), 4.03 (ddd, J = 7.3, 5.4, 1.9 Hz, 3H), 3.84 – 3.78 (m, 1H), 3.76 – 3.70 (m, 6H), 3.44 – 3.40 (m, 3H), 3.05 (bs, 1H). ^{13}C NMR (100 MHz, Chloroform-*d*) δ 139.03, 138.74, 138.05, 137.87, 129.04, 128.96, 128.37, 128.27, 128.24, 127.84, 127.75, 127.59, 127.49, 126.53, 126.45, 103.69, 101.31, 101.16, 95.16, 77.36, 76.25, 75.94, 75.30, 74.80, 72.81, 72.41, 72.20, 70.62, 70.16, 69.59, 69.45, 69.21, 68.64, 66.88, 63.10, 50.91.HRMS (ESI) m/z: calc'd for $C_{44}H_{49}NO_{12}Na$:834.3213, found: 834.3214.

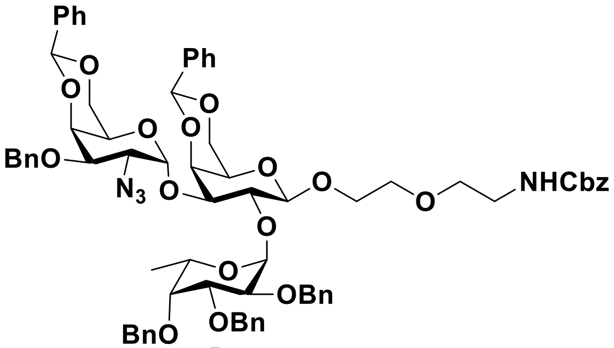

5

Compound **10** (0.15 g, 0.27 mmol) and **21** (0.17 g, 0.194 mmol) were dissolved in dry CH_2Cl_2 (5mL), and freshly dried 4 Å MS were added under anhydrous conditions. After stirring for 1 h at RT, the reaction flask was cooled at -40 °C and NIS (0.093 g, 0.41 mmol), TfOH (0.008 mL, 0.055 mmol) were added and monitored the reaction by TLC. After the completion of reaction, quenched with Et_3N and diluted with CH_2Cl_2. Molecular sieves were filtered off on celite bed and organic layer washed with $Na_2S_2O_3$, followed by brine and dried over Na_2SO_4, then filtered, concentrated under reduced pressure. The residue was purified by column chromatography (EtOAc/Hexane = 1/1) to afford **5** (0.19 g, 53%). ^{1}H NMR (400 MHz, Chloroform-*d*) δ 7.54 (d, J = 8.0 Hz, 2H), 7.43 – 7.40 (m, 6H), 7.37 – 7.30 (m, 14H), 7.29 – 7.18 (m, 18H), 5.47 (s, 1H), 5.31 – 5.23 (m, 3H), 5.11 (d, J = 12.0 Hz, 1H), 5.03 (d, J = 2.4 Hz, 2H), 4.92 (t, J = 10.7 Hz, 2H), 4.73 – 4.61 (m, 5H), 4.45 – 4.38 (m, 2H), 4.31 – 4.28 (m, 2H), 4.21 – 4.14 (m, 2H), 4.02 – 3.94 (m, 4H), 3.89 – 3.76 (m, 4H), 3.71 (s, 2H), 3.66 – 3.58 (m, 3H), 3.53 (q, J = 7.0 Hz, 1H), 3.46 (t, J = 5.0 Hz, 2H), 3.39 – 3.25 (m, 3H), 2.83 (d, J = 12.4 Hz, 1H), 1.16 (d, J = 6.4 Hz, 3H). ^{13}C NMR (100 MHz, Chloroform-*d*) δ 156.54, 139.35, 139.13, 138.90, 138.47, 137.99, 137.77, 137.53, 136.67, 129.06, 128.85, 128.68, 128.61, 128.50 0, 128.36, 128.26, 128.21, 127.97, 127.78, 127.72, 127.61, 127.03 , 126.30, 126.11, 101.88, 100.68, 100.58, 98.08, 93.76, 80.44,

77.77, 77.37, 76.11, 74.97, 74.23, 73.75, 73.18, 72.41, 71.66, 71.57, 71.20, 70.17, 69.95, 69.39, 68.78, 67.89, 66.86, 66.59, 66.30, 63.33, 58.91, 41.04, 29.84, 16.90.

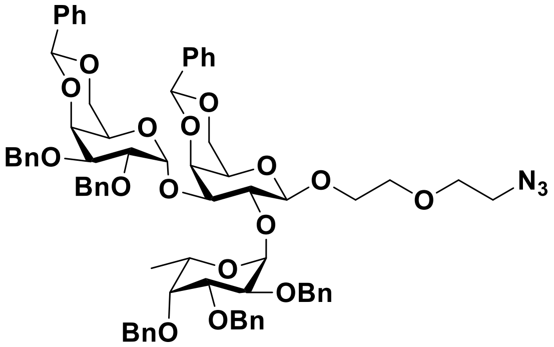

6

Compound **10** (0.13 g, 0.24 mmol) and **22** (0.16 g, 0.192 mmol) were dissolved in dry CH_2Cl_2 (24 mL), and freshly dried 4 Å MS were added under anhydrous conditions. After stirring for 1 h at RT, the reaction flask was cooled at -40 °C and NIS (0.081 g, 0.36 mmol), TfOH (0.004 mL, 0.048 mmol) were added and monitored the reaction by using TLC. After the completion of reaction, quenched with Et_3N and diluted with CH_2Cl_2. Molecular sieves were filtered off on celite bed and organic layer washed with $Na_2S_2O_3$, followed by brine and dried over Na_2SO_4, then filtered, concentrated under reduced pressure. The residue was purified by column chromatography (EtOAc/Hexane = 1/1) to afford **6** (0.12 g, 42%). ^{1}H NMR (400 MHz, Chloroform-*d*) δ 7.54 – 7.49 (m, 2H), 7.44 – 7.37 (m, 11H), 7.37 – 7.23 (m, 17H), 7.20 – 7.07 (m, 5H), 5.49 (s, 1H), 5.06 (d, *J* = 3.3 Hz, 1H), 5.01 (d, *J* = 10.1 Hz, 1H), 4.87 – 4.75 (m, 6H), 4.69 (s, 2H), 4.62 – 4.48 (m, 4H), 4.39 (d, *J* = 7.7 Hz, 1H), 4.32 (d, *J* = 12.3 Hz, 1H), 4.15 (d, *J* = 3.6 Hz, 1H), 4.13 – 4.03 (m, 4H), 3.97 – 3.88 (m, 3H), 3.73 (dd, *J* = 9.8, 3.6 Hz, 1H), 3.65 – 3.37 (m, 11H), 3.32 – 3.22 (m, 2H), 1.20 (d, *J* = 6.2 Hz, 3H). ^{13}C NMR (100 MHz, Chloroform-*d*) δ 139.42, 139.36, 139.18, 139.09, 138.72, 138.62, 138.03, 129.15, 128.74, 128.61, 128.55, 128.32, 128.13, 128.11, 128.03, 127.88, 127.77, 127.67, 127.61, 127.45, 127.38, 127.17, 126.52, 126.43, 104.40, 102.36, 101.03, 100.15, 96.48, 82.60, 79.99, 78.51, 77.36, 76.19, 75.79, 75.72, 75.52, 75.09, 74.30, 73.98, 73.24, 73.13, 72.61, 71.21, 70.39, 69.79, 69.73, 69.42, 69.22, 69.08, 66.65, 62.61, 50.87, 17.09. HRMS (ESI) m/z: calc'd for $C_{71}H_{77}O_{16}N_3Na$:1250.5201, found: 1250.5186.

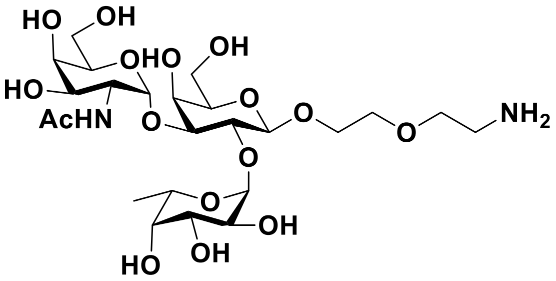

1

Compound **5** (0.042 g, 0.033 mmol) was dissolved in dry THF (10 mL), zinc dust (0.040 g, 0.611 mmol) and acetic anhydride (0.015 mL, 0.165 mmol) was added and stirred at room temperature for 6 h, the reaction mixture was immediately filtered through celite and the filtrate was extracted with EtOAc and washed with $NaHCO_3$ (3 x 20 mL). The organic layers were combined, washed with brine, dried over Na_2SO_4 and concentrated under reduced pressure. The crude was dissolved in dry methanol (3 mL), 20% $Pd(OH)_2$ on carbon (0.030 g) and 10 µL formic acid were added was added and purged with a hydrogen gas, and the mixture was stirred at room temperature for 2 d. The mixture was filtered through Celite, and the filtrate was evaporated under reduced pressure. The residue was purified through bond elute C-18 column eluted with water. The product fraction was lyophilized to afford compound **1** (0.008 g, 40%.) as a white powder.^{1}H NMR (600 MHz, Deuterium Oxide) δ 5.29 (d, *J* = 3.7 Hz, 1H), 5.17 (d, *J* = 3.7 Hz, 1H), 4.62 (d, *J* = 5.8 Hz, 1H), 4.57 (d, *J* = 7.8 Hz, 1H), 4.43 (q, *J* = 6.7 Hz, 1H), 4.24 – 4.21 (m, 2H), 4.08 (d, *J* = 8.0 Hz, 1H), 3.99 (d, *J* = 3.1 Hz, 1H), 3.99 – 3.91 (m, 2H), 3.87 – 3.84 (m, 1H), 3.82 (d, *J* = 9.0 Hz, 2H), 3.78 – 3.74 (m, 8H), 3.66 (q, *J* = 5.9, 5.0 Hz, 2H), 3.22 – 3.18 (m, 3H), 2.04 (s, 3H), 1.23 (d, *J* = 6.6 Hz, 3H). ^{13}C NMR (150 MHz, Deuterium Oxide) δ 101.65, 101.39, 98.60, 91.28, 76.22, 75.57, 74.87, 72.56, 72.44, 71.91, 71.04, 70.75, 70.11, 69.70, 69.68, 69.15, 68.48, 67.69, 66.83, 66.30, 65.16, 62.91, 61.31, 61.00, 49.49, 46.63, 39.03, 21.90, 15.32.

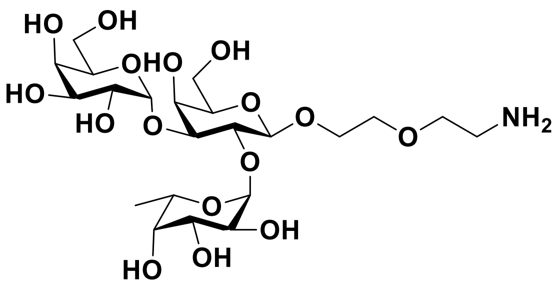

2

Compound **6** (0.04 g) was dissolved in dry methanol (3 mL), 20% $Pd(OH)_2$ (0.035 g) and 10 µL formic acid were added and purged with a hydrogen gas, and the mixture was stirred at room temperature for 2 days. The whole mixture was filtered through celite, and the filtrate was evaporated under reduced pressure. The residue was purified through a bond elute C-18 column eluted with water. The product fraction was lyophilized to afford compound **2** (0.015 g, 78%.) as a white powder. ^{1}H NMR (400 MHz, Deuterium Oxide) δ 5.09 (d, *J* = 3.9 Hz, 1H), 4.62 – 4.56 (m, 3H), 4.16 (d, *J* = 2.7 Hz, 1H), 4.08 (ddd, *J* = 11.4, 5.7, 3.5 Hz, 1H), 4.02 – 3.95 (m, 2H), 3.90 – 3.80 (m, 4H), 3.78 – 3.73 (m, 6H), 3.72 – 3.61 (m, 7H), 3.45 (dd, *J* = 10.0, 7.7 Hz , 1H), 3.19 (t, *J* = 5.2 Hz, 2H), 1.21 (d, *J* = 6.5 Hz, 3H). ^{13}C NMR (100 MHz, Deuterium Oxide) δ 103.25, 102.93, 94.98, 75.92, 75.30, 74.77, 72.52, 71.41, 71.39, 70.54, 70.46, 69.71, 69.38, 69.19, 68.32, 66.55, 64.82, 60.98, 60.93, 39.19, 15.80. HRMS (ESI) m/z: calc'd for $C_{22}H_{42}NO_{16}H$: 576.2504, found: 576.2503.

Synthesis of compound 3 and 4.

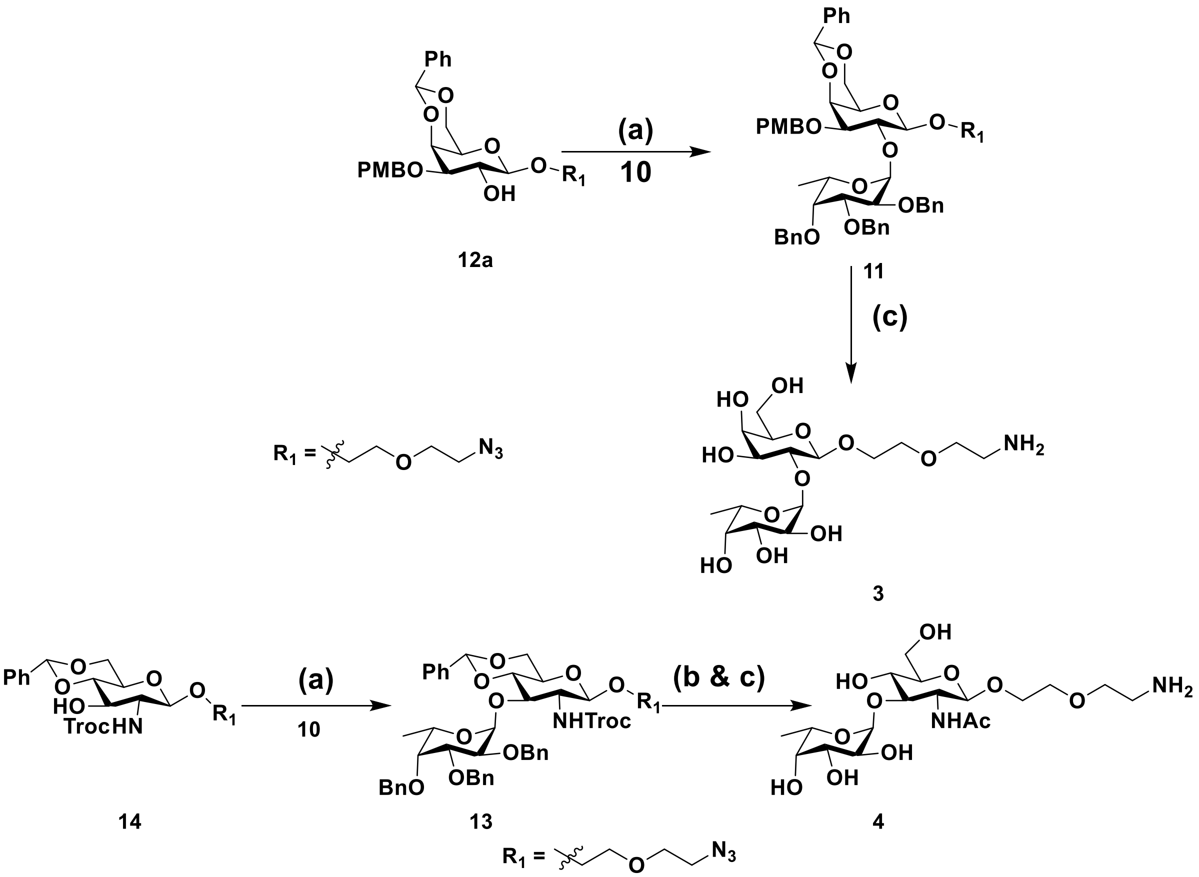

Figure S5. Synthesis of comp **3 and 4**: (a) NIS/TfOH, DCM , -40 to -20 ˚C, 4 Å molecular sieves with Comp **12a (10)** and Comp **14 (10)**; (b) LiOH.H_2O, 1,4-*d*ioxane:H_2O (v/v, 1:1), 80 ºC (c) HCOOH, $Pd(OH)_2/H_2$, MeOH.

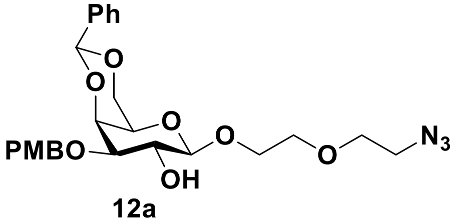

12a

Compound **12** (0.8 g, 1.32 mmol) was dissolved in mixture of dry methanol and CH_2Cl_2 (15 mL, 2/1, v/v). Sodium methoxide (0.14 g, 2.64 mmol) was added under nitrogen atmosphere. After

stirring overnight the reaction mixture was neutralized with Amebrlite IR-120H$^+$ acid resin, filtered and solvent were evaporated. The residue was purified by column chromatography (EtOAc/Hexane = 1/1) to afford **12a** (0. 5 g, 75%). ^{1}H NMR (400 MHz, Chloroform-*d*) δ 7.52 (dd, *J* = 7.4, 1.9 Hz, 2H), 7.38 – 7.31 (m, 5H), 6.86 (d, *J* = 8.6 Hz, 2H), 5.45 (s, 1H), 4.70 (s, 2H), 4.38 (d, *J* = 7.8 Hz, 1H), 4.29 (dd, *J* = 12.4, 1.6 Hz, 1H), 4.09 – 3.98 (m, 4H), 3.79 (s, 3H), 3.78 – 3.72 (m, 2H), 3.69 (dd, *J* = 7.7, 3.5Hz, 3H), 3.48 (dd, *J* = 9.7, 3.5 Hz, 1H), 3.40 – 3.36 (m, 2H), 3.36 (s, 1H), 2.79 (bs, 1H).^{13}C NMR (100 MHz, Chloroform-*d*) δ 159.47, 137.9, 130.4 , 129.60, 129.03, 128.23, 126.52, 113.97, 103.51, 101.28, 78.79, 73.54, 71.40, 70.60, 70.19, 70.13, 69.41, 68.63, 66.91, 55.42, 50.88.

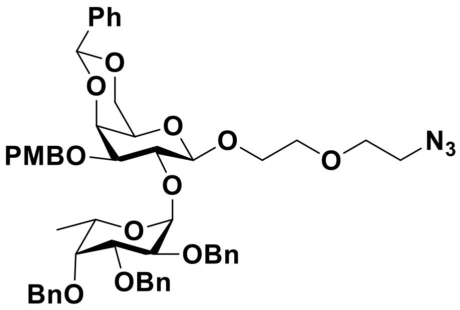

11

Compound **10** (0.11 g, 0.20 mmol) and **12a** (0.09 g, 0.183 mmol), were dissolved in dry CH_2Cl_2 (3 mL), and freshly dried 4 Å MS were added under anhydrous conditions. After stirring for 1 h at RT, the reaction flask mixture was cooled at -40 °C and NIS (0.068 g, 0.305 mmol), TfOH (0.004 mL, 0.041 mmol) were added and monitored the reaction by TLC. After the completion of reaction, quenched with Et_3N and diluted with CH_2Cl_2. Molecular sieves were filtered off on ciliate and organic layer washed with $Na_2S_2O_3$, followed by brine and dried over Na_2SO_4, then filtered, concentrated under reduced pressure. The residue was purified by column chromatography (EtOAc/Hexane = 1/4) to afford **11** (0.13 g, 69%). ^{1}H NMR (400 MHz, Chloroform-*d*) δ 7.52 (dd, *J* = 7.7, 1.7 Hz, 2H), 7.40 – 7.37 (m, 3H), 7.36 – 7.32 (m, 3H), 7.32 – 7.27 (m, 5H), 7.26 – 7.24 (m, 2H), 7.23 – 7.16 (m, 7H), 6.78 – 6.75 (m, 2H), 5.59 (d, *J* = 3.6 Hz, 1H), 5.40 (s, 1H), 4.94 (d, *J* = 11.6 Hz, 1H), 4.87 (d, *J* = 11.7 Hz, 1H), 4.74 (d, *J* = 11.7 Hz, 1H), 4.67 (t, *J* = 12.4 Hz, 2H), 4.56 (d, *J* = 12.0 Hz, 1H), 4.52 – 4.48 (m, 2H), 4.41 (q, *J* = 6.4 Hz, 1H), 4.30 – 4.27 (m, 1H), 4.16 (dd, *J* = 9.4, 7.9 Hz, 1H), 4.10 (d, *J* = 3.5 Hz, 1H), 4.04 – 3.95 (m,

4H), 3.80 – 3.78 (m, 1H), 3.76 (s, 3H), 3.69 – 3.61 (m, 3H), 3.59 – 3.51 (m, 3H), 3.34 (s, 1H), 3.26 -3.23 (m, 3H), 1.09 (d, *J* = 6.4 Hz, 3H). ^{13}C NMR (100 MHz, Chloroform-*d*) δ 159.12, 139.22, 138.9, 138.64, 137.80, 130.51, 128.90, 128.72, 128.45, 128.37, 128.18, 127.91, 127.51, 127.38, 126.35, 113.73, 101.80, 100.99, 97.24, 81.23, 79.62, 77.87, 76.09, 74.66, 73.08, 72.94, 72.57, 71.95, 70.51, 70.45, 69.85, 69.39, 67.90, 66.45, 66.25, 55.35, 50.80, 29.79, 16.77.

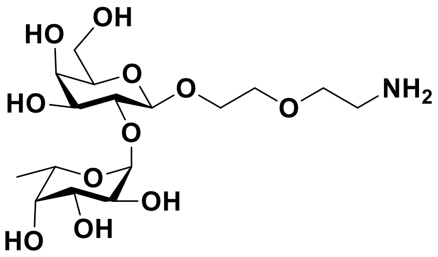

3

Compound **11** (0.035 g) was dissolved in dry methanol (3 mL), 20% $Pd(OH)_2$ on carbon (0.025 g) and formic acid (10 µL) were added and purged with a hydrogen gas, and the mixture was stirred at room temperature for 2 days. The mixture was filtered through celite, and the filtrate was evaporated under reduced pressure. The residue was purified through bond elute C-18 column eluted with water. The product fraction was lyophilized to afford compound **3** (0.011 g, 73%.) as a white powder. ^{1}H NMR (400 MHz, Deuterium Oxide) δ 5.10 (d, *J* = 3.9 Hz, 1H), 4.42 (d, *J* = 7.8 Hz, 1H), 4.21 (q, *J* = 6.6 Hz, 1H), 4.01 – 3.93 (m, 1H), 3.80 (d, *J* = 3.5 Hz, 1H), 3.74 (dt, *J* = 9.5, 3.1 Hz, 3H), 3.69 – 3.62(m, 8H), 3.58 (dd, *J* = 7.8, 4.4 Hz, 1H), 3.48 (dd, *J* = 9.6, 7.8 Hz, 1H), 3.16 – 3.06 (m, 2H), 1.10 (d, *J* = 6.6 Hz, 3H). ^{13}C NMR (100 MHz, Deuterium Oxide) δ 101.85, 99.61, 77.26, 75.04, 73.53, 71.91, 69.81, 69.59, 69.16, 68.87, 68.41, 66.86, 66.36, 61.02, 39.14, 15.47. HRMS (ESI) m/z: calc'd for $C_{16}H_{32}NO_{11}H$:414.1975, found: 414.1976.

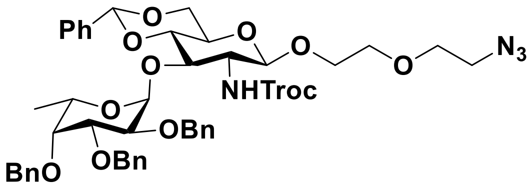

13

Compound **10** (0.11 g, 0.20 mmol) and **14** (0.09 g, 0.163 mmol) were dissolved in dry CH_2Cl_2 (3 mL), and freshly dried 4 Å MS were added under anhydrous conditions. After stirring for 1 h at RT, the reaction flask mixture was cooled at -40 °C. NIS (0.068 g, 0.305 mmol), TfOH (0.004 mL, 0.041 mmol) were added and monitored the reaction by TLC. After the completion of reaction, quenched with Et_3N and diluted with CH_2Cl_2. Molecular sieves were filtered off on ciliate and organic layer washed with $Na_2S_2O_3$, followed by brine and dried over Na_2SO_4, then filtered, concentrated under reduced pressure. The residue was purified by column chromatography (EtOAc/Hexane = 1/3) to afford **13** (0.11 g, 55%). ^{1}H NMR (400 MHz, Chloroform-*d*) δ 7.47 (dd, *J*= 6.7, 3.0 Hz, 2H), 7.43 – 7.28 (m, 18H), 5.61 (s, 1H), 5.52 (s, 1H), 5.22 (d, *J* = 3.7 Hz, 1H), 4.93 (d, *J* = 11.5 Hz, 2H), 4.86 – 4.73 (m, 5H), 4.59 (d, *J* = 11.5 Hz, 2H), 4.36 (dd, *J* = 10.5, 4.9 Hz, 1H), 4.26 (t, *J* = 9.6 Hz, 1H), 4.14 – 4.11 (m, 1H), 4.07 (dd, *J* = 10.2, 3.7 Hz, 1H), 3.98 – 3.96 (m, 1H), 3.95 – 3.93 (m, 1H), 3.81 – 3.73 (m, 2H), 3.71 – 3.61 (m, 5H), 3.57 – 3.56 (m, 1H), 3.52 (dd, *J* = 9.6, 4.9 Hz, 1H), 3.49 – 3.39 (m, 3H), 0.81 (d, *J* = 6.4 Hz, 3H);^{13}C NMR (100 MHz, Chloroform-*d*) δ 154.19, 138.90, 138.65, 138.41, 137.28, 129.21, 128.57, 128.47, 128.40, 128.31, 128.25, 128.21, 127.83, 127.63, 127.57, 127.41, 126.31, 101.81, 97.94, 95.57, 80.38, 79.58, 77.81, 74.97, 74.49, 74.36, 73.71, 73.04, 70.76, 70.07, 69.24, 68.84, 66.86, 66.40, 59.12, 50.91, 16.30. HRMS (ESI) m/z: calc'd for $C_{47}H_{53}Cl_3N_4O_{12}Na$:993.2623, found: 993.2645.

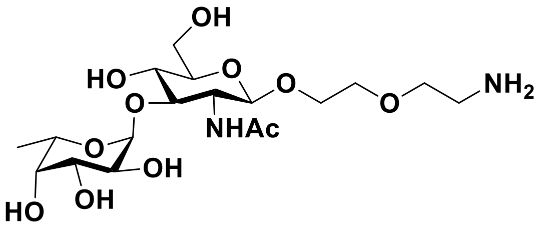

4

Compound **13** (0.10 g, 0.102 mmol) was dissolved in H_2O/Dioxane (2 mL, 1/1, v/v), LiOH.H_2O (0.043 g, 1.02 mmol) was added and stirred for 24 h at 80 ^{o}C. The reaction mixture was cooled to room temperature, neutralized by Amberlite IR120 H^+ acid resin, filtered and concentrated to dryness. Next, the crude was dissolved in MeOH followed by the addition of triethylamine and acetic anhydride (0.096 mL, 1.02 mmol) at 0 ^{o}C and stirred for another 12 h. Volatiles were evaporated and concentrated to dryness. The crude was dissolved in dry methanol (3 mL), 20% $Pd(OH)_2$ on carbon (0.025 g) and formic acid (5 µL) were added and purged with a hydrogen gas, and the mixture was stirred at room temperature for 24 h. The mixture was filtered through celite, and the filtrate was evaporated under reduced pressure. The residue was purified through bond elute C-18 column eluted with water. The product fraction was lyophilized to afford compound **4** (0.03 g, 65%.) as a white powder.^{1}H NMR (400 MHz, Deuterium Oxide) δ 4.91 (d, *J* = 4.0 Hz, 1H), 4.49 (d, *J* = 8.6 Hz, 1H), 4.24 (q, *J* = 6.6 Hz, 1H), 3.96 (dt, *J* = 11.0, 4.4 Hz, 1H), 3.86 (dd, *J* = 12.2, 1.7 Hz, 1H), 3.79 – 3.55 (m, 11H), 3.47 – 3.40 (m, 2H), 3.12 (t, *J* = 4.9 Hz, 2H), 1.94 (s, 3H), 1.08 (d, *J* = 6.6 Hz, 3H);^{13}C NMR (100 MHz, Deuterium Oxide) δ 174.68, 101.08, 99.98, 80.35, 75.92, 71.86, 69.69, 69.55, 69.29, 68.57, 67.96, 66.94, 66.44, 60.73, 55.27, 39.14, 22.28, 15.22. HRMS (ESI) m/z: calc'd for $C_{18}H_{35}N_2O_{11}H$: 455.2241, found: 455.2242.

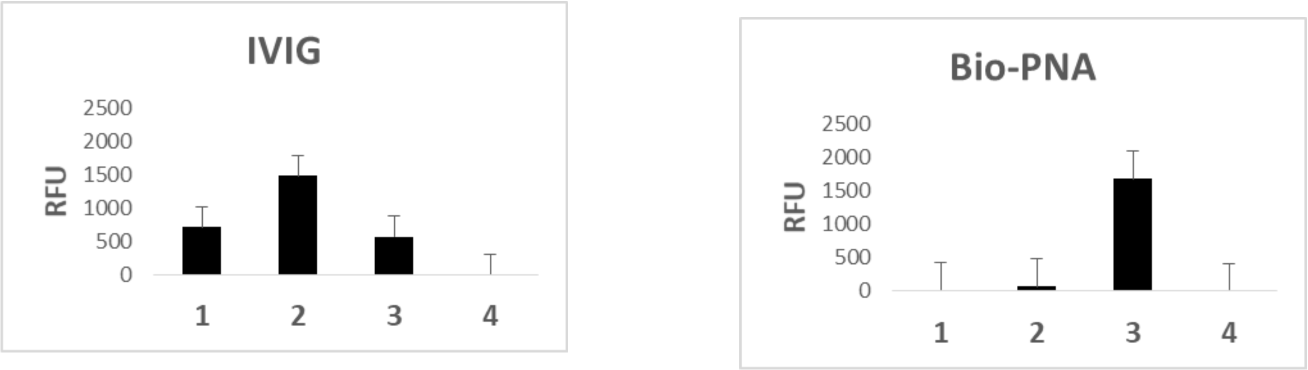

Figure S6. Microarray analysis of compounds **1-4** with IVIG and PNA lectin.

NMR DATA

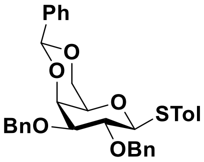
Ph
O
O
O
STol
BnO
OBn

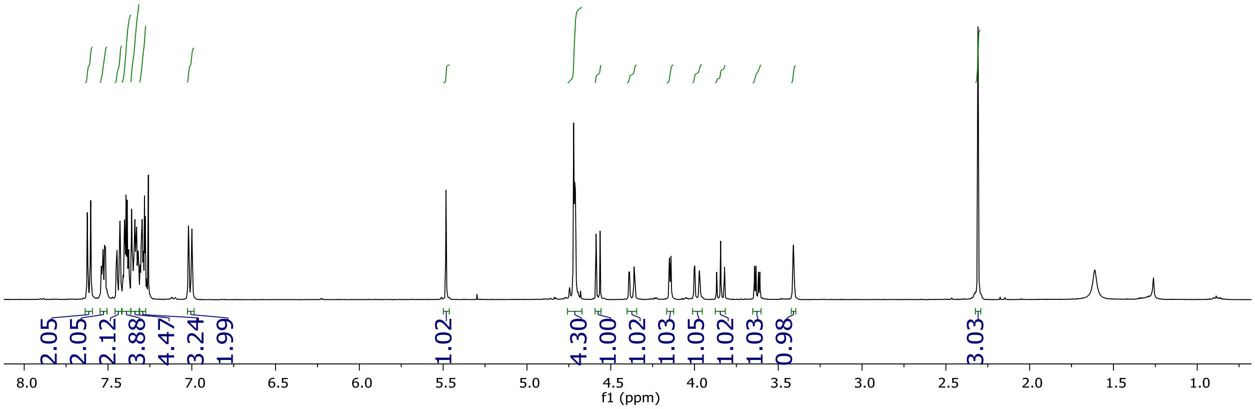
2.05
2.05
2.12
3.88
4.47
3.24
1.99
1.02
4.30
1.00
1.02
1.03
1.05
1.02
1.03
0.98
3.03
8.0 7.5 7.0 6.5 6.0 5.5 5.0 4.5 4.0 3.5 3.0 2.5 2.0 1.5 1.0
f1 (ppm)

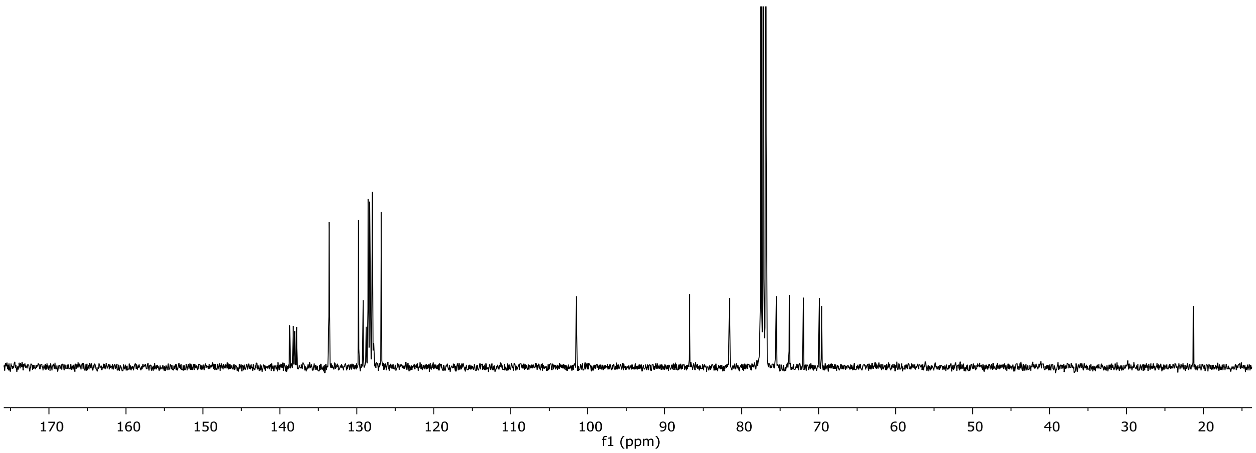
170 160 150 140 130 120 110 100 90 80 70 60 50 40 30 20
f1 (ppm)

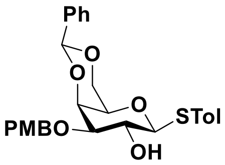
Ph
O
O
O
STol
PMBO
OH

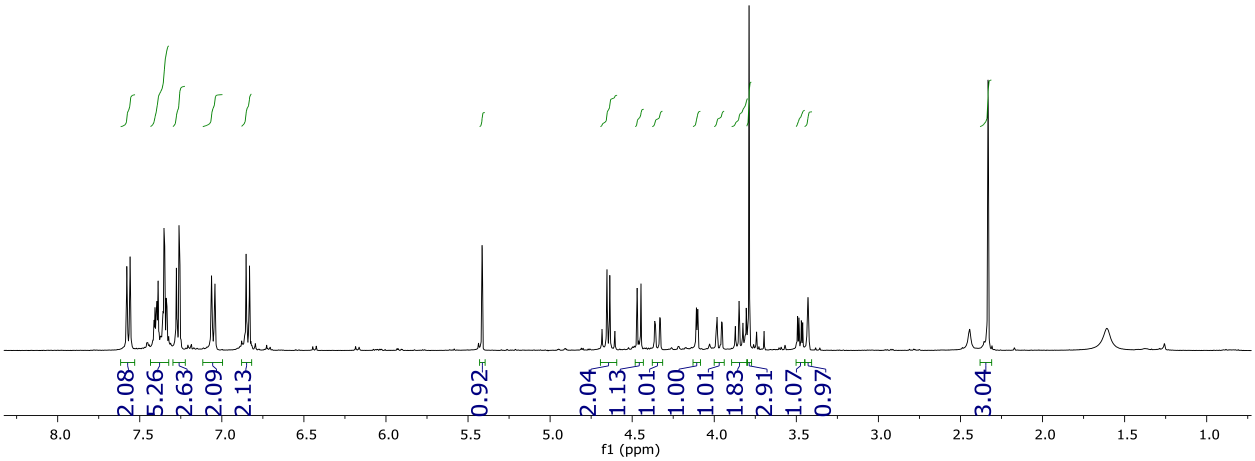
2.08
5.26
2.63
2.09
2.13
0.92
2.04
1.13
1.01
1.00
1.01
1.83
2.91
1.07
0.97
3.04
8.0 7.5 7.0 6.5 6.0 5.5 5.0 4.5 4.0 3.5 3.0 2.5 2.0 1.5 1.0
f1 (ppm)

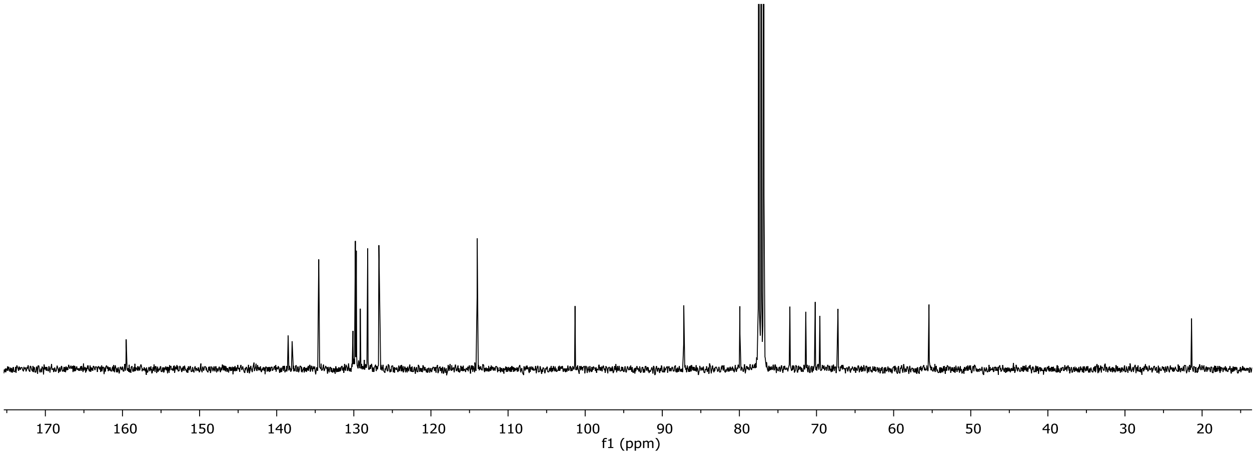
170 160 150 140 130 120 110 100 90 80 70 60 50 40 30 20
f1 (ppm)

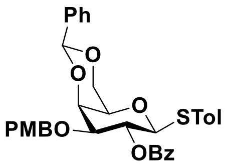
Ph
O
O
O
PMBO
STol
OBz

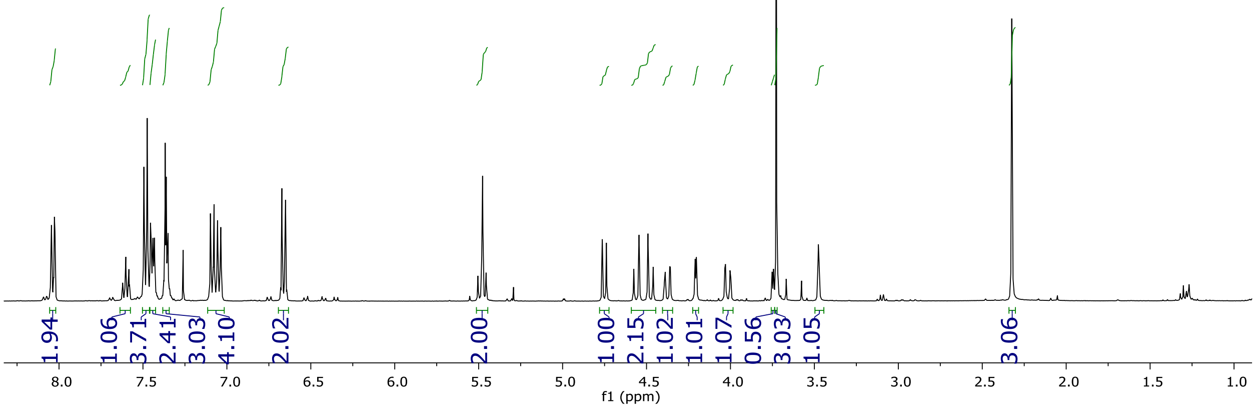
1.94
1.06
3.71
2.41
3.03
4.10
2.02
2.00
1.00
2.15
1.02
1.01
1.07
0.56
3.03
1.05
3.06
8.0 7.5 7.0 6.5 6.0 5.5 5.0 4.5 4.0 3.5 3.0 2.5 2.0 1.5 1.0
f1 (ppm)

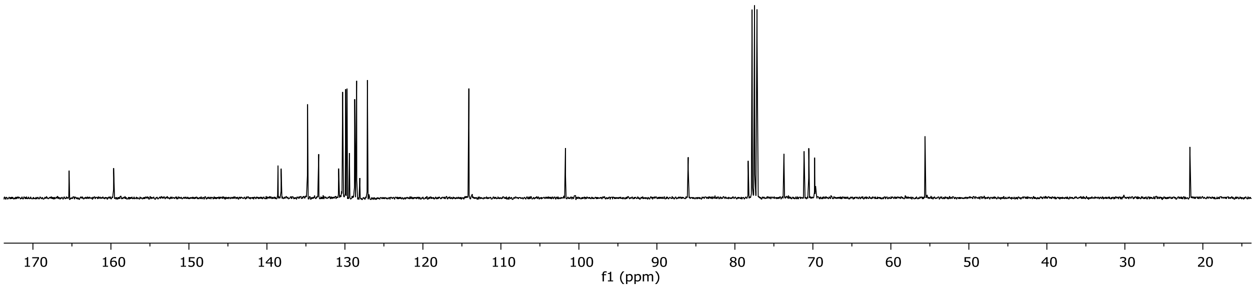
170 160 150 140 130 120 110 100 90 80 70 60 50 40 30 20
f1 (ppm)

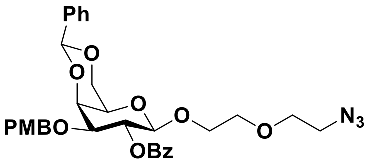
Ph
O
O
O
PMBO
OBz
O
O
N3

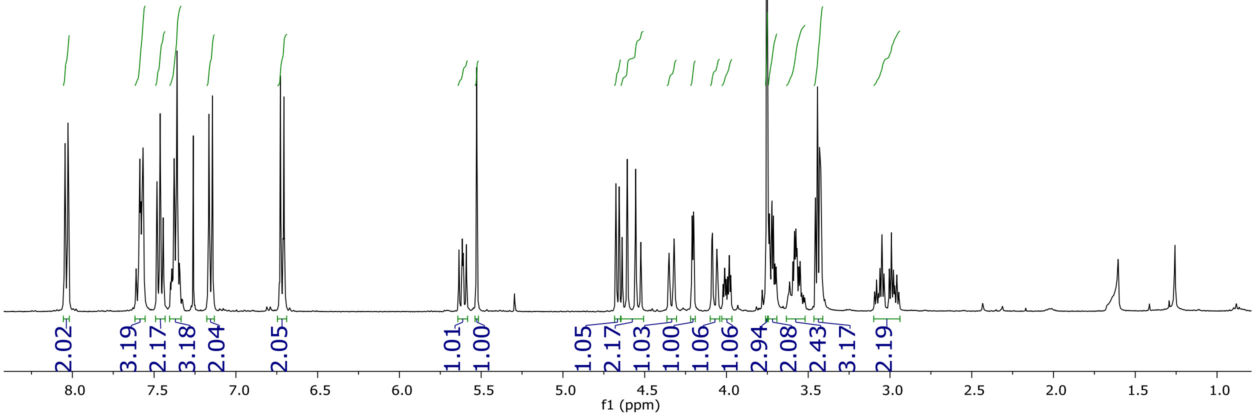
2.02
3.19
2.17
3.18
2.04
2.05
1.01
1.00
1.05
2.17
1.03
1.00
1.06
1.06
2.94
2.08
2.43
3.17
2.19
8.0 7.5 7.0 6.5 6.0 5.5 5.0 4.5 4.0 3.5 3.0 2.5 2.0 1.5 1.0
f1 (ppm)

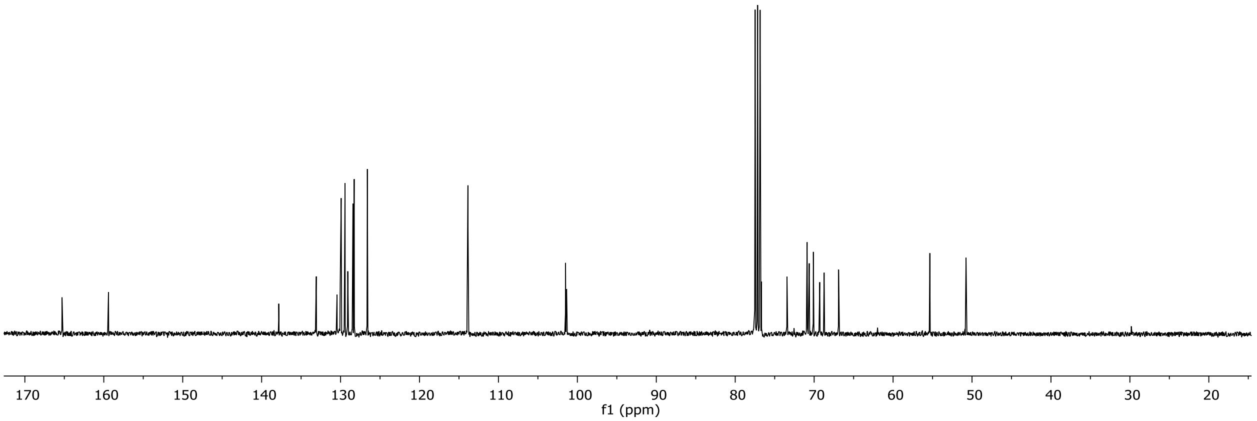
170 160 150 140 130 120 110 100 90 80 70 60 50 40 30 20
f1 (ppm)

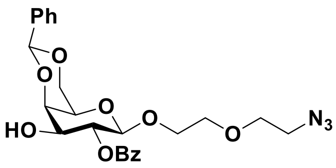
Ph
O
O
O
O
O
N3
HO
OBz

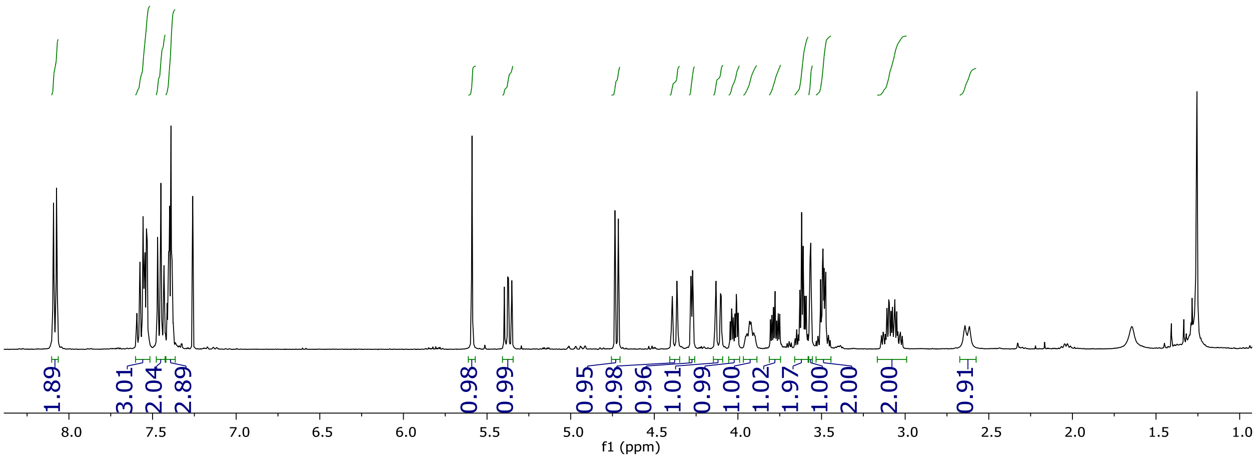
1.89
3.01
2.04
2.89
0.98
0.99
0.95
0.98
0.96
1.01
0.99
1.00
1.02
1.97
1.00
2.00
2.00
0.91
8.0
7.5
7.0
6.5
6.0
5.5
5.0
4.5
4.0
3.5
3.0
2.5
2.0
1.5
1.0
f1 (ppm)

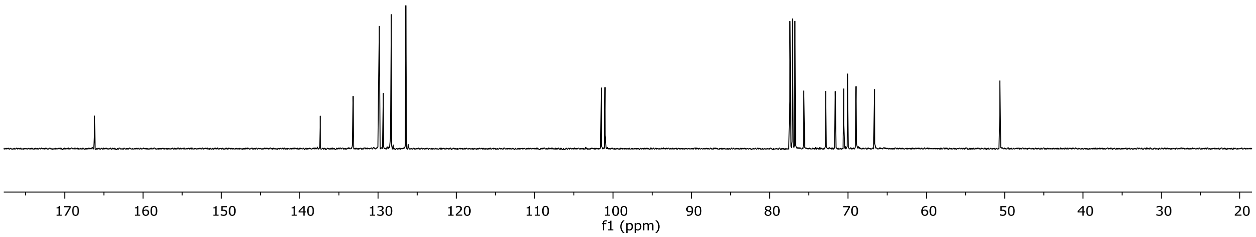
170
160
150
140
130
120
110
100
90
80
70
60
50
40
30
20
f1 (ppm)

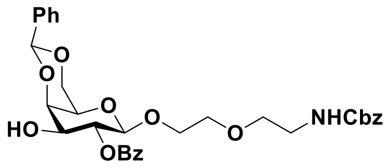
Ph
O
O
O
HO
OBz
O
O
NHCbz

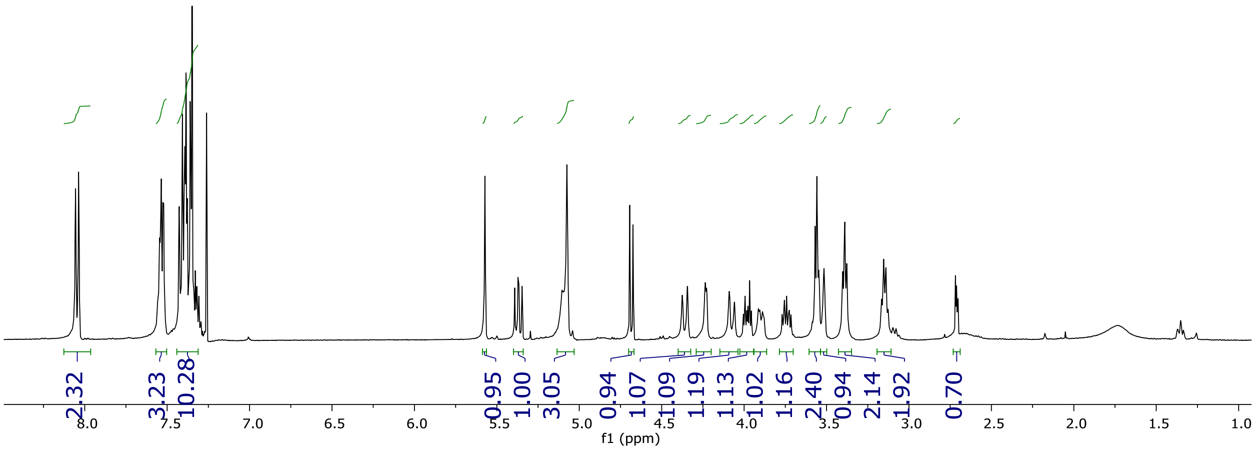
2.32
3.23
10.28
0.95
1.00
3.05
0.94
1.07
1.09
1.19
1.13
1.02
1.16
2.40
0.94
2.14
1.92
0.70
8.0
7.5
7.0
6.5
6.0
5.5
5.0
4.5
4.0
3.5
3.0
2.5
2.0
1.5
1.0
f1 (ppm)

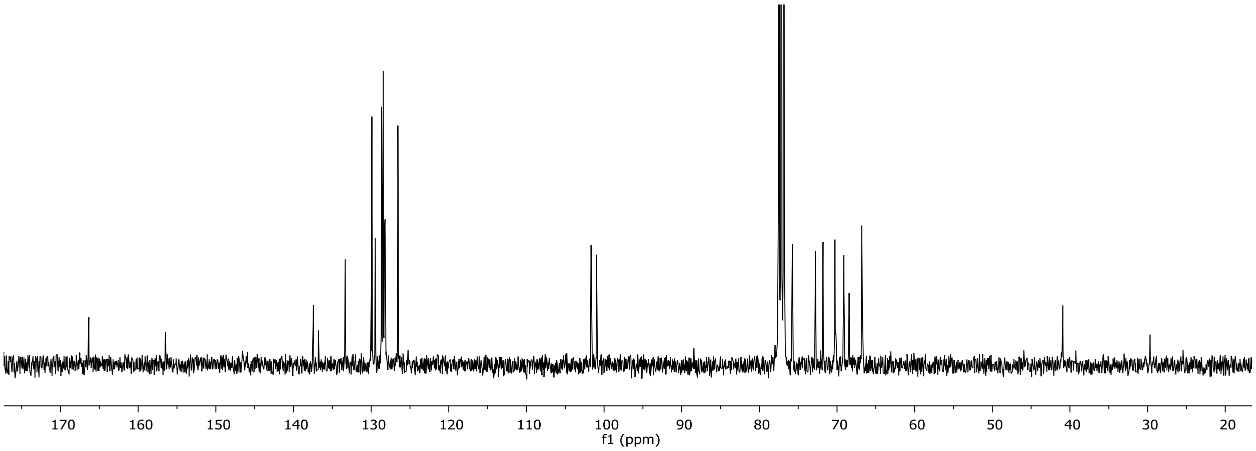
170
160
150
140
130
120
110
100
90
80
70
60
50
40
30
20
f1 (ppm)

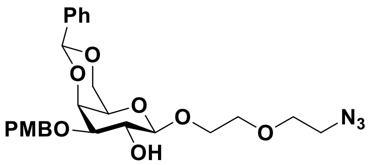
Ph
O
O
O
PMBO
OH
O
O
N3

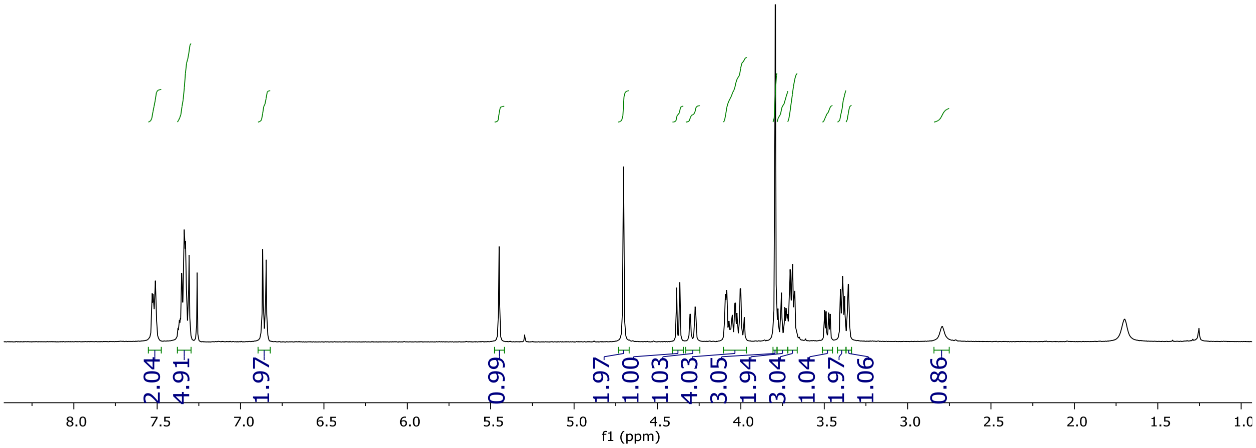
2.04
4.91
1.97
0.99
1.97
1.00
1.03
4.03
3.05
1.94
3.04
1.04
1.97
1.06
0.86
8.0 7.5 7.0 6.5 6.0 5.5 5.0 4.5 4.0 3.5 3.0 2.5 2.0 1.5
f1 (ppm)

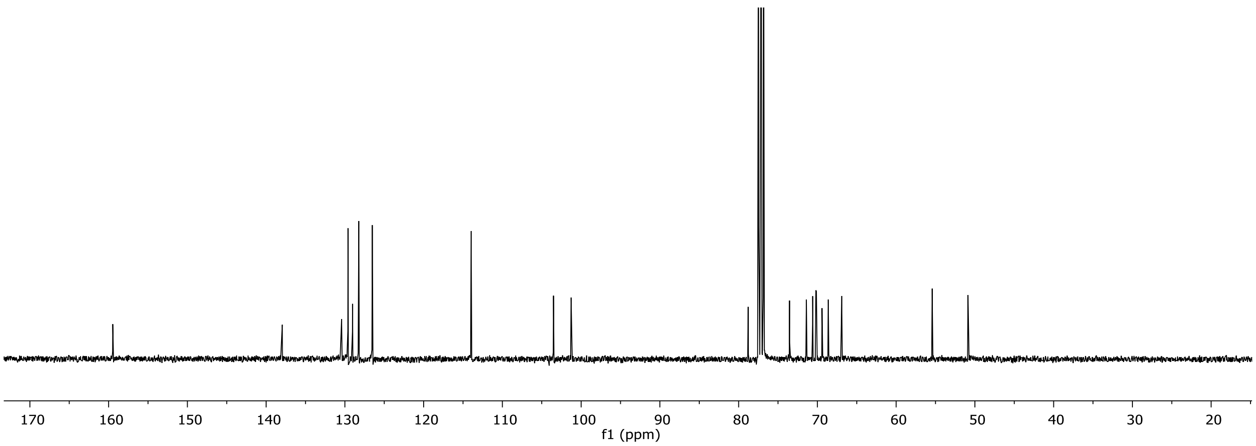
170 160 150 140 130 120 110 100 90 80 70 60 50 40 30 20
f1 (ppm)

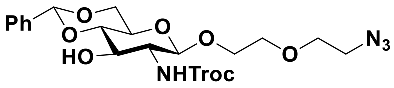
Ph
O
O
O
HO
NHTroc
O
O
N3

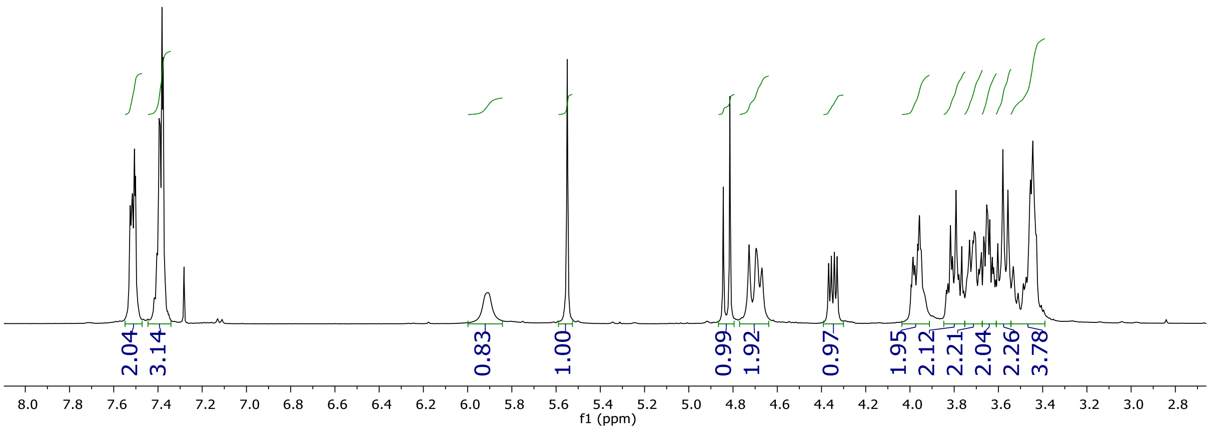
2.04
3.14
0.83
1.00
0.99
1.92
0.97
1.95
2.12
2.21
2.04
2.26
3.78
8.0 7.8 7.6 7.4 7.2 7.0 6.8 6.6 6.4 6.2 6.0 5.8 5.6 5.4 5.2 5.0 4.8 4.6 4.4 4.2 4.0 3.8 3.6 3.4 3.2 3.0 2.8
f1 (ppm)

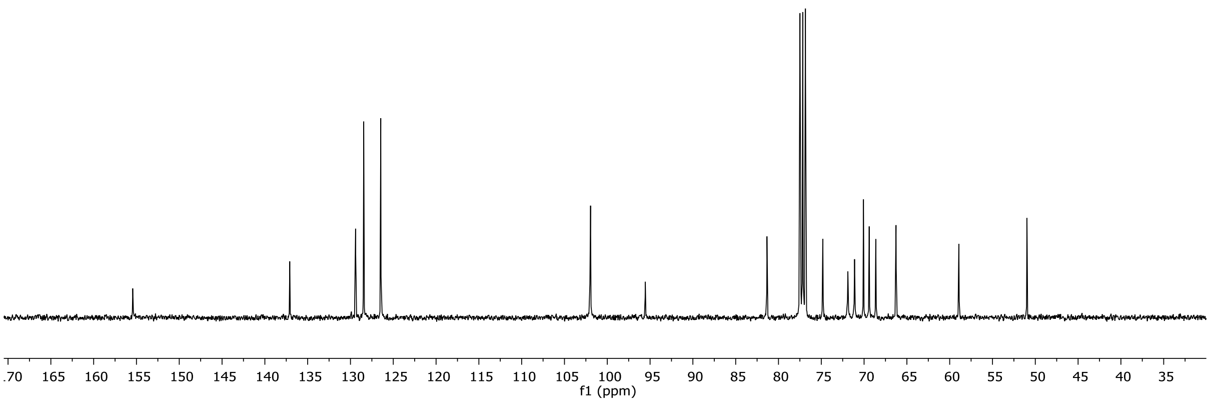
.70 165 160 155 150 145 140 135 130 125 120 115 110 105 100 95 90 85 80 75 70 65 60 55 50 45 40 35
f1 (ppm)

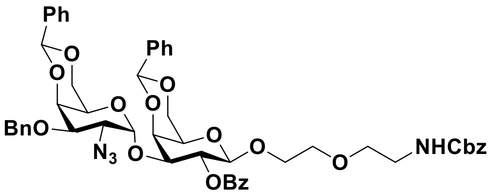
Ph
O
O
BnO
O
N3
O
Ph
O
O
O
O
O
O
NHCbz
OBz

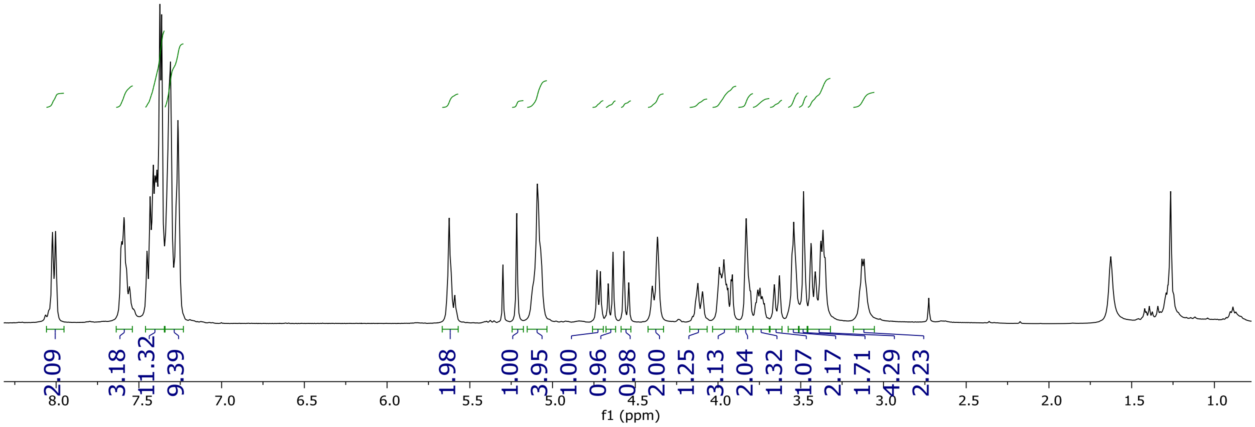
2.09
3.18
11.32
9.39
1.98
1.00
3.95
1.00
0.96
0.98
2.00
1.25
3.13
2.04
1.32
1.07
2.17
1.71
4.29
2.23
8.0
7.5
7.0
6.5
6.0
5.5
5.0
4.5
4.0
3.5
3.0
2.5
2.0
1.5
1.0
f1 (ppm)

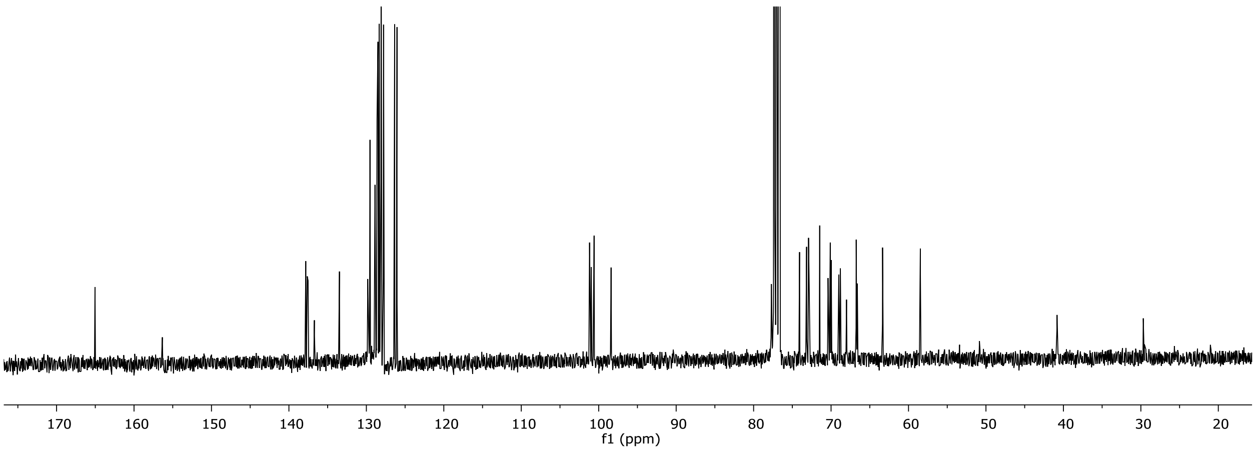
170
160
150
140
130
120
110
100
90
80
70
60
50
40
30
20
f1 (ppm)

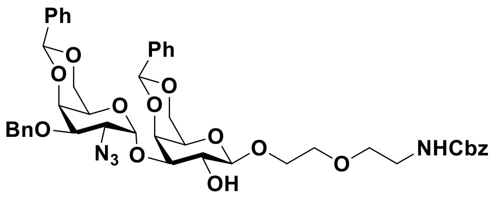
Ph
O
O
BnO
O
N3
O
Ph
O
O
O
O
OH
O
O
NHCbz

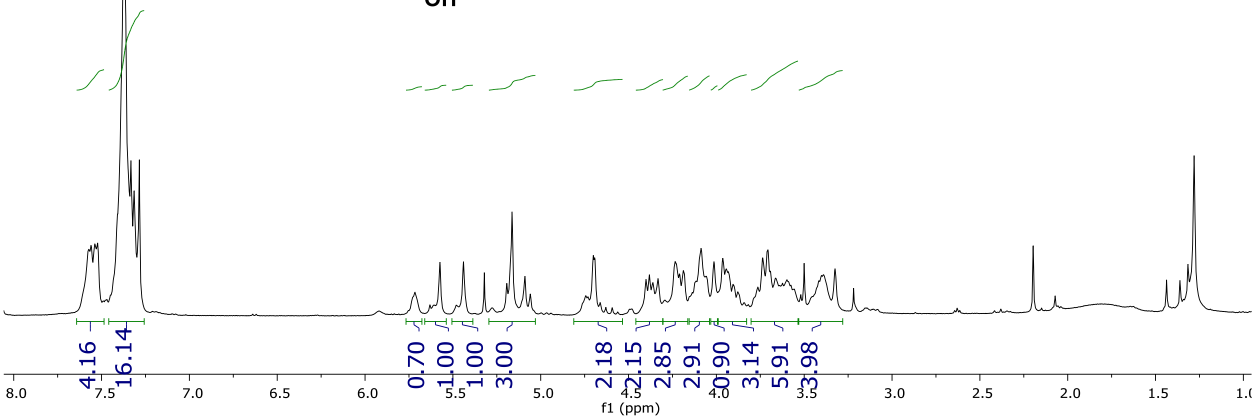
4.16
16.14
0.70
1.00
1.00
3.00
2.18
2.15
2.85
2.91
0.90
3.14
5.91
3.98
8.0 7.5 7.0 6.5 6.0 5.5 5.0 4.5 4.0 3.5 3.0 2.5 2.0 1.5
f1 (ppm)

.

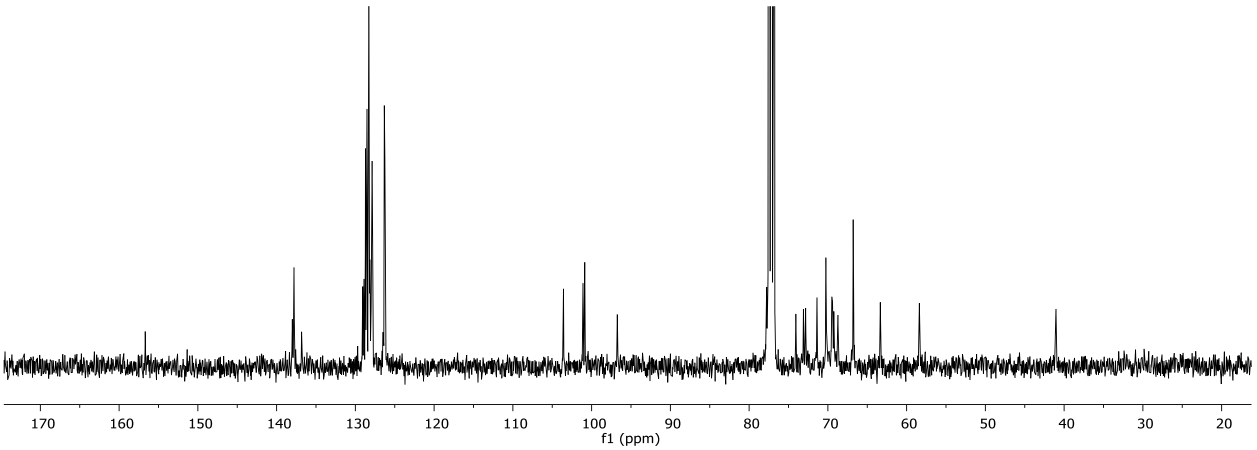
170 160 150 140 130 120 110 100 90 80 70 60 50 40 30 20
f1 (ppm)

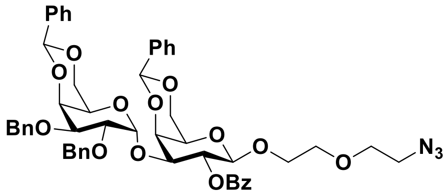
Ph
Ph
BnO
BnO
OBz
N_3

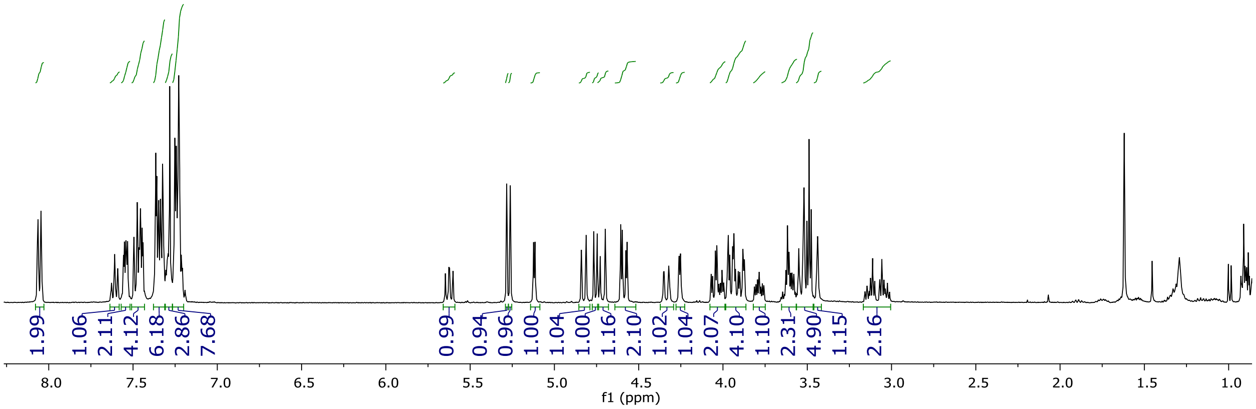
1.99
1.06
2.11
4.12
6.18
2.86
7.68
0.99
0.94
0.96
1.00
1.04
1.00
1.16
2.10
1.02
1.04
2.07
4.10
1.10
2.31
4.90
1.15
2.16
8.0 7.5 7.0 6.5 6.0 5.5 5.0 4.5 4.0 3.5 3.0 2.5 2.0 1.5 1.0
f1 (ppm)

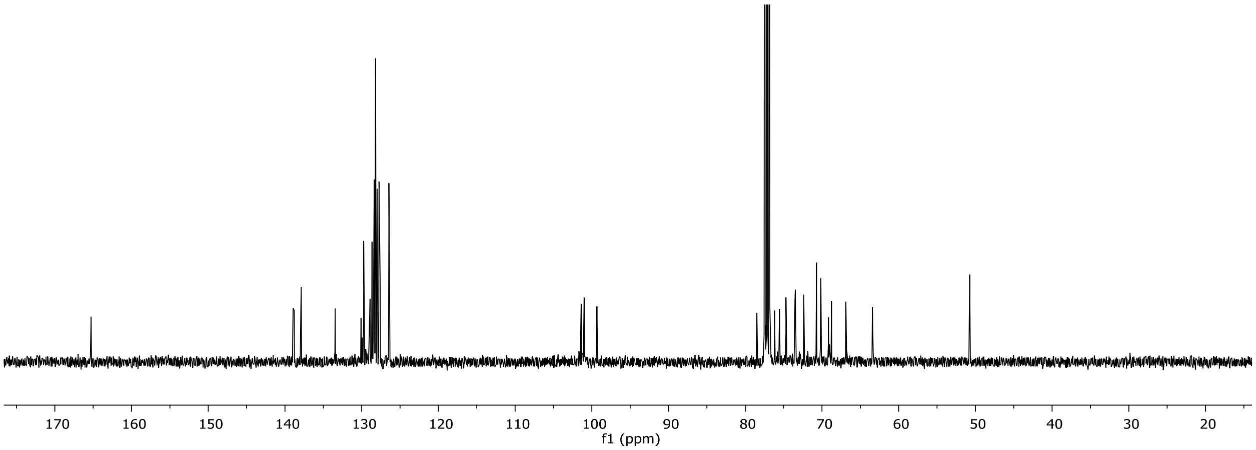
170 160 150 140 130 120 110 100 90 80 70 60 50 40 30 20
f1 (ppm)

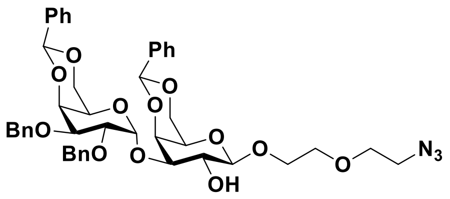
Ph
O
O
O
BnO
BnO
O
Ph
O
O
O
O
O
O
N3
OH

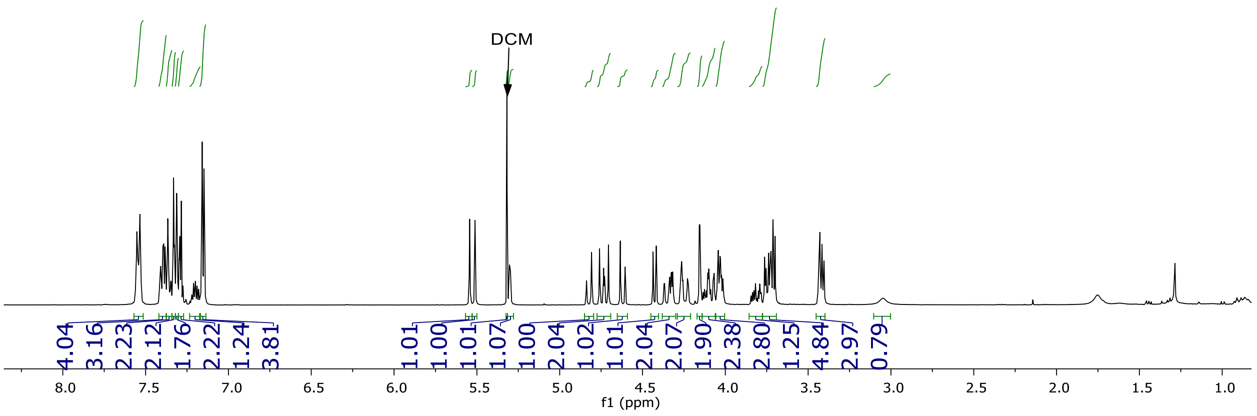
DCM
4.04
3.16
2.23
2.12
1.76
2.22
1.24
3.81
1.01
1.00
1.01
1.07
1.00
2.04
1.02
1.01
2.04
2.07
1.90
2.38
2.80
1.25
4.84
2.97
0.79
f1 (ppm)

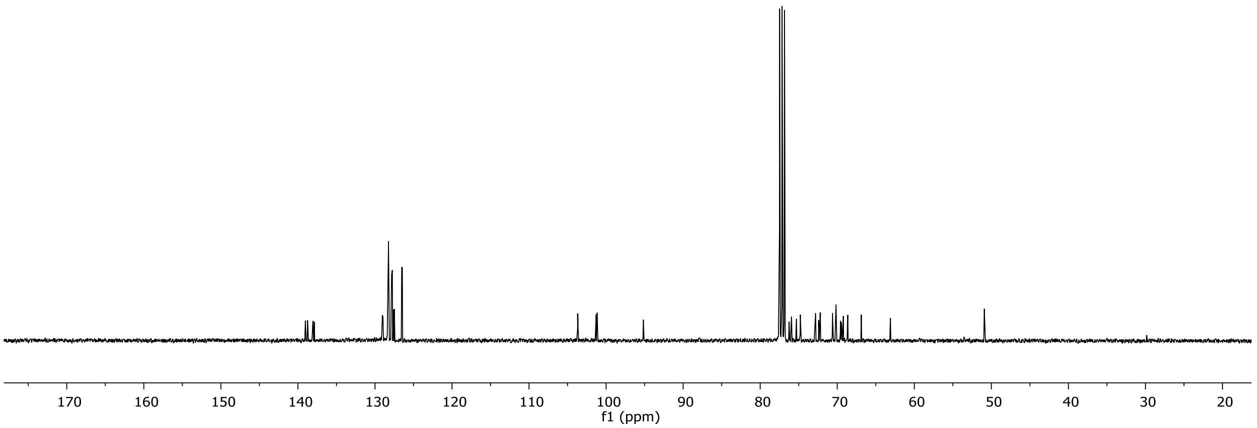
f1 (ppm)

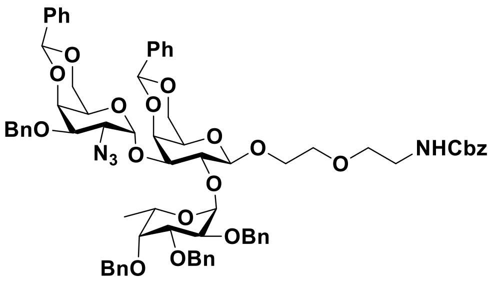
Ph
O
O
O
BnO
N3
O
O
O
Ph
O
O
O
O
NHCbz
O
O
OBn
OBn
BnO

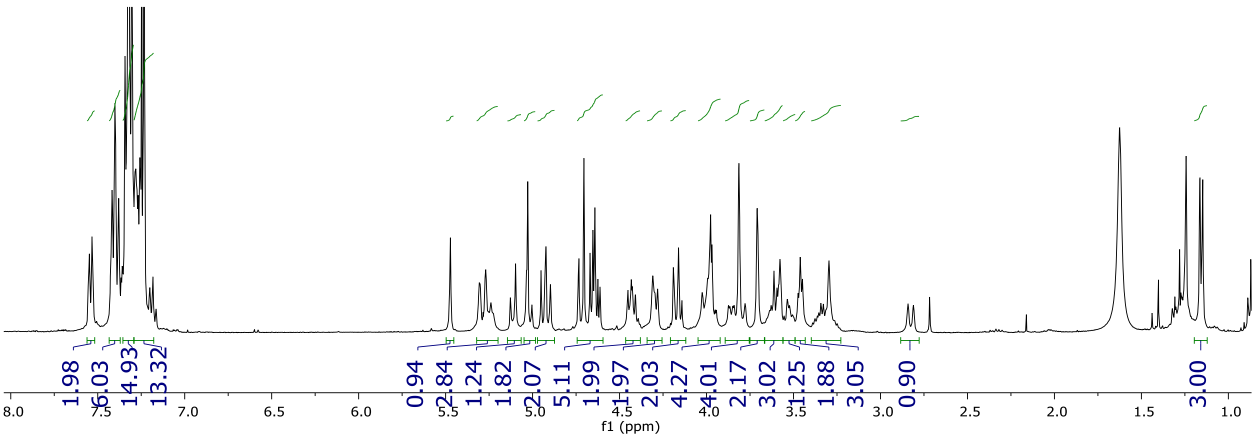
1.98
6.03
14.93
13.32
0.94
2.84
1.24
1.82
2.07
5.11
1.99
1.97
2.03
4.27
4.01
2.17
3.02
1.25
1.88
3.05
0.90
3.00
8.0
7.5
7.0
6.5
6.0
5.5
5.0
4.5
4.0
3.5
3.0
2.5
2.0
1.5
1.0
f1 (ppm)

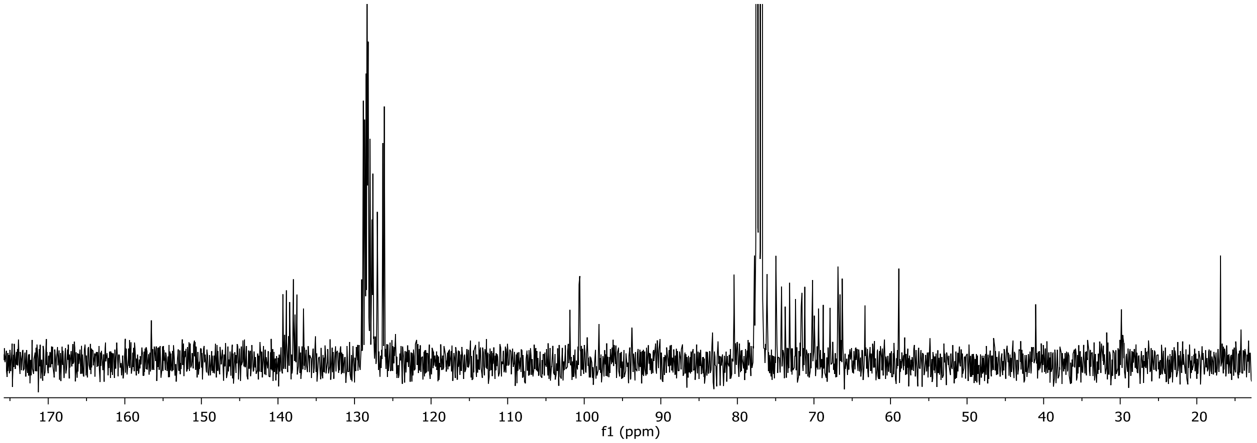
170
160
150
140
130
120
110
100
90
80
70
60
50
40
30
20
f1 (ppm)

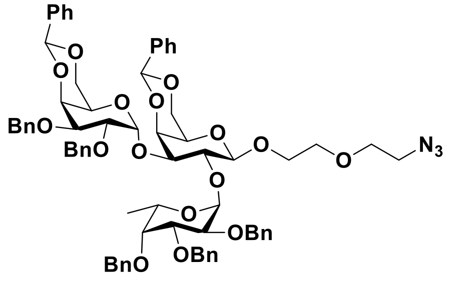

Ph
O
O
O
BnO
BnO
O
Ph
O
O
O
O
O
N3
O
O
OBn
BnO
OBn

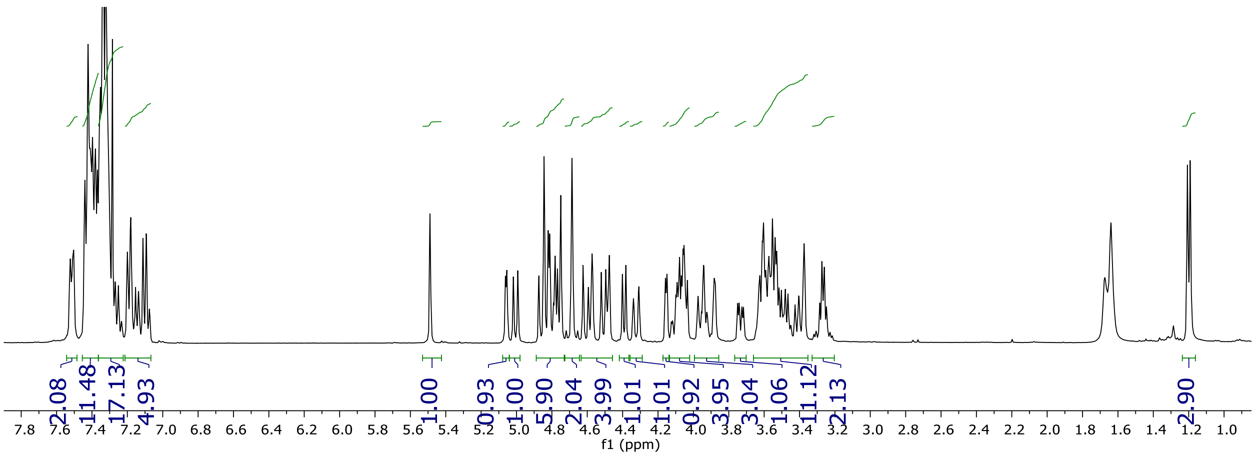

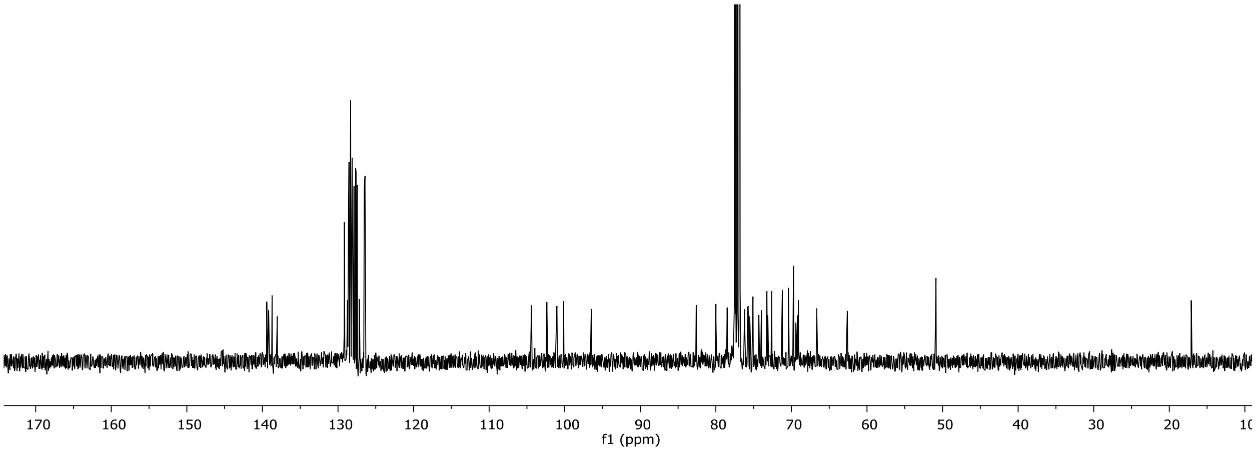

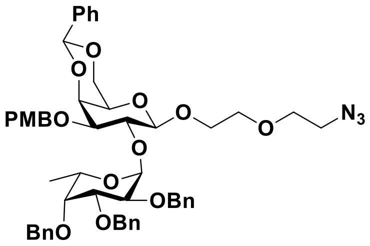

Ph
O
O
O
PMBO
O
O
N3
O
O
OBn
BnO
OBn

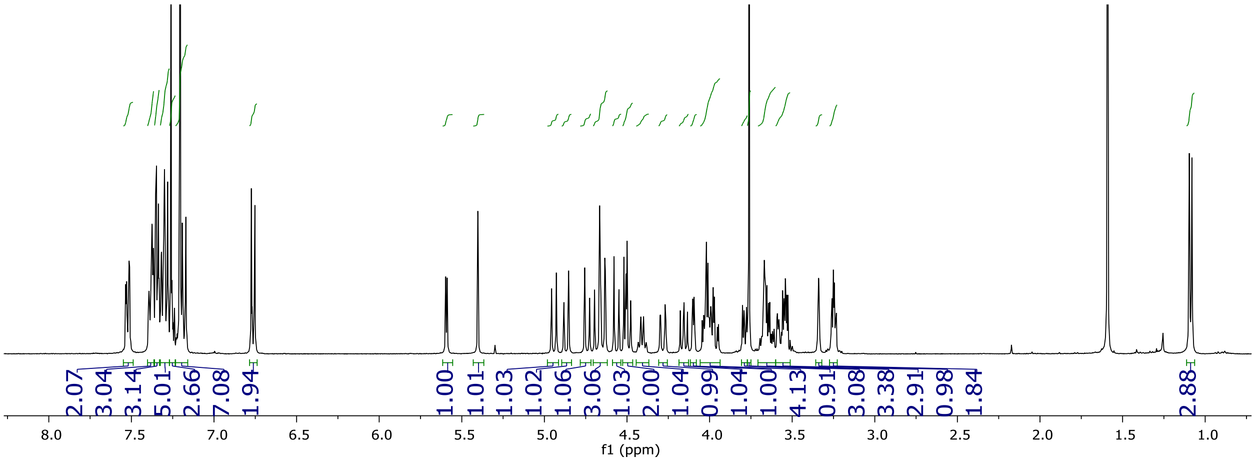

2.07
3.04
3.14
5.01
2.66
7.08
1.94
1.00
1.01
1.03
1.02
1.06
3.06
1.03
2.00
1.04
0.99
1.04
1.00
4.13
0.91
3.08
3.38
2.91
0.98
1.84
2.88
8.0
7.5
7.0
6.5
6.0
5.5
5.0
4.5
4.0
3.5
3.0
2.5
2.0
1.5
1.0
f1 (ppm)

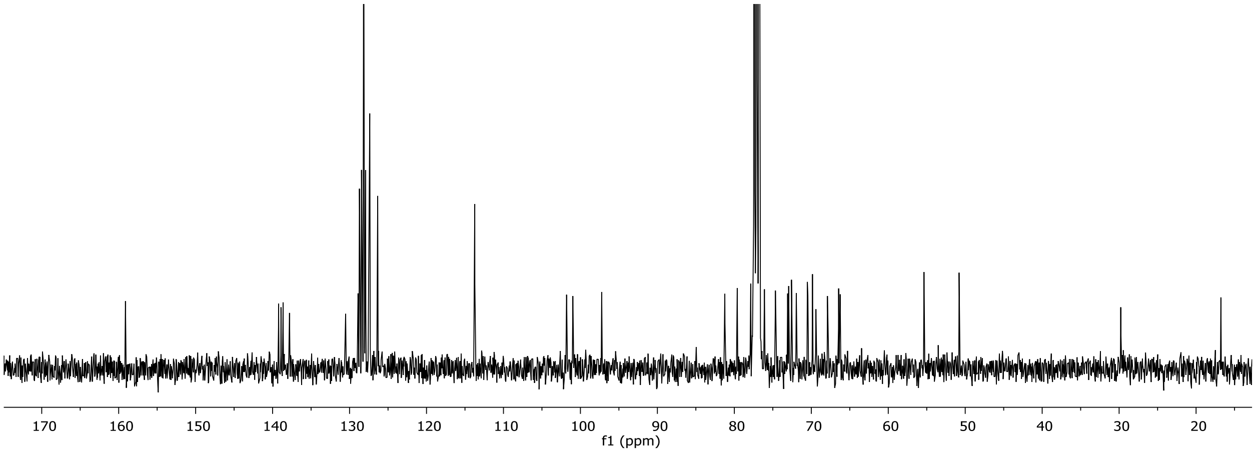

170
160
150
140
130
120
110
100
90
80
70
60
50
40
30
20
f1 (ppm)

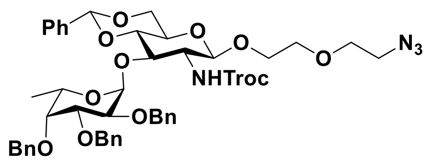
Ph
O
O
O
O
O
N3
NHTroc
OBn
OBn
BnO

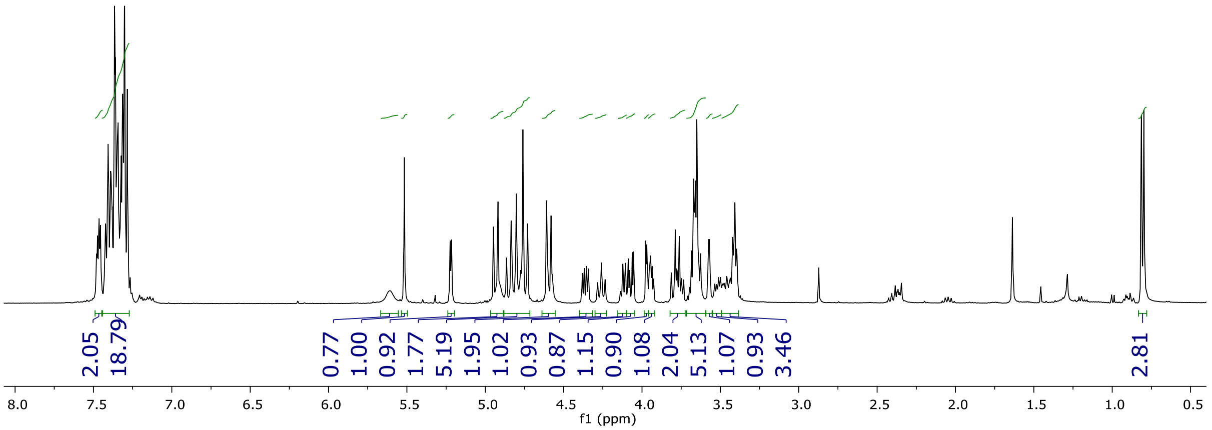
2.05
18.79
0.77
1.00
0.92
1.77
5.19
1.95
1.02
0.93
0.87
1.15
0.90
1.08
2.04
5.13
1.07
0.93
3.46
2.81
8.0 7.5 7.0 6.5 6.0 5.5 5.0 4.5 4.0 3.5 3.0 2.5 2.0 1.5 1.0 0.5
f1 (ppm)

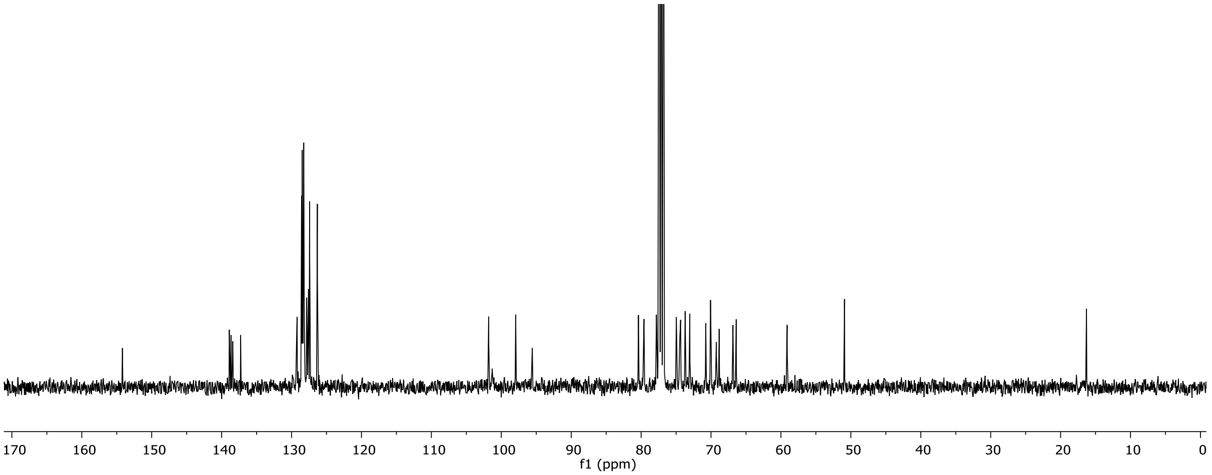
170 160 150 140 130 120 110 100 90 80 70 60 50 40 30 20 10 0
f1 (ppm)

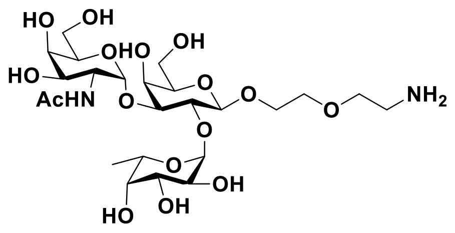
HO
OH
OHO
OH
HO
AcHN
O
O
O
NH2
O
OH
HO
OH

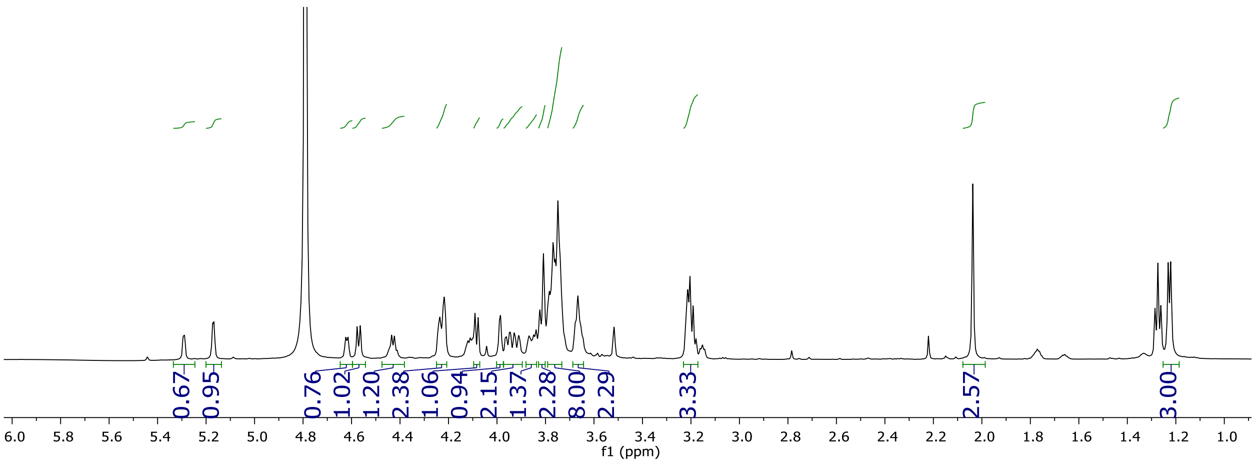
0.67
0.95
0.76
1.02
1.20
2.38
1.06
0.94
2.15
1.37
2.28
8.00
2.29
3.33
2.57
3.00
6.0 5.8 5.6 5.4 5.2 5.0 4.8 4.6 4.4 4.2 4.0 3.8 3.6 3.4 3.2 3.0 2.8 2.6 2.4 2.2 2.0 1.8 1.6 1.4 1.2 1.0
f1 (ppm)

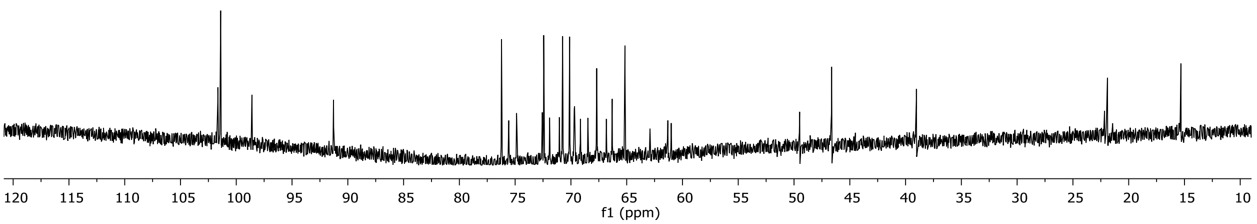
120 115 110 105 100 95 90 85 80 75 70 65 60 55 50 45 40 35 30 25 20 15 10
f1 (ppm)

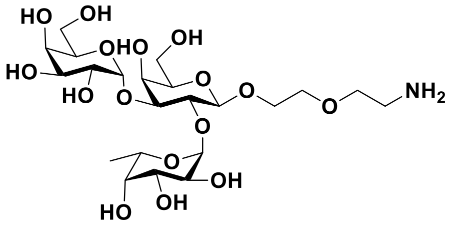
HO
OH
OHO
OH
O
HO
O
HO
O
O
NH2
O
O
OH
HO
OH

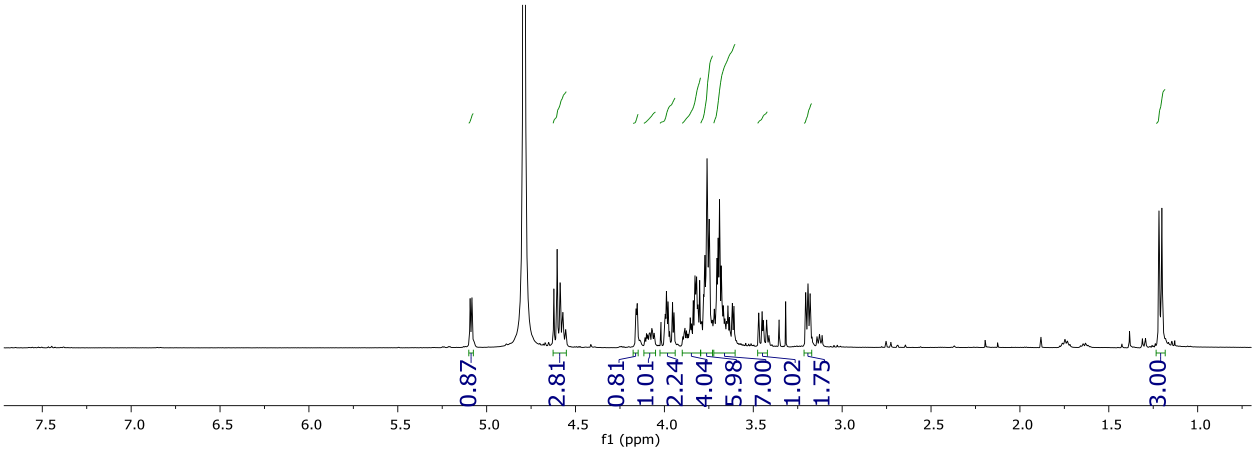
0.87
2.81
0.81
1.01
2.24
4.04
5.98
7.00
1.02
1.75
3.00
7.5
7.0
6.5
6.0
5.5
5.0
4.5
4.0
3.5
3.0
2.5
2.0
1.5
1.0
f1 (ppm)

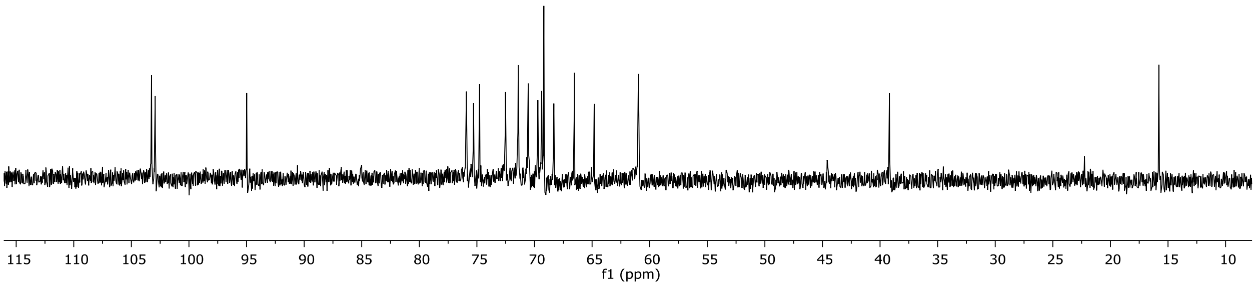
115
110
105
100
95
90
85
80
75
70
65
60
55
50
45
40
35
30
25
20
15
10
f1 (ppm)

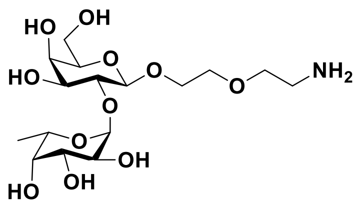
HO
OH
O
O
O
NH2
HO
O
O
OH
HO
OH

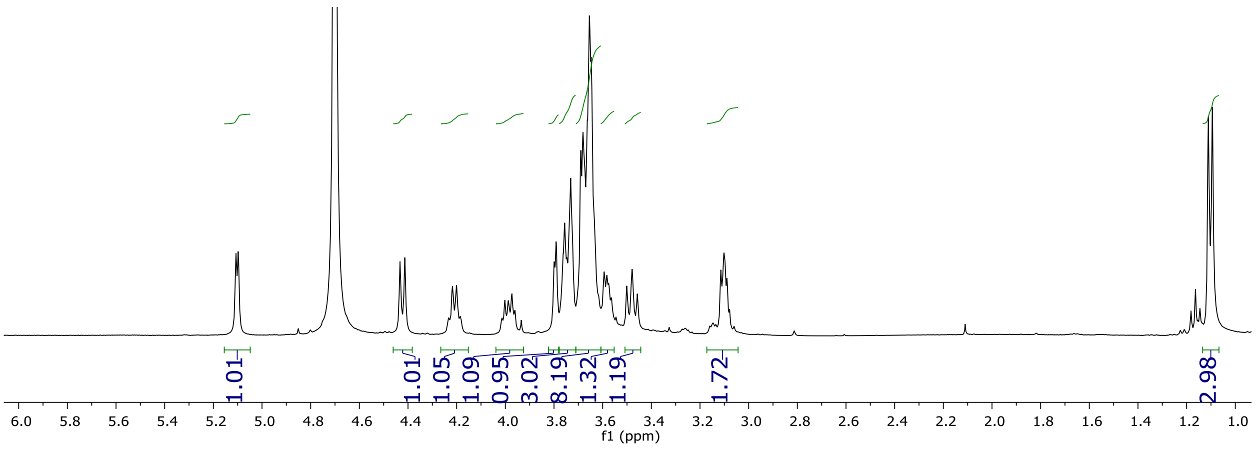
1.01
1.01
1.05
1.09
0.95
3.02
8.19
1.32
1.19
1.72
2.98
6.0 5.8 5.6 5.4 5.2 5.0 4.8 4.6 4.4 4.2 4.0 3.8 3.6 3.4 3.2 3.0 2.8 2.6 2.4 2.2 2.0 1.8 1.6 1.4 1.2 1.0
f1 (ppm)

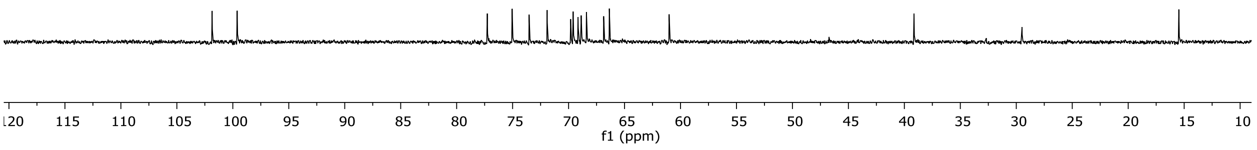
120 115 110 105 100 95 90 85 80 75 70 65 60 55 50 45 40 35 30 25 20 15 10
f1 (ppm)

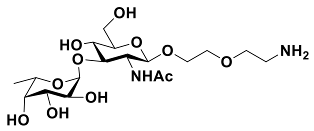
OH
HO
O
O
O
O
NH2
NHAc
O
OH
HO
OH

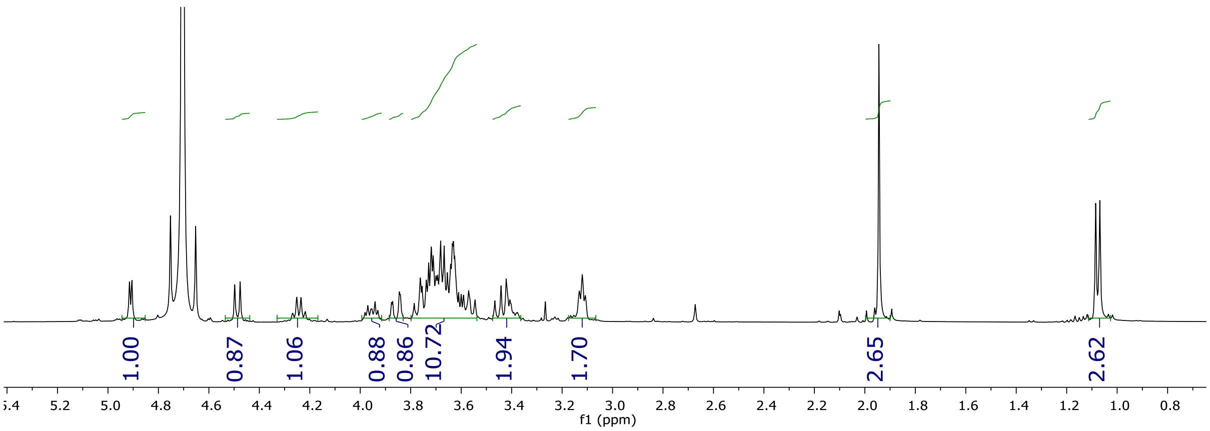
1.00
0.87
1.06
0.88
0.86
10.72
1.94
1.70
2.65
2.62
5.2 5.0 4.8 4.6 4.4 4.2 4.0 3.8 3.6 3.4 3.2 3.0 2.8 2.6 2.4 2.2 2.0 1.8 1.6 1.4 1.2 1.0 0.8
f1 (ppm)

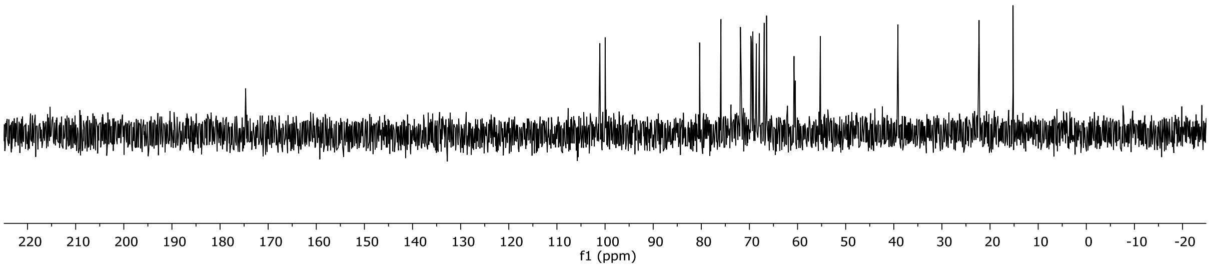
220 210 200 190 180 170 160 150 140 130 120 110 100 90 80 70 60 50 40 30 20 10 0 -10 -20
f1 (ppm)

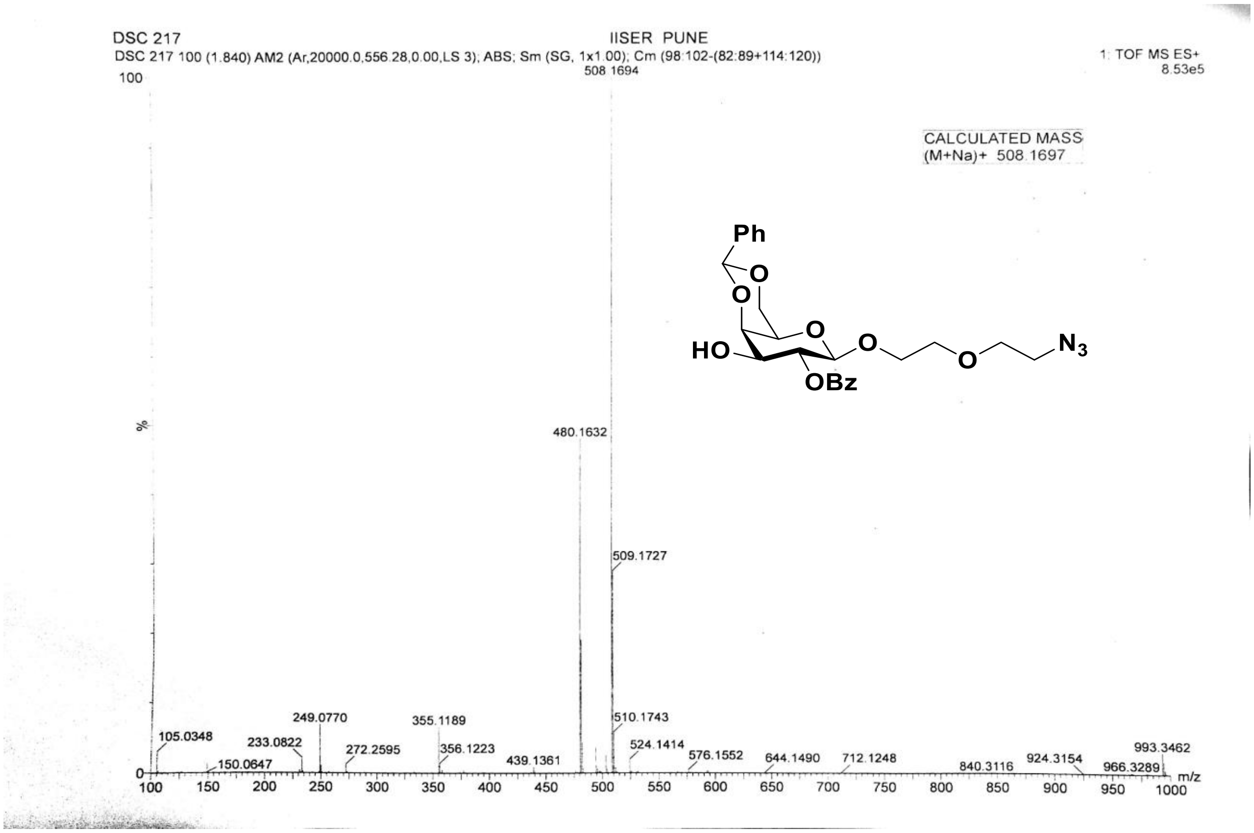
DSC 217
IISER PUNE
DSC 217 100 (1.840) AM2 (Ar,20000.0,556.28,0.00,LS 3); ABS; Sm (SG, 1x1.00); Cm (98:102-(82:89+114:120))
1: TOF MS ES+
8.53e5
CALCULATED MASS
(M+Na)+ 508.1697
Ph
O
O
O
HO
OBz
O
O
N3
508.1694
480.1632
509.1727
510.1743
524.1414
576.1552
644.1490
712.1248
840.3116
924.3154
966.3289
993.3462
105.0348
150.0647
233.0822
249.0770
272.2595
355.1189
356.1223
439.1361
100
%
0
m/z

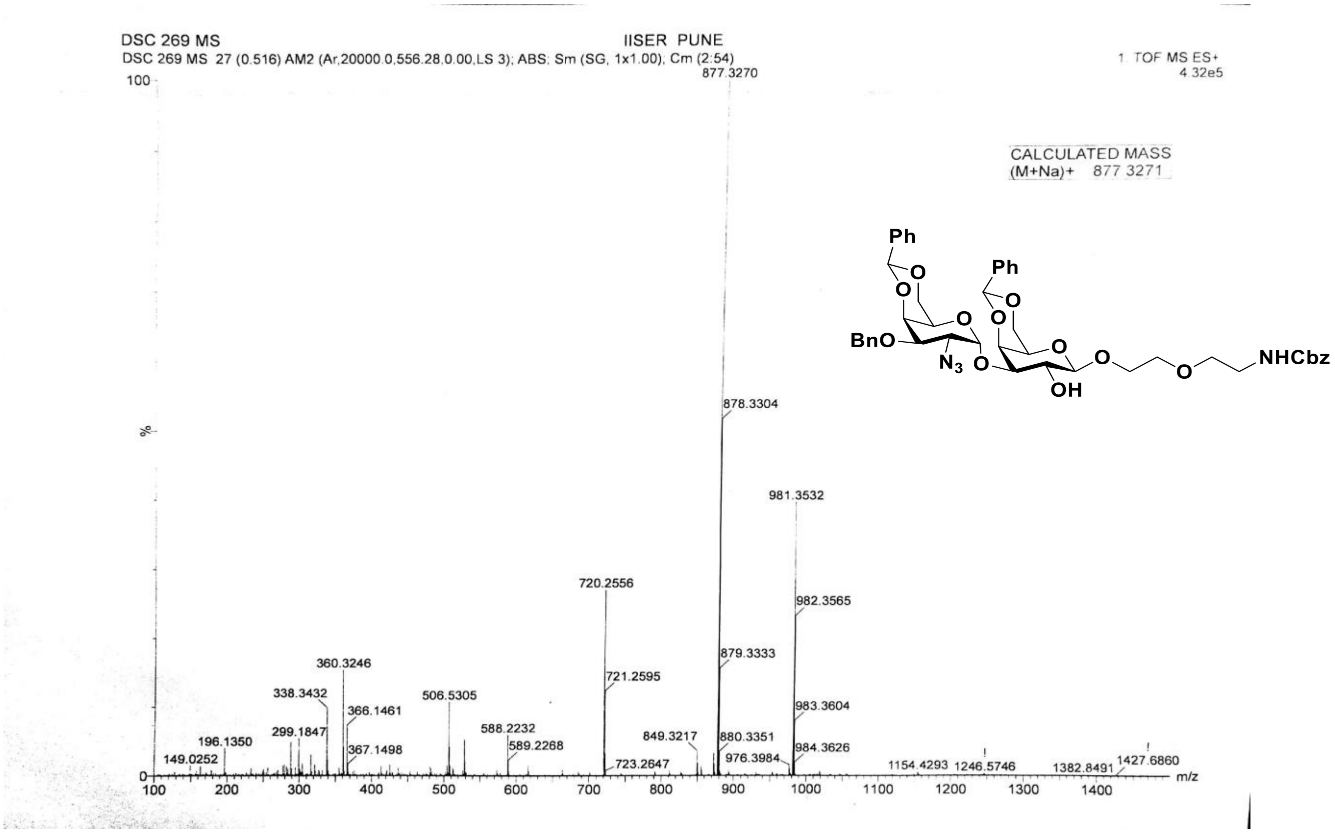
DSC 269 MS
IISER PUNE
DSC 269 MS 27 (0.516) AM2 (Ar,20000.0,556.28,0.00,LS 3); ABS; Sm (SG, 1x1.00); Cm (2:54)
1: TOF MS ES+
4.32e5
CALCULATED MASS
(M+Na)+ 877.3271
Ph
O
O
BnO
N3
OH
NHCbz
877.3270
878.3304
981.3532
982.3565
720.2556
879.3333
721.2595
360.3246
338.3432
366.1461
506.5305
983.3604
588.2232
299.1847
849.3217
880.3351
196.1350
367.1498
589.2268
984.3626
149.0252
723.2647
976.3984
1154.4293
1246.5746
1382.8491
1427.6860
%
m/z

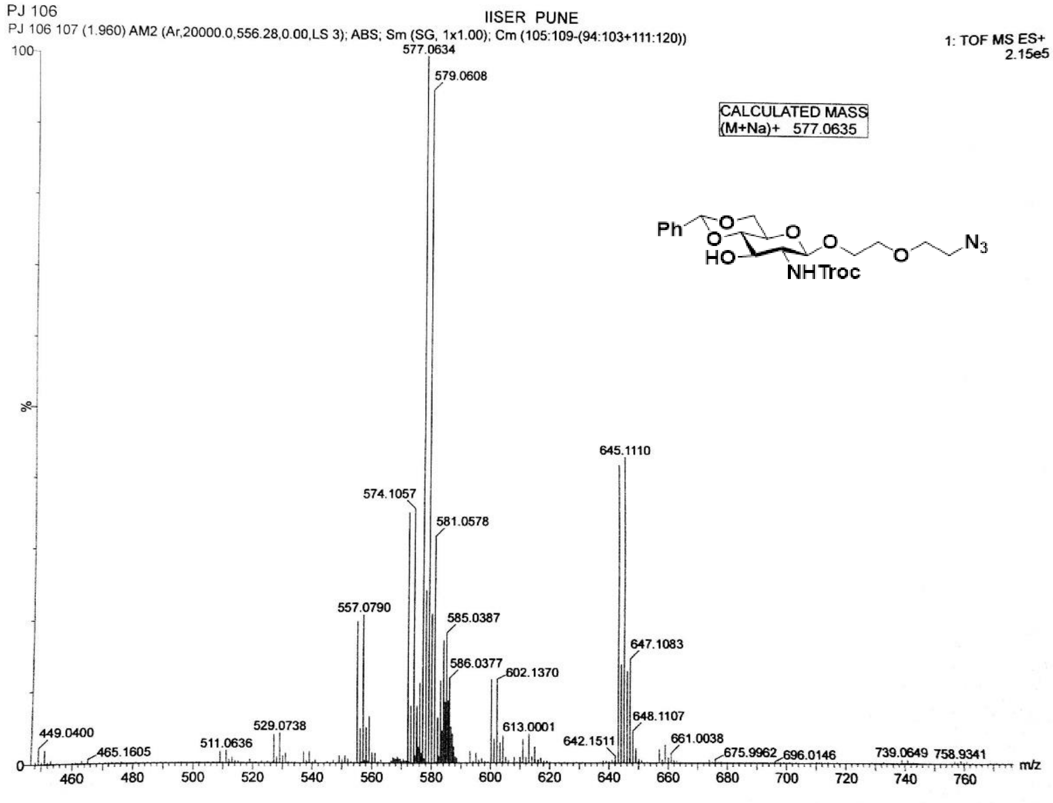
PJ 106
IISER PUNE
PJ 106 107 (1.960) AM2 (Ar,20000.0,556.28,0.00,LS 3); ABS; Sm (SG, 1x1.00); Cm (105:109-(94:103+111:120))
1: TOF MS ES+
2.15e5
CALCULATED MASS
(M+Na)+ 577.0635
Ph
O
O
HO
NHTroc
N3
100
%
0
577.0634
579.0608
645.1110
574.1057
581.0578
557.0790
585.0387
647.1083
586.0377
602.1370
648.1107
613.0001
449.0400
529.0738
511.0636
642.1511
661.0038
465.1605
675.9962
696.0146
739.0649
758.9341
460
480
500
520
540
560
580
600
620
640
660
680
700
720
740
760
m/z

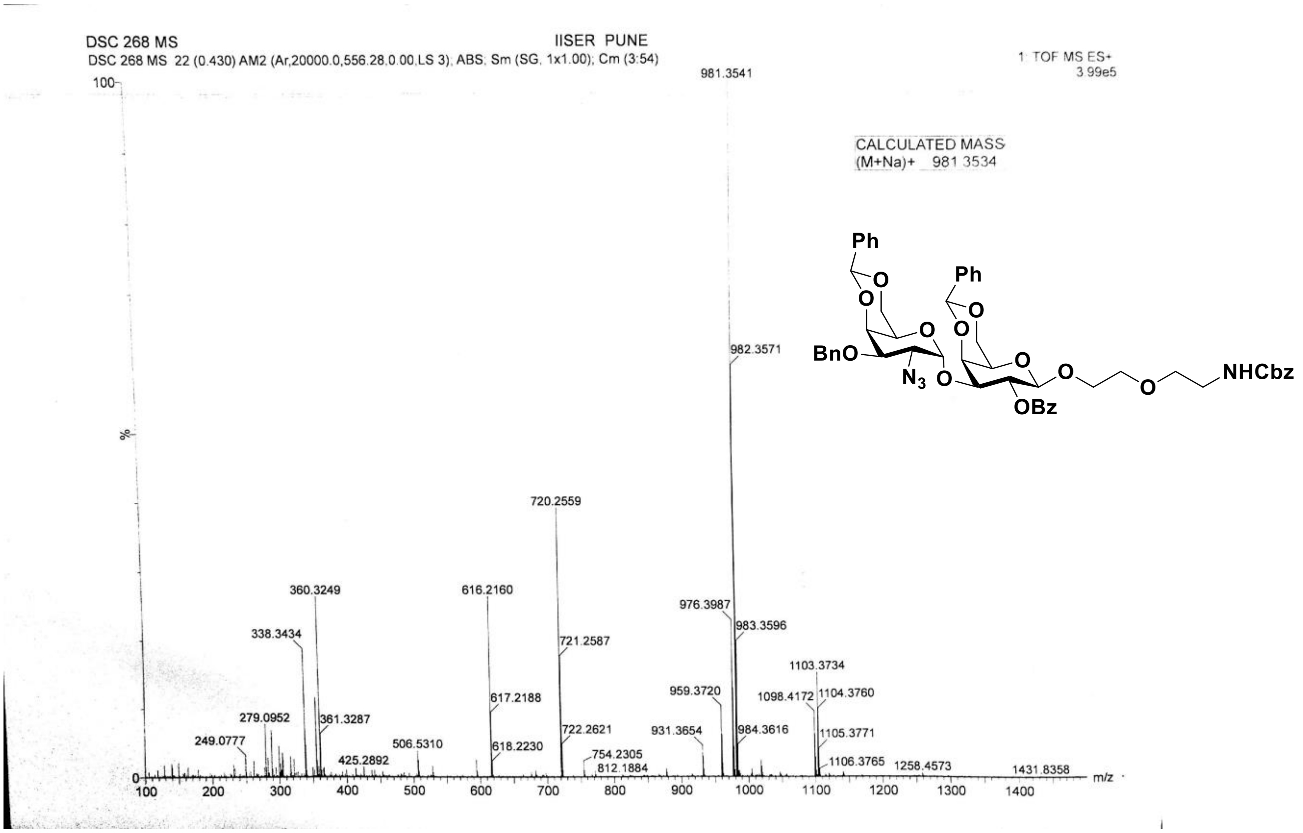
DSC 268 MS
IISER PUNE
DSC 268 MS 22 (0.430) AM2 (Ar,20000.0,556.28,0.00,LS 3); ABS; Sm (SG, 1x1.00); Cm (3:54)
1: TOF MS ES+
3.99e5
CALCULATED MASS
(M+Na)+ 981.3534
981.3541
982.3571
720.2559
616.2160
360.3249
338.3434
976.3987
983.3596
721.2587
1103.3734
959.3720
1098.4172
1104.3760
617.2188
279.0952
361.3287
722.2621
931.3654
984.3616
1105.3771
249.0777
506.5310
754.2305
425.2892
618.2230
812.1884
1106.3765
1258.4573
1431.8358
m/z
%
Ph
BnO
N3
OBz
NHCbz

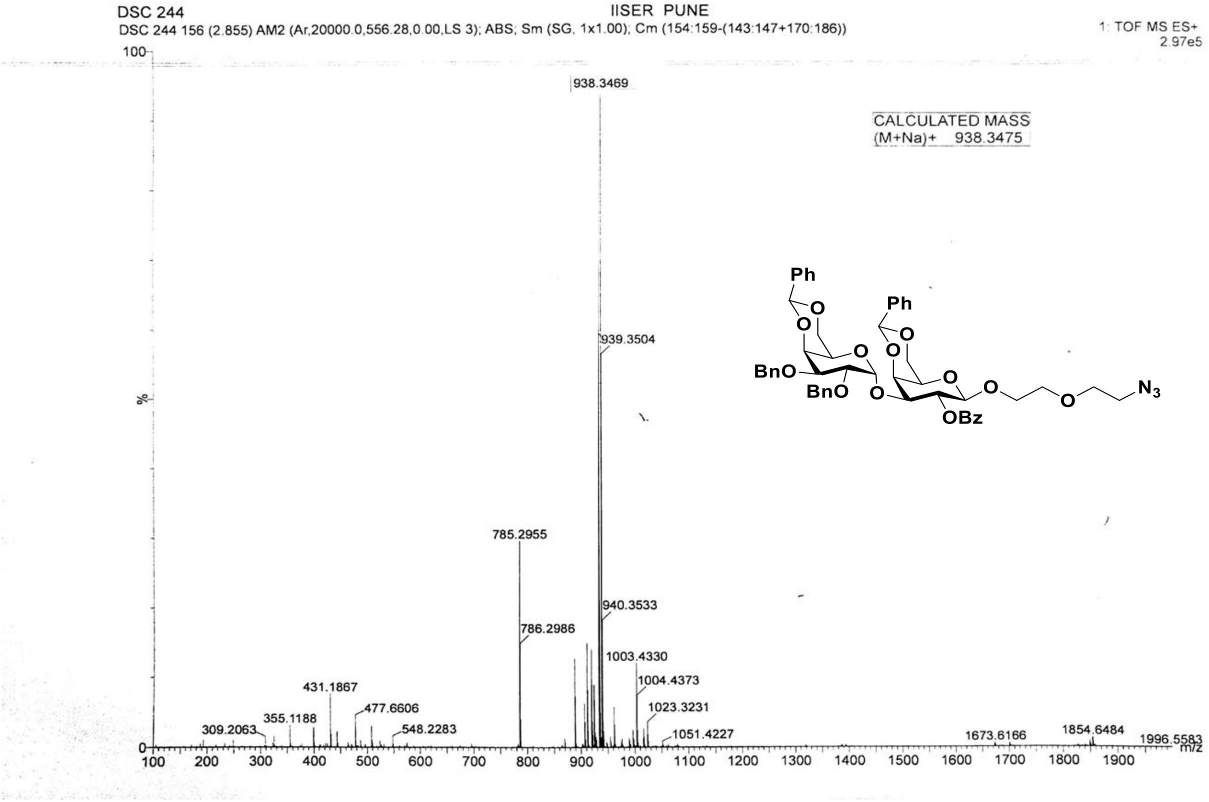
DSC 244
IISER PUNE
DSC 244 156 (2.855) AM2 (Ar,20000.0,556.28,0.00,LS 3); ABS; Sm (SG, 1x1.00); Cm (154:159-(143:147+170:186))
1: TOF MS ES+
2.97e5
938.3469
CALCULATED MASS
(M+Na)+ 938.3475
939.3504
785.2955
940.3533
786.2986
1003.4330
1004.4373
1023.3231
1051.4227
431.1867
477.6606
355.1188
548.2283
309.2063
1673.6166
1854.6484
1996.5583
m/z
%
Ph
O
BnO
BnO
OBz
N3

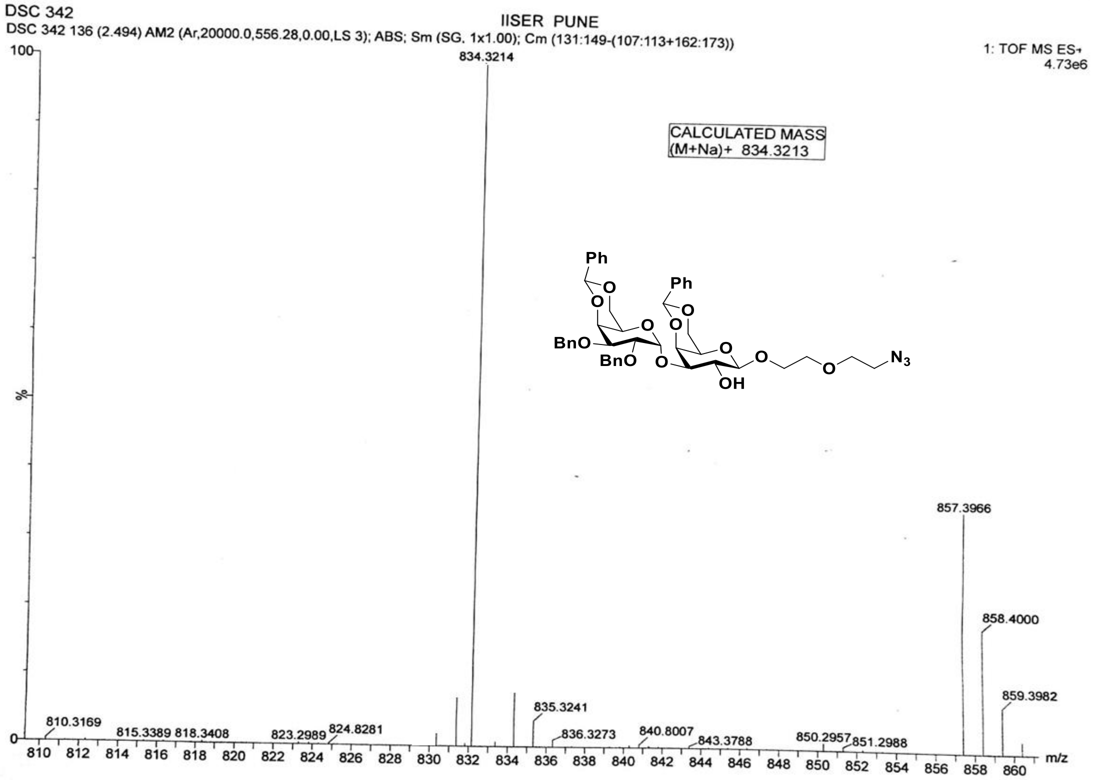
DSC 342
DSC 342 136 (2.494) AM2 (Ar,20000.0,556.28,0.00,LS 3); ABS; Sm (SG, 1x1.00); Cm (131:149-(107:113+162:173))
IISER PUNE
1: TOF MS ES+
4.73e6
834.3214
CALCULATED MASS
(M+Na)+ 834.3213
Ph
O
BnO
OH
N3
857.3966
858.4000
859.3982
810.3169
815.3389 818.3408
823.2989 824.8281
835.3241
836.3273
840.8007
843.3788
850.2957 851.2988
%
100
0
m/z

PJ 107

IISER PUNE

PJ 107 185 (3.390) AM2 (Ar,20000.0,556.28,0.00,LS 3); ABS; Sm (SG, 1x1.00); Cm (184:187-(86:178+192:265))

1: TOF MS ES+
2.41e5

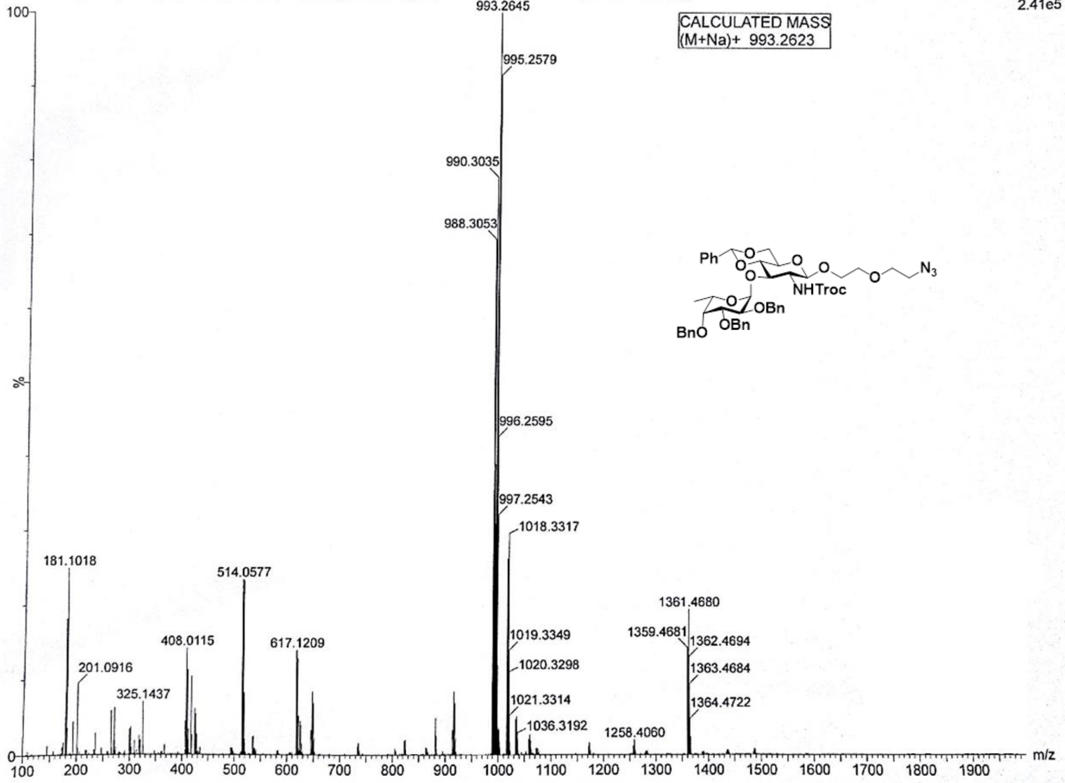

DSC 229 MS IISER PUNE

DSC 229 MS 40 (0.757) AM2 (Ar,20000.0,556.28,0.00,LS 3); ABS; Sm (SG, 1x1.00); Cm (2:54)

1 TOF MS ES+
1.84e5

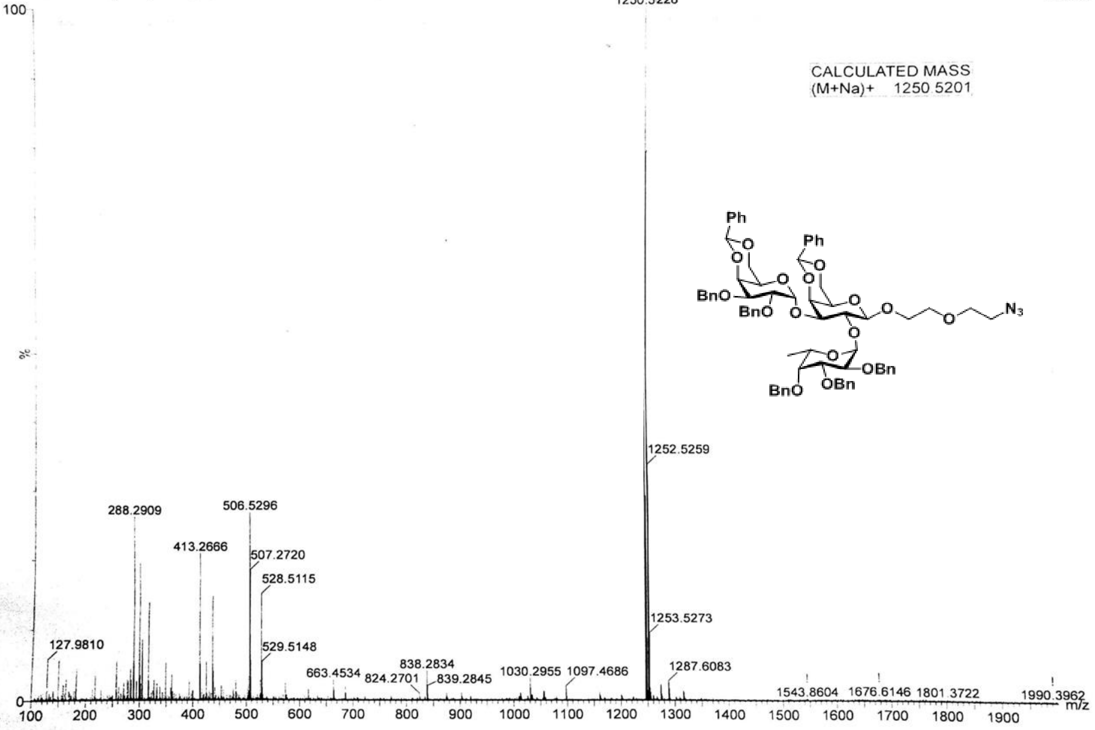

DSC 260 IISER PUNE

DSC 260 19 (0.379) AM2 (Ar,20000.0,556.28,0.00,LS 3); ABS; Sm (SG, 1x1.00); Cm (15:20-(1:12+28:45)) 1: TOF MS ES+ 4.13e4

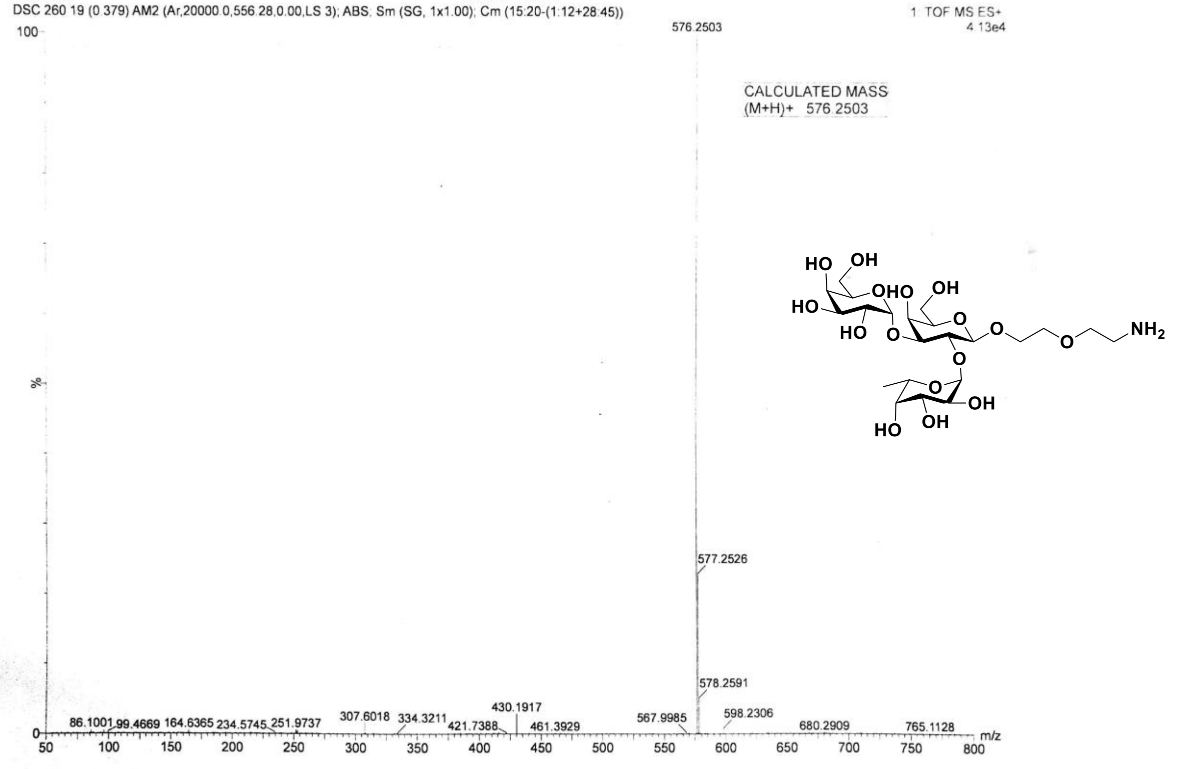

PJ 127 F

IISER PUNE

PJ 127 F 18 (0.342) AM2 (Ar,20000.0,556.28,0.00,LS 3); ABS; Sm (SG, 1x1.00); Cm (17:18-(2:15+19:270))

1: TOF MS ES+
1.01e5

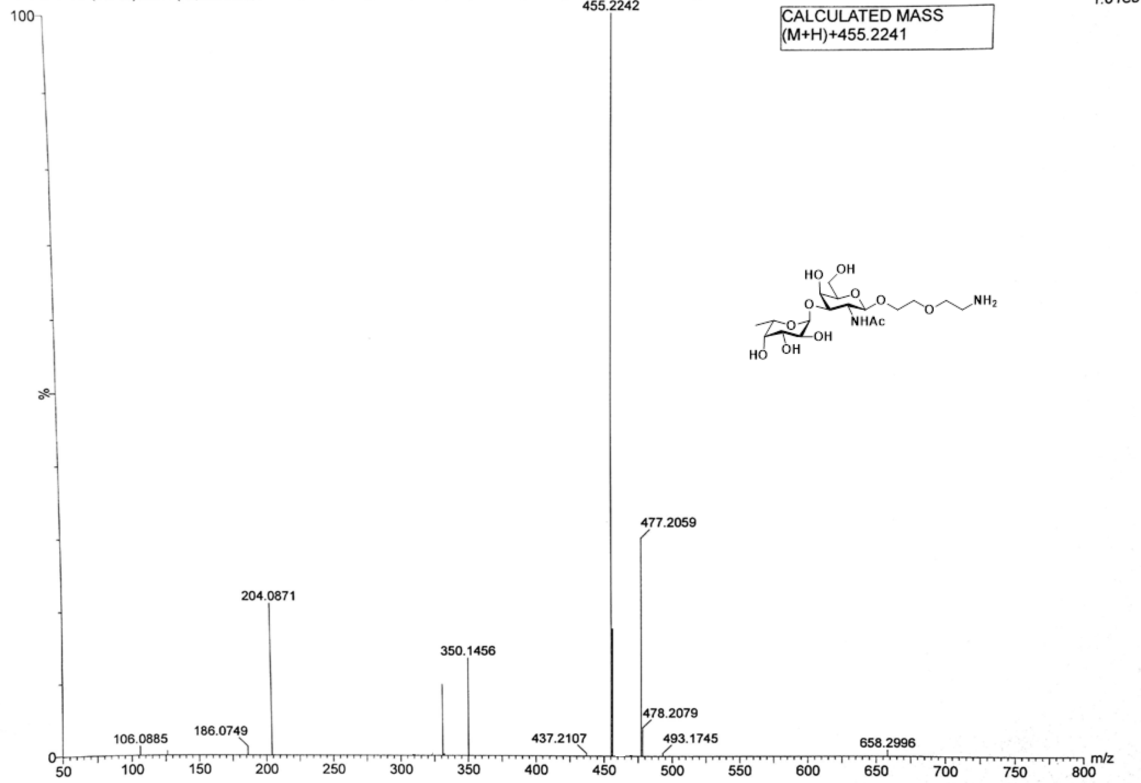

DSC 155 IISER PUNE

DSC 155 135 (2.477) AM2 (Ar,20000.0,556.28,0.00,LS 3); ABS; Sm (SG, 1x1.00); Cm (134:139-(2:133+140:273)) 1: TOF MS ES+ 9.70e5

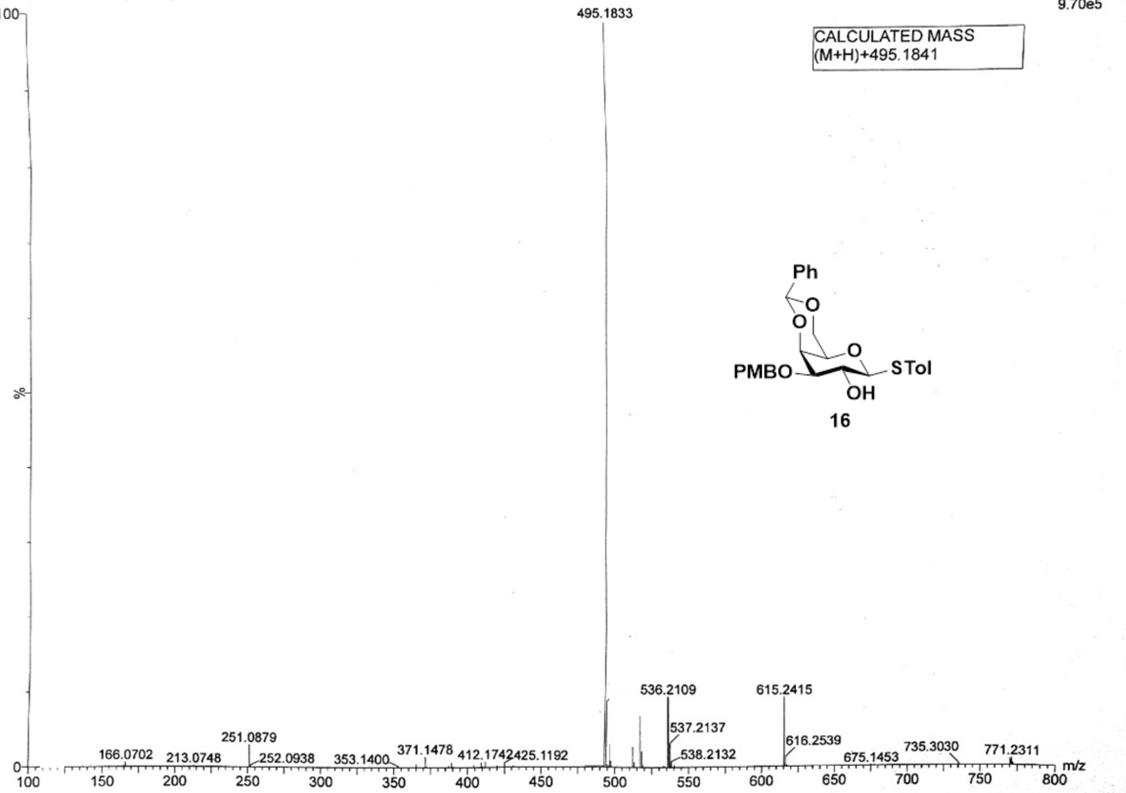

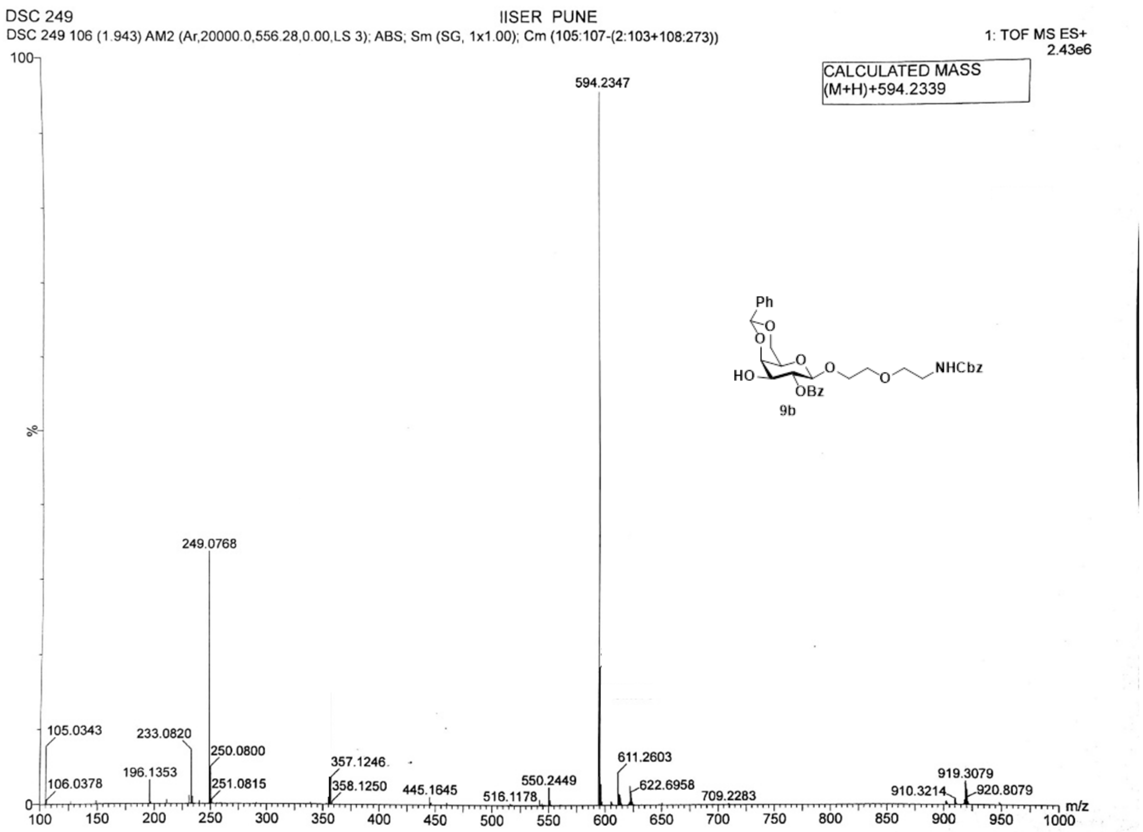
DSC 249
IISER PUNE
DSC 249 106 (1.943) AM2 (Ar,20000.0,556.28,0.00,LS 3); ABS; Sm (SG, 1x1.00); Cm (105:107-(2:103+108:273))
1: TOF MS ES+
2.43e6
CALCULATED MASS
(M+H)+594.2339
594.2347
249.0768
105.0343
106.0378
233.0820
196.1353
250.0800
251.0815
357.1246
358.1250
445.1645
516.1178
550.2449
611.2603
622.6958
709.2283
910.3214
919.3079
920.8079
%
m/z
Ph
HO
OBz
NHCbz
9b

DSC 217 IISER PUNE

DSC 217 105 (1.926) AM2 (Ar,20000.0,556.28,0.00,LS 3); ABS; Sm (SG, 1x1.00); Cm (104:106)

1: TOF MS ES+ 2.52e5

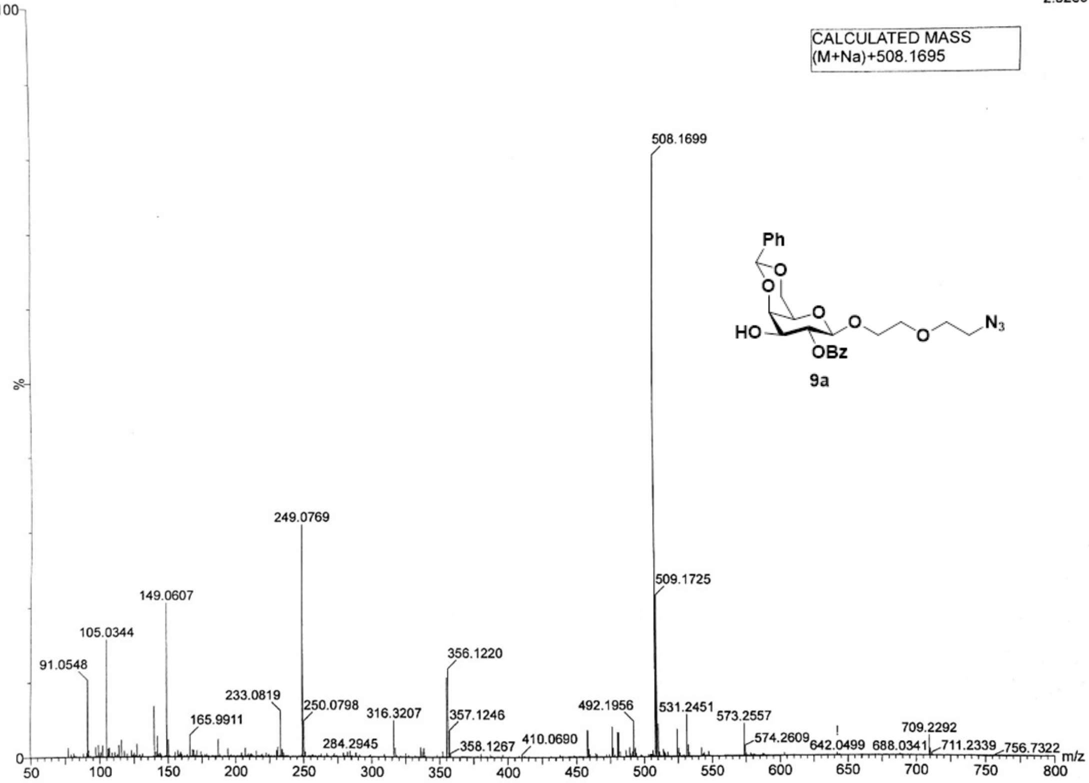

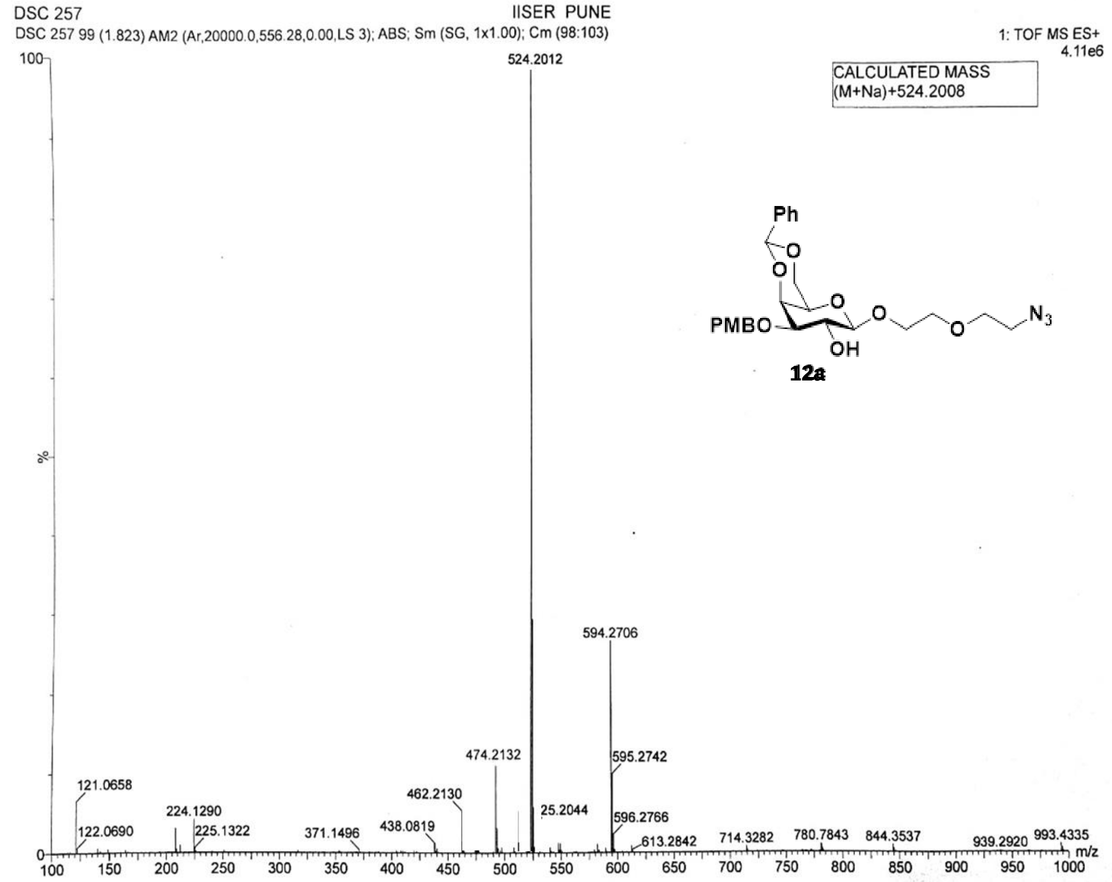
DSC 257
IISER PUNE
DSC 257 99 (1.823) AM2 (Ar,20000.0,556.28,0.00,LS 3); ABS; Sm (SG, 1x1.00); Cm (98:103)
1: TOF MS ES+
4.11e6
CALCULATED MASS
(M+Na)+524.2008
Ph
PMBO
OH
N3
12a
524.2012
594.2706
595.2742
596.2766
474.2132
462.2130
438.0819
371.1496
121.0658
122.0690
224.1290
225.1322
25.2044
613.2842
714.3282
780.7843
844.3537
939.2920
993.4335
m/z
%

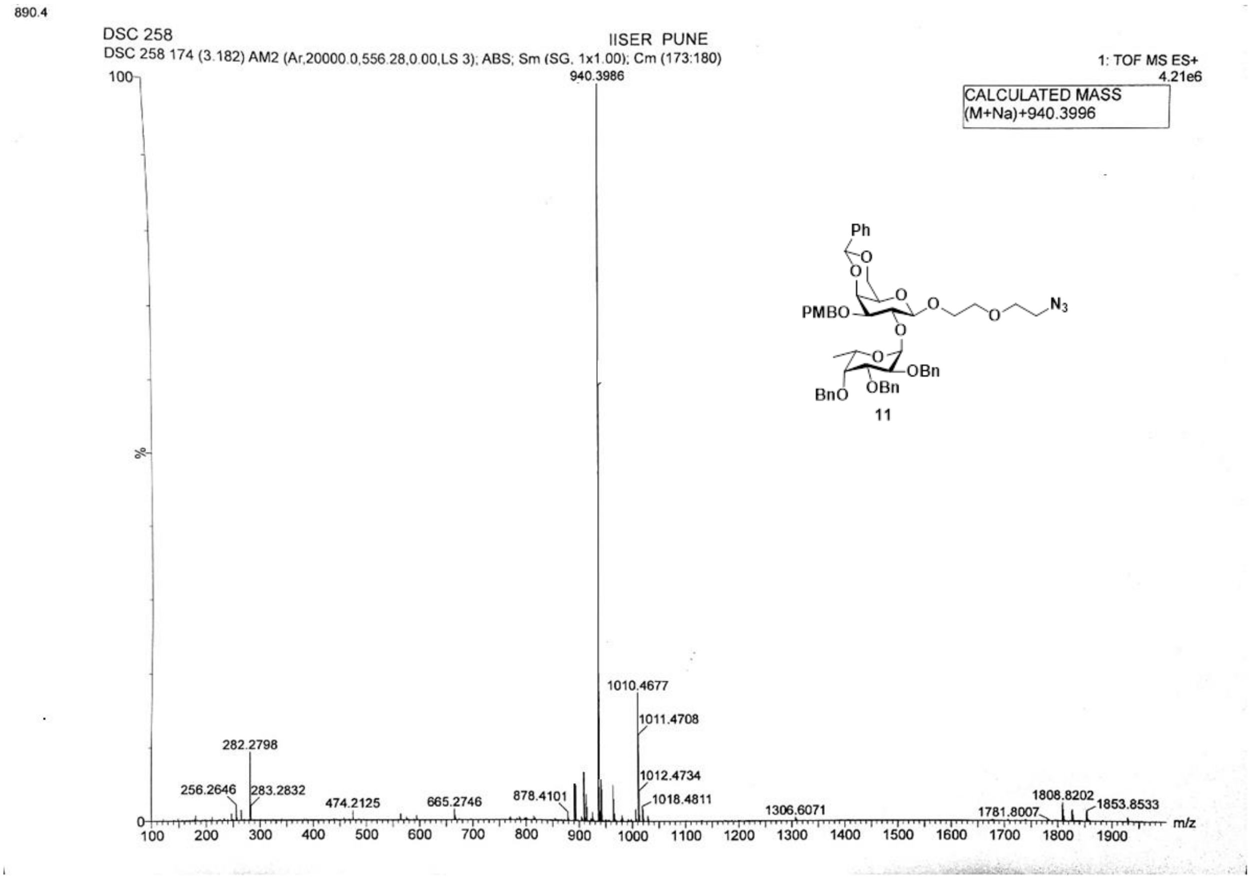
890.4
DSC 258
IISER PUNE
DSC 258 174 (3.182) AM2 (Ar,20000.0,556.28,0.00,LS 3); ABS; Sm (SG, 1x1.00); Cm (173:180)
1: TOF MS ES+
4.21e6
CALCULATED MASS
(M+Na)+940.3996
Ph
PMBO
OBn
BnO
OBn
N3
11
100
%
0
940.3986
1010.4677
1011.4708
1012.4734
1018.4811
282.2798
256.2646
283.2832
474.2125
665.2746
878.4101
1306.6071
1781.8007
1808.8202
1853.8533
m/z
100
200
300
400
500
600
700
800
900
1000
1100
1200
1300
1400
1500
1600
1700
1800
1900

DSC An IISER PUNE

DSC An 16 (0.308) AM2 (Ar,20000.0,556.28,0.00,LS 3); ABS; Sm (SG, 1x1.00); Cm (16:18-(2:15+19:273))

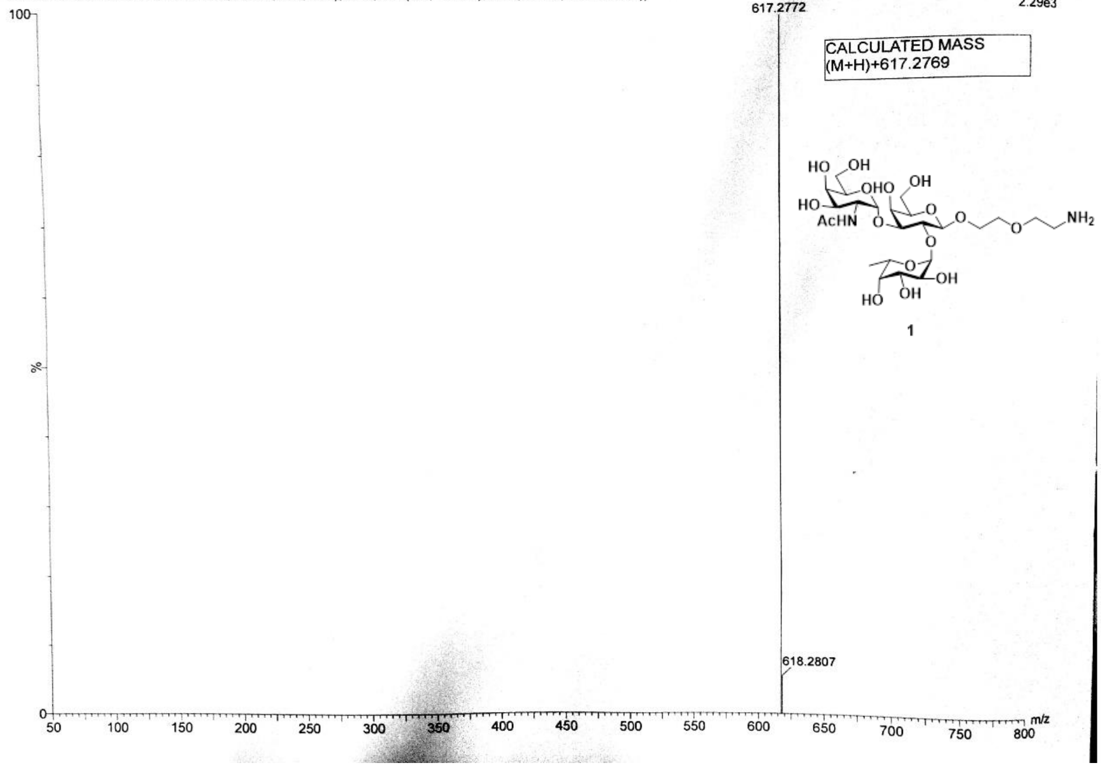

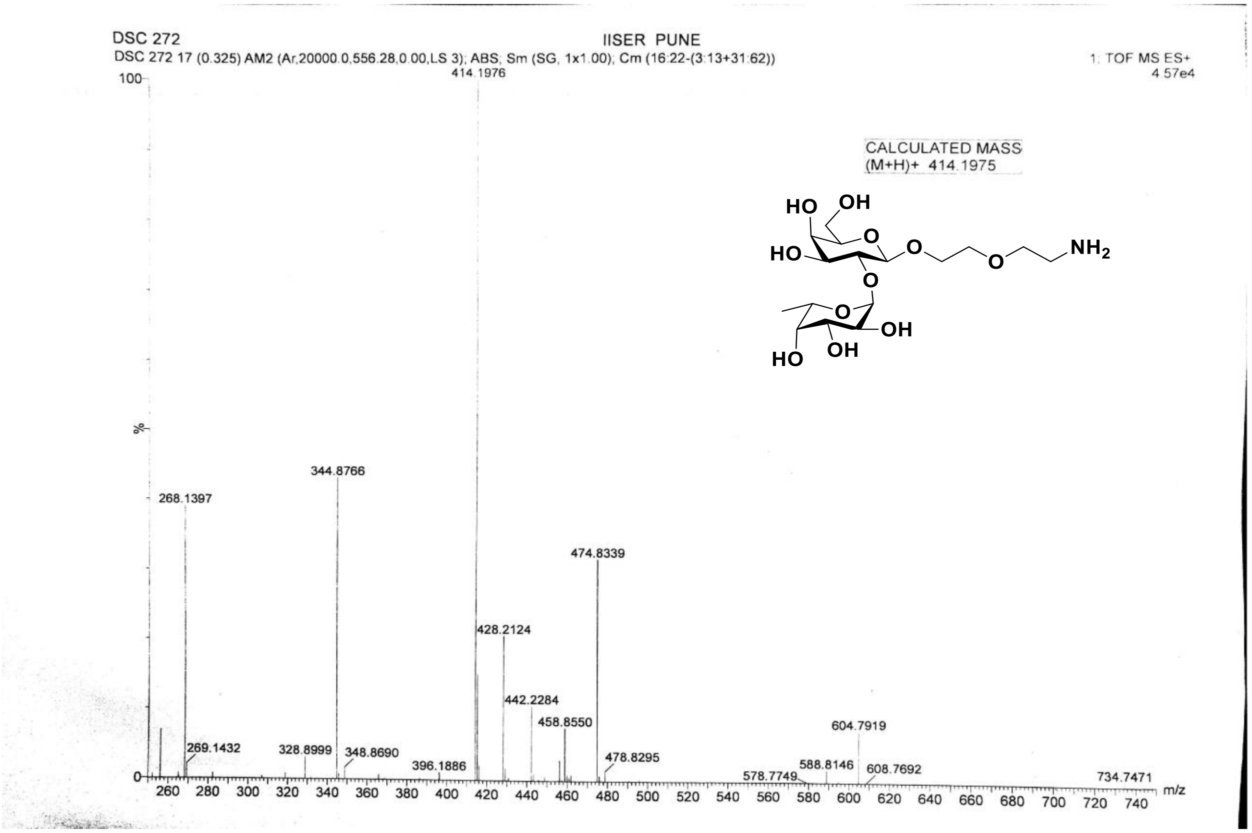
DSC 272
IISER PUNE
DSC 272 17 (0.325) AM2 (Ar,20000.0,556.28,0.00,LS 3); ABS; Sm (SG, 1x1.00); Cm (16:22-(3:13+31:62))
1: TOF MS ES+
4.57e4
100
%
0
414.1976
CALCULATED MASS
(M+H)+ 414.1975
HO
OH
O
HO
O
O
O
NH2
OH
HO
OH
344.8766
268.1397
474.8339
428.2124
442.2284
458.8550
604.7919
269.1432
328.8999
348.8690
396.1886
478.8295
588.8146
578.7749
608.7692
734.7471
m/z
260 280 300 320 340 360 380 400 420 440 460 480 500 520 540 560 580 600 620 640 660 680 700 720 740
